# Cyclohexanone and Phenolic Acid Derivatives from Endophytic Fungus *Diaporthe foeniculina*

**DOI:** 10.3389/fchem.2021.738307

**Published:** 2021-09-01

**Authors:** Xiuxiang Lu, Yanjiang Zhang, Wenge Zhang, Huan Wang, Jun Zhang, Sasa Wang, Haibo Tan

**Affiliations:** ^1^ Key Laboratory of Plant Resources Conservation and Sustainable Utilization, Guangdong Provincial Key Laboratory of Applied Botany, South China Botanical Garden, Chinese Academy of Sciences, Guangzhou, China; ^2^ University of Chinese Academy of Sciences, Beijing, China; ^3^ National Engineering Research Center of Navel Orange, Gannan Normal University, Ganzhou, China; ^4^ Key Laboratory of Chemistry and Engineering of Forest Products, Guangxi University for Nationalities, Nanning, China

**Keywords:** *Diaporthe foeniculina*, *Leptospermum brachyandrum*, foeniculins A-K, cytotoxic activity, antibacterial activity

## Abstract

Chemical investigation of an endophytic fungus *Diaporthe foeniculina* SCBG-15, led to the isolation of eight new cyclohexanone derivatives, foeniculins A–H (1–8) and three new phenolic acid derivatives, foeniculins I–K (9–11). Their structures were extensively established on the basis of ^1^H and ^13^C NMR spectra together with COSY, HSQC, HMBC, and NOESY experiments. The absolute configurations were confirmed by quantum chemical ECD calculations and single-crystal X-ray diffractions. Moreover, the *in vitro* cytotoxic and antibacterial activities of isolated compounds 1–11 were also evaluated.

## Introduction

*Leptospermum brachyandrum* belongs to the genus *Leptospermum*, it is an important member in the plant family Myrtaceae (Beardsell et al., 1993; Brophy et al., 1999). It mainly occurred in Australia and had been introduced into China a few decades ago. Nowadays, this plant is widely planted in the southern of China due to its ornamental and medicinal properties. Our previous phytochemical works proved that the chemical constitutes of *L. brachyandrum* were ploymethylated meroterpenoid and phloroglucinol derivatives (Zou et al., 2018). In recent years, our group focused on bioactive meaningful natural products from the plants and endophytic fungi towards the pharmaceutical drug discovery (Liu et al., 2016a; Liu et al., 2016b; Liu et al., 2016c; Xiang et al., 2017; Liu et al., 2018). As a part of our ongoing research effort to discover biologically active and structurally unique natural products (Liu et al., 2016d; Liu et al., 2016e; Li et al., 2017), the *Diaporthe foeniculinaan* SCBG-15, an endophytic strain derived from *L. brachyandrum*, which displayed a variety of secondary metabolisms with potentially structural diversity during the HPLC and TLC analyses, was selected as the target for the further chemical investigation.

**FIGURE1:**
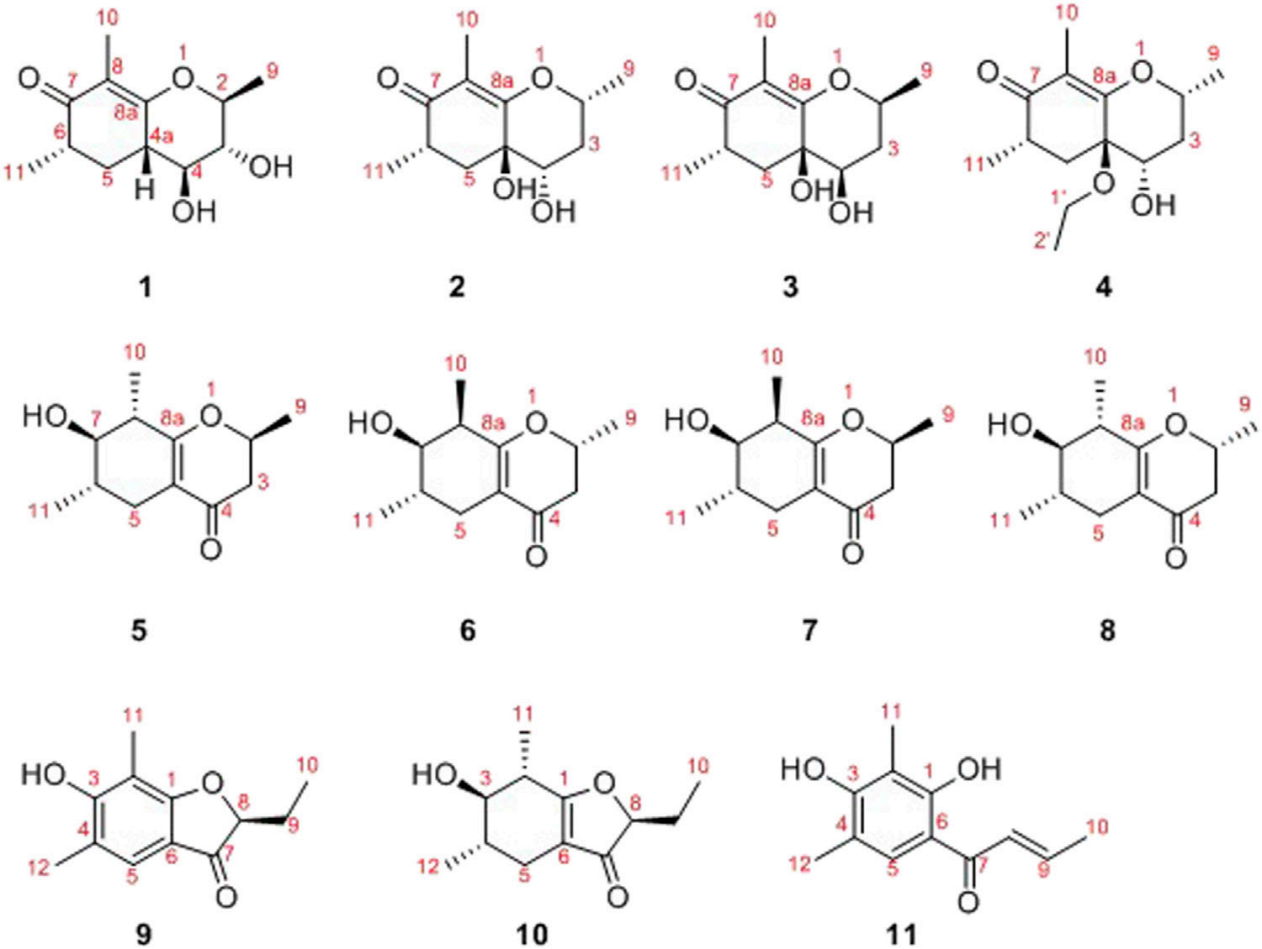
Structures of compounds 1–11.

In the latest years, plenty of new privileged natural compounds with highly structural diversities were isolated from the genus *Diaporthe*, and which exhibited significant biological activities (Zhu et al., 2010; Zang et al., 2012; Li et al., 2015; Mandavid et al., 2015; Cui et al., 2017; Cui et al., 2018; Luo et al., 2018; Gao et al., 2020). In this study, an extensively chemical constituent research on EtOAc extract of the fungus SCBG-15 using sequential column chromatography over silica gel, RP-C_18_ silica, and Sephadex LH-20 along with preparative and semipreparative HPLC resulted in the discovery of eight new cyclohexanone derivatives, foeniculins A–H (1–8), and three phenolic acid derivatives, foeniculins I–K (9–11). All of the novel compounds 1–11 possessed polymethylated skeleton (Figure 1). Herein, the details of isolation, structural elucidation by NMR spectral interpretation, single-crystal X-ray diffraction, and biological evaluation of these isolates are described.

## Materials and Methods

### General Experimental Procedures

Optical rotations were recorded using an Anton Paar MCP-500 spectropolarimeter (Anton Paar, Graz, Austria). UV spectra were obtained by a Shimadzu UV-2600 spectrophotometer (Shimadzu, Kyoto, Japan). ECD spectra were measured with an Applied Photophysis Chirascan. IR data were measured on a Shimadzu IR Affinity-1 spectrometer (Shimadzu, Kyoto, Japan). 1D and ^2^D NMR spectra were collected on a Bruker Avance-500 spectrometer with TMS as an internal standard (Bruker, Fällanden, Switzerland). HRESIMS spectra were acquired with a Thermo MAT95XP high resolution mass spectrometer (Thermo Fisher Scientific, Bremen, Germany). Silica gel (200–300 mesh, Qingdao Marine Chemical Inc. Qingdao, China) was used for column chromatography. TLC analysis was carried out on silica gel plate (Merck KGaA, Darmstadt, Germany). A Hitachi Primaide [Hitachi Instruments (Dalian) Co., Ltd.] equipped with a diode array detector (DAD) using a semi-preparative YMC ODS C_18_ column (20 × 250 mm, 5 μm) was used for semi-preparative HPLC separation. All solvents were analytical grade (Guangzhou Chemical Regents Company, Ltd. Guangzhou, China).

### Fungal Material

The endophytic fungal strain *D. foeniculina* SCBG-15 was isolated from the plant of *L. brachyandrum*, which was collected at South China Botanical Garden (SCBG), Chinese Academy of Sciences, China, in September 2016. The strain was identified by sequence analysis of rDNA ITS (internal transcribed spacer) region. The sequence of the ITS region of the *D. foeniculina* has been submitted to GenBank (Accession No. MN788609). The strain is preserved at the Laboratory of Natural Product Medicinal Chemistry, SCBG.

### Extraction and Isolation

The fungus *D. foeniculina* was fermented on an autoclaved rice solid medium (15 × 3 L Erlenmeyer flasks, each containing 300 g of grains and 360 ml of distilled water) for 30 days at 28°C. After cultivation, the mycelia and rice solid medium were extracted with EtOAc for three times, and the crude extract (50 g) was obtained. The crude extract was subjected to silica gel using gradient elution with petroleum ether-EtOAc as eluent (v/v, 100:1→50:50) and CH_2_Cl_2_-MeOH (v/v, 5:1→2:1). Then, they were combined by TLC analysis to afford six main fractions (Fr.1-Fr.6).

Fr.5 (7.22 g) was applied to column chromatography over RP-C_18_ silica gel, eluting with MeOH-H_2_O (v/v, 2:5→1:0) to give six subfractions (Fr.5-1 to Fr.5-6). Fr.5-2 (1.94 g) was separated by Sephadex LH-20 column chromatography and eluted with CHCl_3_-MeOH (v/v, 1:1) to afford six subfractions (Fr.5-2-1 to Fr.5-2-6). Fr.5-2-2 (1.23 g) was isolated by column chromatography on silica gel and eluted with *n*-hexane-EtOAc gradient (v/v, 4:1→1:5) to obtain six subfractions (Fr.5-2-2-1 to Fr.5-2-2-6). Fr.5-2-2-5 (127.4 mg) was further purified by the semi-preparative HPLC system with CH_3_CN-H_2_O (10:90) as eluent to afford compounds 1 (3.0 mg, t_*R*_ = 35.0 min), 2 (4.4 mg, t_*R*_ = 15.7 min), and 3 (2.0 mg, t_*R*_ = 21.6 min). Fr.5-2-1 (311.4 mg) was isolated by column chromatography on silica gel and eluted with *n*-hexane-EtOAc gradient (v/v, 5:1→1:5) to get three subfractions (Fr.5-2-1-1 to Fr.5-2-1-3). Fr.5-2-1-1 (208.8 mg) was subjected to semi-preparative HPLC with CH_3_CN-H_2_O (v/v, 50:50) to give seven subfractions (Fr.5-2-1-1-1 to Fr.5-2-1-1-7). Fr.5-2-1-1-6 (28.3 mg) was purified by semi-preparative HPLC and washed with CH_3_CN-H_2_O (v/v, 35:65) to afford compound 4 (3.0 mg, t_*R*_ = 20.5 min). Fr.5-2-1-1-1 (23.1 mg) was purified by semi-preparative HPLC equipped with a chiral column and washed with isopropanol-hexane (30:70) to afford compounds 5 (1.0 mg, t_*R*_ = 19.8 min), 6 (1.0 mg, t_*R*_ = 18.5 min), 7 (0.8 mg, t_*R*_ = 22.8 min), and 8 (1.3 mg, t_*R*_ = 28.0 min). Fr.5-2-1-1-2 (40.2 mg) was purified by semi-preparative HPLC and washed with MeOH-H_2_O (v/v, 75:25) to afford compound 10 (3.0 mg, t_*R*_ = 20.5 min).

Fr.4 (2.26 g) was isolated by column chromatography on silica gel and eluted with *n*-hexane-EtOAc gradient (v/v, 30:1→1:1) to get four subfractions (Fr.4-1 to Fr.4-4). Fr.4-2 (197.1 mg) was separated by Sephadex LH-20 column chromatography and eluted with CHCl_3_-MeOH (v/v, 1:1) to afford four subfractions (Fr.4-2-1 to Fr.4-2-4). Fr.4-2-4 (13.8 mg) was further purified by the semi-preparative HPLC system and eluted with MeOH-H_2_O (70:30) to give compound 9 (3.2 mg, t_*R*_ = 19.4 min).

Fr.6 (19.0 g) was separated into four subfractions (Fr.6-1 to Fr.6-4) on ODS column chromatography with MeOH-H_2_O (v/v, 3:10→4:1). Fr.6-1 (2.79 g) was loaded onto Sephadex LH-20 column chromatography and eluted with CHCl_3_-MeOH (v/v, 1:1) to give four subfractions (Fr.6-1-1 to Fr.6-1-4). Fr.6-1-2 (643.0 mg) was isolated by column chromatography on silica gel and eluted with CH_2_Cl_2_-MeOH (v/v, 50:1→1:5) to get seven subfractions (Fr.6-1-2-1 to Fr.6-1-2-7). Fr.6-1-2-4 (311.0 mg) was separated by semi-preparative HPLC with CH_3_CN-H_2_O (v/v, 10:90) and then repeatedly purified by semi-preparative HPLC with CH_3_CN-H_2_O (v/v, 2: 98) to afford compound 11 (5.4 mg, t_*R*_ = 8.7 min).

Foeniculin A (1): colorless needle crystals (*α*)^20^_D_–12.4 (*c* 0.1, MeOH); UV (MeOH): *λ*_max_ (log *ε*): 259 (2.77), 202 (2.41) nm; IR (KBr): 3,381, 2,996, 2,905, 2,837, 1,616, 1,559, 1,456, 1,385, 1,308, 1,229, 1,206, 1,098, 1,024, 695, 758, 733, 667, 596, 556 cm^−1^; HRESIMS: *m*/*z* 227.1274 (M + H)^+^ (calcd for C_12_H_19_O_4_, 227.1278). ^1^H (500 MHz) and ^13^C (125 MHz) NMR data, see Tables 1 and 2.

**TABLE 1:** ^1^H (500 MHz) NMR data for compounds 1–4 (*δ* in ppm, *J* in Hz).

No	1^a^	2^a^	3^a^	4^b^
2	3.73, m	4.62, m	4.42, ddd (2.8, 6.3, 12.4)	4.60, qd (6.3, 11.6)
3*α*	3.29, m	1.60, dd (2.9, 14.3)	1.77, dd (6.3, 14.0)	1.65, m
3*β*		2.54, ddd (5.5, 7.5, 14.3)	2.34, ddd (2.2, 12.4, 14.0)	2.48, ddd, (5.2, 7.5, 14.3)
4	3.26, m	3.78, dd (2.9, 7.5)	3.66, dd (2.2, 3.5)	4.07, dd (2.8, 7.5)
	2.55, m			
5*α*	2.44, m	1.78, dd (4.6, 13.6)	1.72, dd (4.8, 13.0)	2.19, m
5*β*	1.34, m	2.25, dd (6.8, 13.6)	2.21, t 13.0	
6	2.27, m	2.79, ddd (4.6, 6.8, 13.6)	2.75, ddd (4.8, 6.8, 13.0)	2.61, m
9	1.48, d (6.2)	1.38, d (6.3)	1.38, d (6.3)	1.35, d (6.3)
10	1.68, s	1.65, s	1.66, s	1.65, s
11	1.16, d (6.2)	1.12, d (6.3)	1.13, d (6.3)	1.09, d (6.3)
1′				3.60, m
2′				1.16, t (7.0)

a Recorded in CD_3_OD.

b Recorded in CD_3_COCD_3_.

**TABLE 2:** ^13^C (125 MHz) NMR data for compounds 1–4 (*δ*_C_ in ppm).

No	1^a^	2^a^	3^a^	4^b^
1				
2	79.1, CH	71.1, CH	71.9, CH	69.8, CH
3	76.3, CH	39.0, CH_2_	35.5, CH_2_	38.2, CH
4	76.0, CH	70.6, CH	71.0, CH	66.9, CH
4a	43.8, C	70.5, C	69.0, C	75.1, C
5	33.1, CH_2_	40.9, CH_2_	40.3, CH_2_	34.4, CH_2_
6	41.5, CH	36.2, CH	37.6, CH	35.9, CH
7	204.1, C	203.6, C	204.0, C	198.9, C
8	115.9, C	115.2, C	118.0, C	115.2, C
8a	171.0, C	170.1, C	170.8, C	165.5, C
9	18.8, CH_3_	22.6, CH_3_	22.8, CH_3_	21.9, CH_3_
10	8.2, CH_3_	7.9, CH_3_	8.2, CH_3_	7.3, CH_3_
11	15.8, CH_3_	15.7, CH_3_	15.6, CH_3_	15.1, CH_3_
1′				59.3, CH_2_
2′				15.4, CH_3_

a Recorded in CD_3_OD.

b Recorded in CD_3_COCD_3_.

Foeniculin B (2): colorless needle crystals; m. p. 120–121°C (*α*)^20^_D_ + 8.2 (*c* 0.1, MeOH); UV (MeOH): *λ*_max_ (log *ε*): 267 (3.28) nm; IR (KBr): 3,370, 2,976, 2,932, 2,884, 1,717, 1,614, 1,381, 1,337, 1,242, 1,217, 1,146, 1,105, 1,026, 978, 874, 773, 739, 689, 667, 596 cm^−1^; HRESIMS: *m*/*z* 227.1275 (M + H)^+^ (calcd for C_12_H_19_O_4_, 227.1278). ^1^H (500 MHz) and ^13^C (125 MHz) NMR data, see Tables 1 and 2.

Foeniculin C (3): white solid (*α*)^20^_D_–33.4 (*c* 0.1, MeOH); UV (MeOH): *λ*_max_ (log *ε*): 263 (3.10) nm; IR (KBr): 3,377, 2,974, 2,926, 1,616, 1,456, 1,386, 1,333, 1,289, 1,252, 1,209, 1,141, 1,103, 1,068, 1,011, 976, 914, 883, 760, 692 cm^−1^; HRESIMS: *m*/*z* 227.1276 (M + H)^+^ (calcd for C_12_H_19_O_4_, 227.1278). ^1^H (500 MHz) and ^13^C (125 MHz) NMR data, see Tables 1 and 2.

Foeniculin D (4): white solid (*α*)^20^_D_ + 11.6 (*c* 0.05, MeOH); UV (MeOH): *λ*_max_ (log *ε*): 269 (2.54) nm; IR (KBr): 3,329, 2,947, 2,835, 1,651, 1,456, 1,410, 1,115, 1,017, 667, 608, 546 cm^−1^; HRESIMS: *m*/*z* 255.1598 (M + H)^+^ (calcd for C_14_H_23_O_4_, 255.1591). ^1^H (500 MHz) and ^13^C (125 MHz) NMR data, see Tables 1 and 2.

Foeniculin E (5): colorless needle crystals (*α*)^20^_D_–11.6 (*c* 0.03, MeOH); UV (MeOH): *λ*_max_ (log *ε*): 275 (2.98) nm; IR (KBr): 3,356, 1,653, 1,616, 667, 600, 552 cm^−1^; HRESIMS: *m*/*z* 211.1329 (M + H)^+^ (calcd for C_12_H_19_O_3_, 211.1329). ^1^H (500 MHz) and ^13^C (125 MHz) NMR data, see Tables 3 and 4.

**TABLE 3:** ^1^H NMR (500 MHz) data for compounds 5–8 in CD_3_OD (*δ* in ppm, *J* in Hz).^**a**^

No	5	6	7	8
2	4.48, m	4.45, m	4.50, m	4.43, m
3	2.44, ddd (4.9, 8.4, 9.3)	2.43, m	2.43, m	2.41, m
5*α*	2.55, dd (4.9, 15.9)	2.43, m	2.49, dd (5.1, 15.7)	2.26, m
5*β*	1.65, dd (2.5, 11.4, 15.9)	1.73, m	1.80, m	1.95, m
6	1.52, ddd (6.1, 11.1, 17.2)	1.81, m	1.86, m	1.86, m
7	2.98, dd (8.9, 10.2)	3.48, dd (5.5, 9.7)	3.55, dd (5.1, 8.5)	3.57, m
8	2.33, ddd (4.9, 8.4, 9.3)	2.60, m	2.57, m	2.41, m
9	1.42, d (6.3)	1.42, d (6.3)	1.41, d (6.3)	1.42, d (6.3)
10	1.26, d (7.0)	1.17, d (7.2)	1.18, d (7.2)	1.19, d (7.4)
11	1.08, d (6.4)	1.03, d (6.4)	1.01, d (6.5)	1.05, d (6.8)

**TABLE 4:** ^13^C (125 MHz) NMR data for compounds 5–8 in CD_3_OD (*δ* in ppm).

No	5	6	7	8
1				
2	75.2, CH	75.1, CH	74.8, CH	76.5, CH
3	42.2, CH_2_	41.9, CH_2_	42.1, CH_2_	43.7, CH_2_
4	194.0, C	193.4, C	193.8, C	195.4, C
4a	109.2, C	109.8, C	108.7, C	111.1, C
5	27.4, CH_2_	27.0, CH_2_	26.2, CH_2_	25.3, CH_2_
6	29.3, CH	35.2, CH	30.0, CH	29.6, CH
7	73.1, CH	78.0, CH	73.0, CH	75.6, CH
8	38.4, CH	42.2, CH	37.7, CH	42.7, CH
8a	174.2, C	173.0, C	173.9, C	175.2, C
9	19.3, CH_3_	19.0, CH_3_	19.1, CH_3_	20.8, CH_3_
10	16.5, CH_3_	13.7, CH_3_	11.3, CH_3_	17.2, CH_3_
11	11.2, CH_3_	16.6, CH_3_	16.3, CH_3_	17.4, CH_3_

Foeniculin F (6): white solid (*α*)^20^_D_ + 12.0 (*c* 0.05, MeOH); UV (MeOH); *λ*_max_ (log *ε*): 268 (3.34), 202 (2.23) nm; HRESIMS: *m*/*z* 233.1146 (M + Na)^+^ (calcd for C_12_H_18_NaO_3_, 233.1148). ^1^H (500 MHz) and ^13^C (125 MHz) NMR data, see Tables 3 and 4.

Foeniculin G (7): white solid (*α*)^20^_D_–13.7 (*c* 0.05, MeOH); UV (MeOH): *λ*_max_ (log *ε*): 275 (3.08) nm; IR (KBr): 3,337, 1,636, 669, 600, 554 cm^−1^; HRESIMS: *m*/*z* 211.1339 (M + H)^+^ (calcd for C_12_H_19_O_3_, 211.1329). ^1^H (500 MHz) and ^13^C (125 MHz) NMR data, see Tables 3 and 4.

Foeniculin H (8): white solid (*α*)^20^_D_ + 10.8 (*c* 0.1, MeOH); UV (MeOH): *λ*_max_ (log *ε*): 274 (3.23) nm; IR (KBr): 3,360, 1,636, 667, 600, 557 cm^−1^; HRESIMS: *m*/*z* 211.1339 (M + H)^+^ (calcd for C_12_H_19_O_3_, 211.1329). ^1^H (500 MHz) and ^13^C (125 MHz) NMR data, see Tables 3 and 4.

Foeniculin I (9): colorless needle crystals; m. p. 108–109°C; UV (MeOH): *λ*_max_ (log *ε*): 328 (3.02), 279 (3.33), 217 (3.47) nm; IR (KBr): 3,379, 2,976, 2,922, 2,851, 1,676, 1,608, 1,458, 1,329, 1,292, 1,220, 1,151, 1,092, 1,024, 947, 768 cm^−1^; HRESIMS: *m*/*z* 207.1013 (M + H)^+^ (calcd for C_12_H_15_O_3_, 207.1016). ^1^H (500 MHz) and ^13^C (125 MHz) NMR data, see Table 5.

**TABLE 5:** ^1^H (500 MHz) and^13^C (125 MHz) NMR data (*δ* in ppm, *J* in Hz) of 9–11.

No	9^c^		10^a^		11^a^	
	*δ*_H_ (*J* in Hz)	*δ*_C_	*δ*_H_ (*J* in Hz)	*δ*_C_	*δ*_H_ (*J* in Hz)	*δ*_C_
1		172.0, C		189.4, C		164.0, C
2		106.8, C	2.70, m	38.6, CH		112.4, C
3		160.5, C	3.73, br s	76.0, CH		162.5, C
4		118.7, C	2.02, m	29.9, CH		117.5, C
5	7.29, s	122.4, CH	2.28, m; 2.02, m	21.6, CH_2_	7.53, s	130.6, CH
6		113.7, C		111.1, C		113.7, C
7		200.6, C		203.1, C		194.1, C
8	4.50, dd (4.5, 7.1)	87.0, CH	4.42, dd (4.5, 6.8)	86.9, CH	7.14, d (15.6)	127.4, CH
9	2.05, m; 1.80, m	24.8, CH_2_	2.02, m; 1.75, m	24.4, CH_2_	7.08, dq (15.6, 5.5)	145.1, CH
10	1.00, t (7.4)	8.9, CH_3_	0.96, t (7.4)	8.5, CH_3_	2.00, d (5.5)	18.8, CH_3_
11	2.19, s	7.3, CH_3_	1.30, d (7.3)	15.8, CH_3_	1.07, s	8.3, CH_3_
12	2.23, s	15.8, CH_3_	1.10, d (6.7)	16.2, CH_3_	2.18, s	16.6, CH_3_

a Recorded in CD_3_OD.

b Recorded in CD_3_COCD_3_.

c Recorded in CDCl_3_.

Foeniculin J (10): colorless oil (*α*)^20^_D_–11.4 (*c* 0.05, MeOH); UV (MeOH): *λ*_max_ (log *ε*): 270 (2.80) nm; IR (KBr): 3,312, 2,976, 2,930, 2,899, 1,717, 1,668, 1,607, 1,456, 1,400, 1,344, 1,271, 1,246, 1,180, 1,130, 1,032, 945, 856, 764, 669 cm^−1^; HRESIMS: *m*/*z* 211.1333 (M + H)^+^ (calcd for C_12_H_19_O_3_, 211.1329). ^1^H (500 MHz) and ^13^C (125 MHz) NMR data, see Table 5.

Foeniculin K (11): green needle crystals; UV (MeOH): *λ*_max_ (log *ε*): 305 (3.00) nm; IR (KBr): 3,358, 2,974, 2,920, 1,649, 1,626, 1,560, 1,479, 1,445, 1,362, 1,305, 1,290, 1,169, 1,113, 1,028, 953, 829 cm^−1^; HRESIMS: *m*/*z* 207.1025 (M + H)^+^ (calcd for C_12_H_15_O_3_, 207.1016). ^1^H (500 MHz) and ^13^C (125 MHz) NMR data, see Table 5.

### X-Ray Crystallographic Analysis

The single-crystal X-ray diffraction data were collected at 100 K for 1, 2, 5, and 9 on Agilent Xcalibur Nova single-crystal diffractometer using CuKα radiation. Crystallographic data for 1, 2, 5, and 9 reported in this paper have been deposited in the Cambridge Crystallographic Data Centre. (Deposition number: CCDC 2008519 for 1, 2008520 for 2, 2047671 for 5, and 2047672 for 9). Copies of these data can be obtained free of charge *via* www.ccdc.cam.au.ck/conts/retrieving.html.)

### Cytotoxicity Assay

The *in vitro* cytotoxic activities of compounds 1–11 were assayed against three human tumor cell lines SF-268, MCF-7, HePG-2, and normal cell line LX-2 with adriamycin as positive control. Assays were performed by the SRB method (Mosmann, 1983).

### Antimicrobial Assay

Compounds 1–11 were evaluated the antimicrobial activity against *Staphylococcus aureus* (CMCC 26003) and *Escherichia coli* (ATCC 8739). Assays were performed by the published microdilution method for the estimation of minimum inhibitory concentration (MIC) values (Li et al., 2017). Vancomycin was used as positive control.

## Results and Discussion

Compound 1 was isolated as needle crystals. Its molecular formula of C_12_H_18_O_4_ was established on the basis of (+)-HRESIMS *m*/*z* 227.1274 (M + H)^+^ (calcd for C_12_H_19_O_4_, 227.1278), implying four degrees of hydrogen deficiency. The IR spectrum of 1 logically revealed the presence of carbonyl and free hydroxyl functional groups through the characteristic resonance absorptions at 1,616 and 3,381 cm^−1^, respectively. The ^1^H NMR data (Table 1) of 1 exhibited a series of typical proton signals, which were responsive for three oxygenated methines [*δ*_H_ 3.73 (1H, m, H-2), 3.29 (1H, m, H-3), 3.26 (1H, m, H-4)] and three methyl moieties [*δ*_H_ 1.48 (3H, d, *J* = 6.2 Hz, H-9), 1.68 (3H, s, H-11), 1.16 (3H, d, *J* = 6.3 Hz, H-10)]. The ^13^C NMR data (Table 2) combined with HSQC spectrum of 1 resolved 12 carbon resonances attributable to three methyls, one methylene, four methines, and four quaternary carbons including one carbonyl functionality (*δ*_C_ 204.1).

**FIGURE 2:**
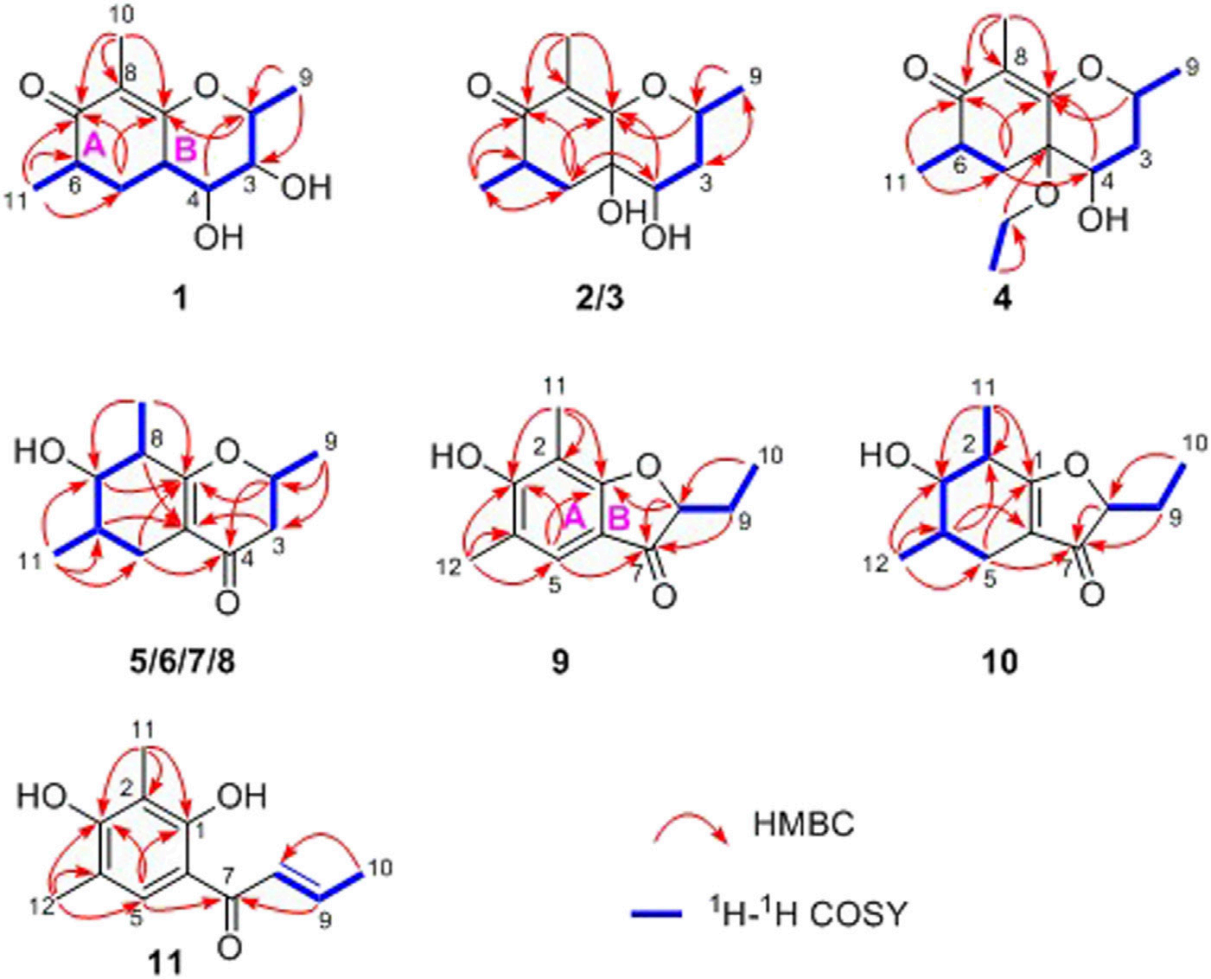
Key ^1^H-^1^H COSY and HMBC correlations of compounds 1–11.

In the ^1^H-^1^H COSY spectrum (Figure 2), the cross peaks of H_3_-11/H-6/H_2_-5 suggested the presence of fragment a (C-11/C-6/C-5). The HMBC correlations from H_3_-10 to C-5 (*δ*_C_ 33.1), C-6 (*δ*_C_ 41.5), and C-7 (*δ*_C_ 204.1), H-11 to C-7, C-8 (*δ*_C_ 115.9), and C-8a (*δ*_C_ 171.0), H_2_-5 to C-7 and C-8a coupled with the fragment a were significantly suggested the existence of a cyclohexanone ring (ring A) with a carbonyl group located at C-7 position as well as two methyls attached at C-6 and C-8 positions, respectively. In addition, the obvious HMBC correlations from H-2 to C-4 (*δ*_C_ 76.0) and C-8a, H-9 to C-2 (*δ*_C_ 79.1) and C-3 (*δ*_C_ 76.3) together with the ^1^H-^1^H COSY spin system b (C-4a/C-4/C-3/C-2/C-9) confirmed the presence of the pyran ring B. Therefore, the planar structure of 1 was established as shown in Figure 1.

**FIGURE 3:**
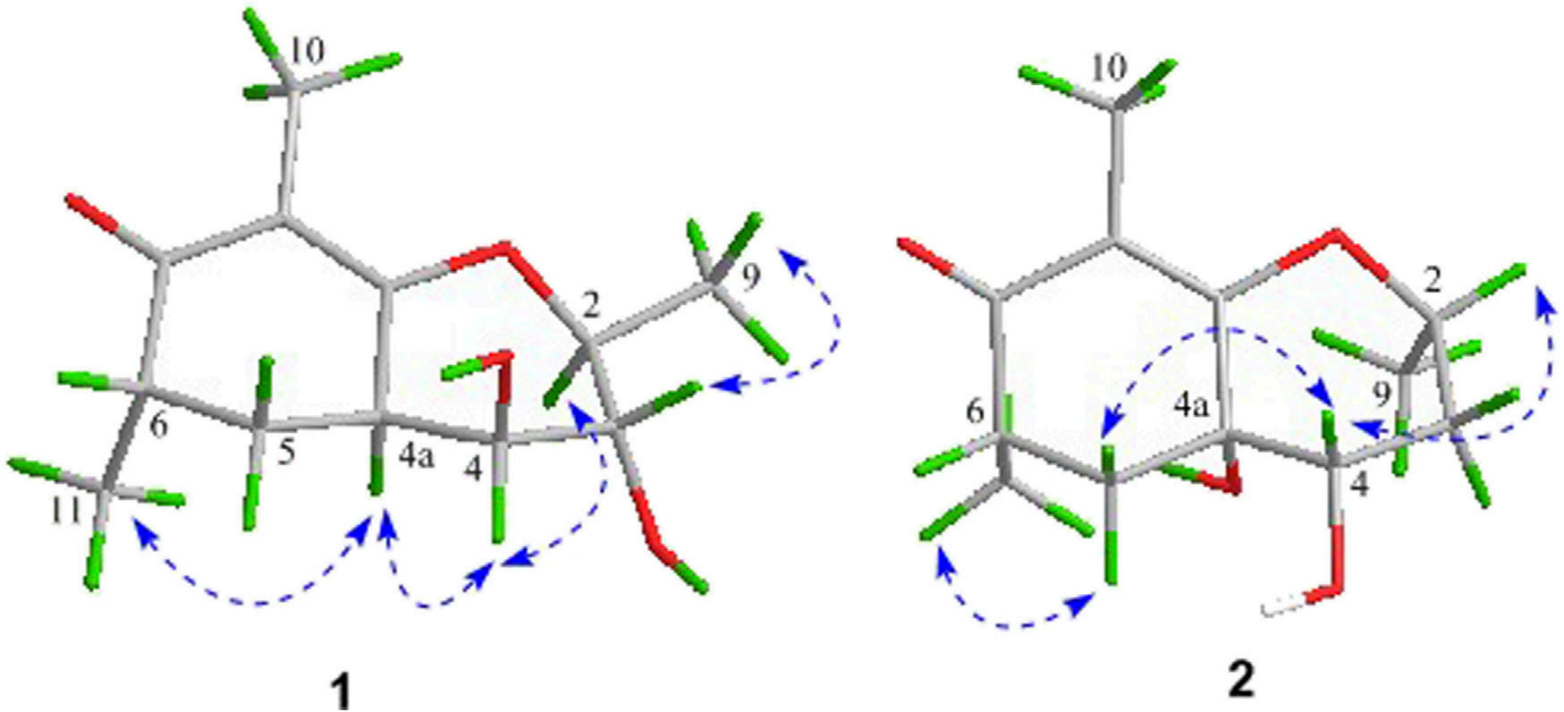
Key NOESY correlations of compounds 1 and 2.

**FIGURE 4:**
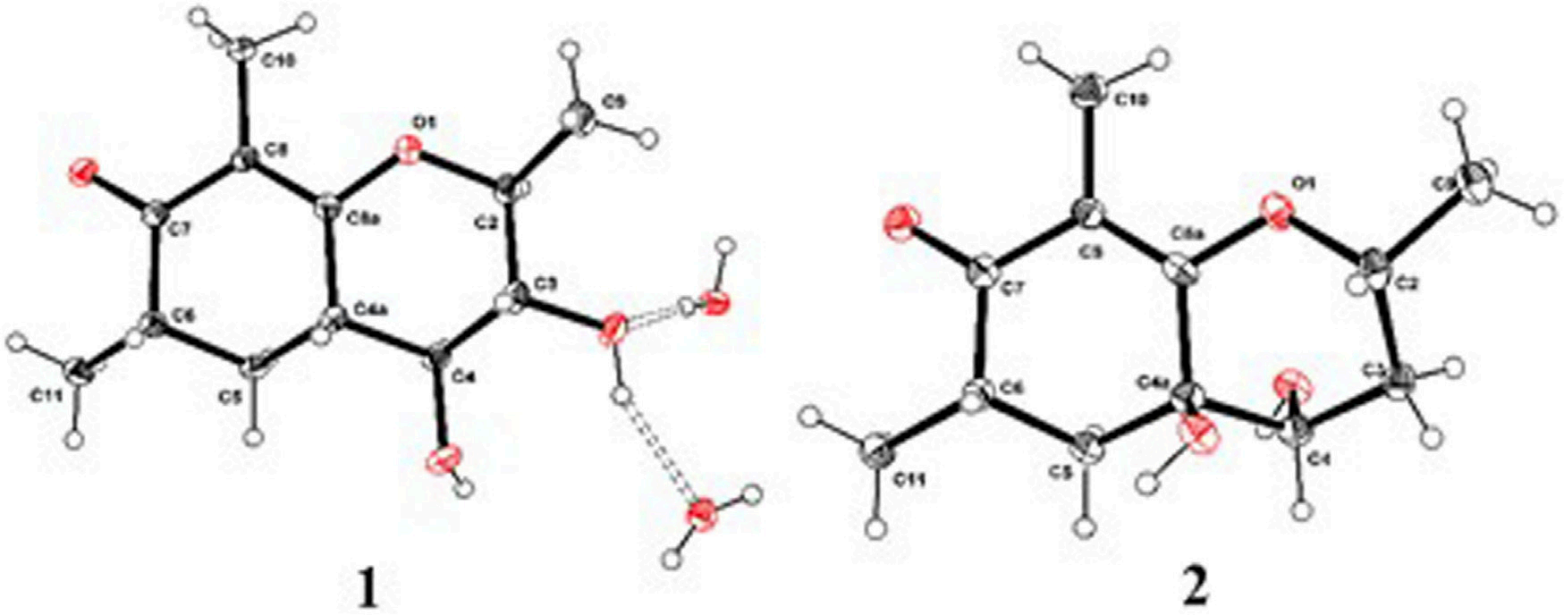
ORTEP drawings of the X-ray structures for compounds 1 and 2.

**FIGURE 5:**
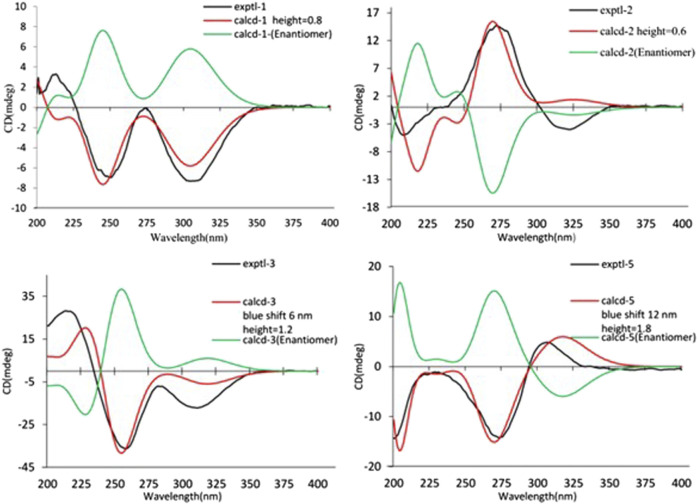
Experimental and calculated ECD spectra of 1–3, 5.

As shown in Figure 3, key NOE correlations of H-2/H-4, H-4/H-4a, H-4a/H_3_-11 confirmed these protons were co-facial, and assigned as *α-*oriented. Then, the NOE correlation between H-5 and H_3_-9 indicated that the methyl group at C-9 was *β-*oriented (Figure 3). Therefore, the relative configuration of 1 was established. The absolute configuration of 1 was finally determined by the single-crystal X-ray diffraction experiment (Figure 4), and it provided the perfect evidence for the absolute configuration of 1 with a Flack parameter of 0.02 (5). Moreover, this conclusion was also verified by the ECD calculations (Figure 5). Therefore, the structure elucidation of compound 1 was completely finished, and its absolute structure was deduced to be 2*S*,3*R*,4*S*,4a*S*,6*S* and trivially named as foeniculin A.

Compound 2 was obtained as needle crystals. Its molecular formula was established as C_12_H_18_O_4_ on the basis of the protonated molecule peak at *m/z* 227.1275 (M + H)^+^ in its HRESIMS spectrum, requiring four degrees of unsaturation. The 1D NMR data (Tables 1 and 2) of 2 were almost in accordance with those of 1, except for the lack of a hydroxyl group at C-3 position in 2, which could be further strengthened by the ^1^H-^1^H COSY cross peaks of H-2/H-3/H-4 as well as the predominant HMBC correlations from H_3_-9 to C-2 and C-3 as well as carbon shit of C-3 (*δ*_C_ 39.0).

The relative configuration of 2 was established by the NOESY experiment. The obvious NOESY cross-peak of H_3_-11 with H-5*α* indicated that these protons should be co-facial, and they were tentatively assigned as *α-*oriented. Moreover, H-5*β* exhibited a conclusive NOESY correlation with H-4, which further correlated with H-2, thus strongly suggesting that they should be located as *β*-oriented (Figure 3). Notably, the relative configuration of the hydroxyl group at C-4 was not determined because of the lack of critical hydroxyl proton signal. Fortunately, the absolute configuration of 2 was successfully determined to be 2*R*,4*S*,4a*R*,6*S* by the analysis of X-ray diffraction data using CuK*α* radiation (Figure 4) and ECD calculation (Figure 5). Therefore, the configuration of 2 was conclusively assigned as shown in Figure 1 and given the trivial name foeniculin B.

**FIGURE 6:**
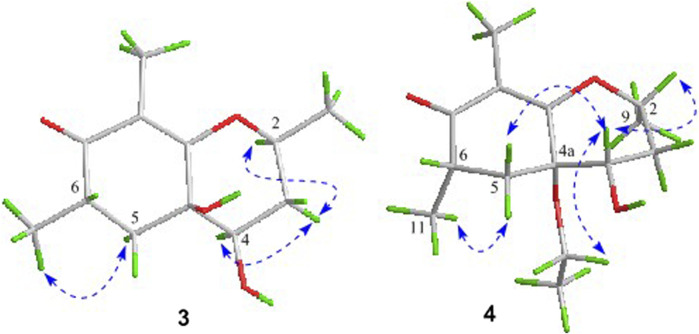
Key NOESY correlations of compounds 3 and 4.

Compound 3 was also obtained as a white amorphous powder with the same molecular formula C_12_H_18_O_4_ as that of 2. The ^1^H NMR data of 3 (Table 1) were closely related to those of 2, only slight differences could be distinguished between the chemical shifts of H-2 (*δ*_H_ 4.62 for 2; *δ*_H_ 4.42 for 3), H-3 (*δ*_H_ 2.54 and 1.60 for 2; *δ*_H_ 2.34 and 1.77 for 3), and H-4 (*δ*_H_ 3.78 for 2; *δ*_H_ 3.66 for 3). Comparing the ^13^C NMR spectra of 2 and 3, the signals attributed to the methylene C-3 (*δ*_C_ 39.0 for 2, *δ*_C_ 35.5 for 3) and quaternary carbon C-8 (*δ*_C_ 115.2 for 2, *δ*_C_ 118.0 for 3) indicated that they should be a pair of diastereoisomers, which showed a little structural difference on the ring B. Interestingly, the partial relative configuration of 3 was determined by NOESY experiment (Figure 6). The NOESY correlations from H-3*β* to H-2 and H-4 assigned these protons as cofacial, thus, the related methyl and hydroxyl functionalities were suggestively established to be *α-*oriented on the ring B. However, the relative configuration of 4a-OH was failed to be determined for the lack of any valuable correlation in the NOESY spectrum. Then, the ECD calculations were employed to establish the absolute configurations of the two diastereoisomers. By fitting the experimental and calculated ECD curves, the 2*S*,4*R*,4a*R*,6*S*-configuration was elucidated for 3 (Figure 5).

Compound 4 was isolated as white solid. Its molecular formula of C_14_H_22_O_4_ was established on the basis of HRESIMS *m*/*z* 255.1598 (M + H)^+^ (calcd for C_14_H_23_O_4_, 255.1591), implying four degrees of hydrogen deficiency. After a careful inspection of the NMR spectra of 4 with those of 2, it could be readily disclosed that they showed very close similarity in most NMR profiles. The major difference between them was the hydroxyl group at C-4a in 2 replaced by a hydroxyethyl one in 4, which could be substantiated by its chemical shifts [*δ*_H_ 3.60 (2H, m), *δ*_C_ (59.3); *δ*_H_ 1.16 (3H, t, *J* = 7.0 Hz), *δ*_C_ (15.4)] in conjunct with the HMBC correlation from H_2_-1′ to C-4a and the ^1^H-^1^H COSY fragment H_2_-1'/H_3_-2'. Interestingly, compound 4 showed an ECD spectrum almost consistent with that of 2 (see Supplementary Material), which strongly illustrated that 4 should also share the similar absolute configuration by the consideration of the same biogenesis. Therefore, the structure of 4 was elucidated as shown in Figure 1 and named as foeniculin D.

Compound 5 was obtained as colorless needle-like crystals. The HRESIMS of compound 5 showed a positive molecular ion peak at *m*/*z* 211.1329, corresponding to a molecular formula of C_12_H_18_O_3_. The ^1^H NMR (Table 3) data of 5 exhibited a series of characteristic proton signals, which were responsive for two oxygenated methines [*δ*_H_ 4.48 (1H, m, H-2), 2.98 (1H, m, H-7)] and three methyl groups [*δ*_H_ 1.42 (3H, d, *J* = 6.3 Hz, H-9), 1.26 (3H, d, *J* = 7.0 Hz, H-10), 1.08 (3H, d, *J* = 6.4 Hz, H-11)]. The ^13^C NMR spectrum combined with HSQC data of 5 resolved 12 carbon resonances, and they were attributable to three methyls, two methylenes, four methines, and three quaternary carbons including a carbonyl group (*δ*_C_ 193.4).

**FIGURE 7:**
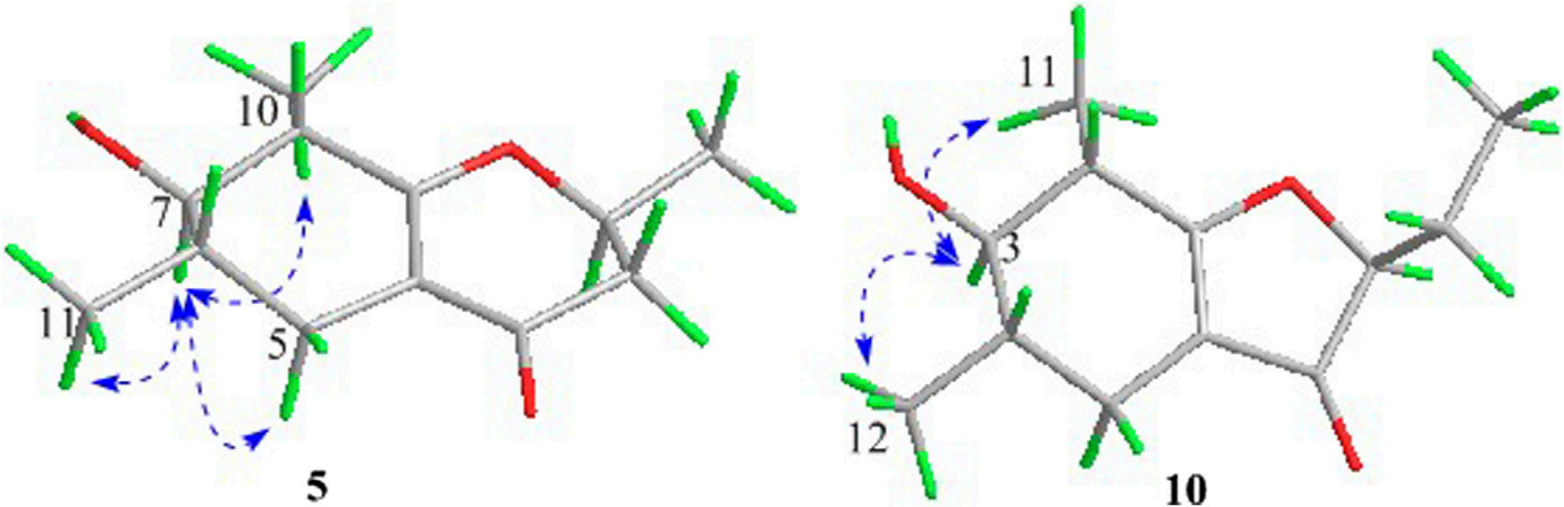
Key NOESY correlations of compounds 5 and 10.

**FIGURE 8:**
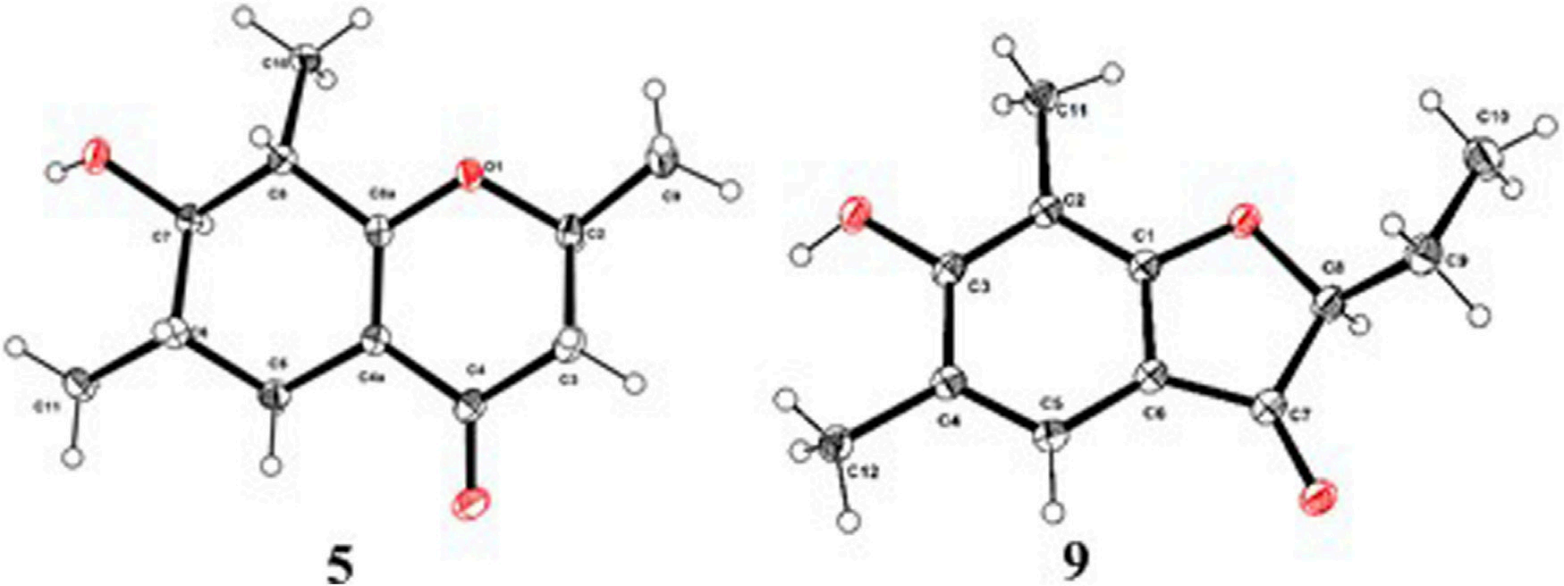
ORTEP drawings of the X-ray structures for compounds 5 and 9.

In the ^1^H-^1^H COSY spectrum (Figure 2), the cross peaks of H_3_-10/H-8/H-7/H-6/H_3_-11 and H_2_-3/H-2/H_3_-9 suggested the presence of two independent fragments, a (C-11/C-6/C-7/C-8/C-10) and b (C-2/C-3/C-9). Based on the fragment a, the HMBC correlations from H-8 to C-6, C-7, and C-4a, H_3_-11 to C-5, C-6, and C-7, H_3_-10 to C-7, C-8, and C-8a suggested the existence of a cyclohexene ring A, which possessed a hydroxyl group located at C-7 and two methyls attached at C-6 and C-8, respectively. Furthermore, the obvious HMBC correlations from H-2 to C-4, H-9 to C-2 and C-3, H-3 to C-4a as well as the ^1^H-^1^H COSY fragment b confirmed the presence of the pyran ring B. The NOESY correlations from H-7 to H-5α, H_3_-10, and H_3_-11 assigned these protons as *β*-orientation (Figure 7). A single crystal of 5 was obtained in MeOH for X-ray diffraction analysis with Flack parameter of 0.04 (9), which suggested the absolute configuration of 5 to be 2*S*,6*S*,7*R*,8*S* shown in Figure 8. Thus, compound 5 was defined as (2*S*,6*S*,7*R*,8*S*)-7-hydroxy-2,6,8-trimethyl-2,3,5,6,7,8-hexahydro-4*H*-chromen-4-one and given the trivial name foeniculin E.

The HRESIMS data *m*/*z* 233.1146 [(M + Na)^+^, calcd C_12_H_18_NaO_3_ 233.1148] of 6, 211.1339 (M + H)^+^ (calcd for C_12_H_19_O_3_, 211.1334) of 7, and *m*/*z* 211.1339 (M + H)^+^ (calcd for C_12_H_19_O_3_, 211.1334) of 8 indicated that compounds 7 and 8 should share the same molecular formula with C_12_H_18_O_3_ as that of 6. Careful comparison of the ^1^H and ^13^C NMR spectra of 6–8 (Tables 3 and 4) with those of foeniculin E (5) revealed that they shared the same planar structure. Moreover, the ^2^D NMR correlations of them (Figure 2) further strengthened this conclusion. Therefore, the aforementioned information suggested that the novel compounds 6–8 should be a series of closely related diastereoisomers of 5.

**FIGURE 9:**
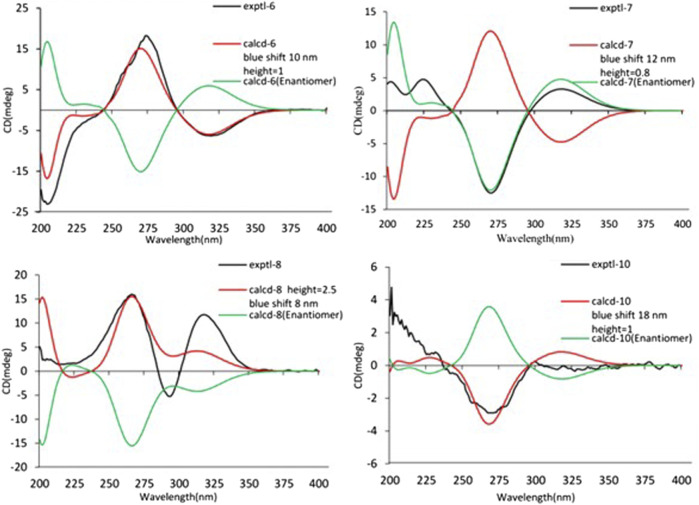
Experimental and calculated ECD spectra of 6–8 and 10.

The relative configuration of 6 was determined by NOESY experiments. In the NOESY spectrum, the obvious NOESY correlations of H-2/H_3_-10, H-6/H_3_-10, and H-7/H_3_-11 indicated the *α*-orientation of H_3_-11 as well as *β*-orientation of H-2, H-6, H_3_-10 and 7-OH. Furthermore, the ECD calculation results showed that the absolute configuration of 6 was 2*R*,6*S*,7*R*,8*R* (Figure 9). Therefore, the structure of 6 was established as shown in Figure 1 and given the trivial name foeniculin F.

Compound 7 shared the same planar structure as those of 5 and 6. In its NOESY spectrum, the key NOESY correlations between H-6/H_3_-10 and H-7/H_3_-11 were readily discovered, which thus successfully established the relative configuration of B ring. However, the lack of the critical NOESY correlations from the protons of A ring to those of B ring made the determination of the absolute configuration of 7 bleak. In order to solve this intractable problem, the ECD calculation method was then performed. Finally, the close comparison of the experimental and calculated ECD curves (Figure 9) revealed the absolute configuration of 7 as 2*S*,6*S*,7*R*,8*R*. Collectively, compound 7 was finally permitted to assign as (2*S*,6*S*,7*R*,8*R*)-7-hydroxy-2,6,8-trimethyl-2,3,5,6,7,8-hexahydro-4H-chromen-4-one and given the trivial name foeniculin G.

Compound 8 also shared very close similarity in the NMR data to those of 6. These subtle differences indicated that the methyl group at C-6 adopted an *α*-orientation and the hydroxyl group at C-7 should be *β*-orientation. This deduction was consistent with the analysis of the ECD calculations (Figure 9). Thus, the absolute structure of compound 8 was determined to be (2*R*,6*S*,7*R*,8*S*)-7-hydroxy-2,6,8-trimethyl-2,3,5,6,7,8-hexahydro-4H-chromen-4-one and given the trivial name foeniculin H.

Compound 9 was isolated as colorless needle crystals, and the molecular formula of C_12_H_14_O_3_ was deduced from the HRESIMS peak at *m*/*z* 207.1013 (M + H)^+^ (calcd for C_12_H_15_O_3_, 207.1021), which clearly suggested the presence of six indices of unsaturation. ^1^H NMR data of 9 (Table 5) revealed three methyl groups including two benzyl protons (*δ*_H_ 2.19 and 2.23, each s), an oxymethine (*δ*_H_ 4.50, dd, *J* = 4.5, 7.1 Hz), a methylene (*δ*_H_ 1.80, m), and an olefinic methine (*δ*_H_ 7.29, s). The ^13^C NMR data (Table 5) and the HSQC spectra revealed the presence of 12 carbons, which included six olefinic carbons (*δ*_C_ 106.8, 113.7, 118.7, 122.4, 160.5, and 172.0), a ketocarbonyl (*δ*_C_ 200.6), three methyls (*δ*_C_ 7.3, 8.9, 15.8), and one oxymethine (*δ*_C_ 87.0). The ^1^H-^1^H COSY revealed one spin-spin system (C-8/C-9/C-10). The HMBC correlations of H-5 to C-1, C-3, C-7, and C-12, H_3_-12 to C-3, C-4, and C-5, as well as H_3_-11 to C-1, C-2, and C-3 established a 3-hydroxy-2,4-dimethylph-2-en-1-one core scaffold for ring A (Figure 1). The HMBC correlations of H-8 to C-1 and C-7 together with H-5 to C-7 established the 5-membered ring B, which fused with ring A at C-1 and C-6 with an ethyl group at C-8. Thus, the planar structure of 9 was successfully established. The 8*S* absolute configuration of 9 was assigned by the X-ray diffraction (Figure 8). Finally, the absolute structure of compound 9 was determined to be (*S*)-8-ethyl-3-hydroxy-2,4-dimethylbenzofuran-3(2H)-one and given the trivial name foeniculin I.

Compound 10 was isolated as a white oil. The molecular formula was established as C_12_H_18_O_3_ from the (M + H)^+^ ion at *m*/*z* 211.1333 in HRESIMS data (calcd for C_12_H_19_O_3_, 211.1329). The molecular unsaturation together with the ^1^H and ^13^C NMR data (Table 5) suggested that 10 was a hydrogenated derivative of 9 with the aid of the HSQC spectrum. The planar structure of 10 was determined unambiguously by ^2^D NMR analyses (^1^H-^1^H COSY, HSQC, and HMBC). The partially relative configuration of 10 was established by analyses of NOESY correlations. The key NOESY correlations between H-3/H_3_-11 and H-3/H_3_-12 strongly suggested that these two methys should be in the same orientation (Figure 7). With its potential biogenesis from the biosynthetic precursor 9, the absolute configuration of C-8 in 10 was rationally deduced to be *S* configuration, which thus resulted the structure of 10 to be 2*R*,3*S*,4*S*,8*S* or 2*S*,3*R*,4*R*,8*S*. Therefore, the calculated ECD methodology was conducted to reveal the possible structure of 10. Fortunately, the calculated ECD spectrum of (2*S*,3*R*,4*S*,8*S*)-10 showed a negative Cotton effect at 270 nm, which well matched with that of the experimental result (Figure 9), allowing the absolute configuration of 10 as 2*S*,3*R*,4*S*,8*S*. Thus, the structure of compound 10 was finally determined and given the trivial name foeniculin J.

According to HRESIMS data, foeniculin K (11) was found to have a molecular formula of C_12_H_14_O_3_, which was the same as that of 9. Analyses of the 1D and 2D NMR of 9 and 11 revealed that compound 11 also possessed a penta-substituted benzene ring A, which was similar to that in compound 9. The main difference between them located in the ring B. In which, compound 11 shared an *α,β-*unsaturated crotonoyl moiety substituted at the C-6 position. This conclusion could be further verified by the ^1^H-^1^H COSY fragment C-8/C-9/C-10 and HMBC correlations from H-5 to C-7. At last, the structure of 11 was determined as shown in Figure 1.

The isolated compounds 1–11 were tested *in vitro* cytotoxic activity against the tumor cell lines SF-268, MCF-7, HePG-2, and normal cell line LX-2. As a result, compound 11 exhibited mild cytotoxicity against the tumor cell line with IC_50_ values of 27.73, 42.54, and 25.12 µM. Compounds 1–10 were inactive to the tested tumor cell lines even at a concentration of 100 µM. The antimicrobial activity of compounds 1–11 was also evaluated against the bacteria *Escherichia coli* and *S. aureus*. However, all of them were found to be devoid of significant activity.

## Conclusion

A phytochemical investigation on the *Diaporthe foeniculina* SCBG-15 resulted in the isolation and structural elucidation of eleven new compounds foeniculins. The structures including absolute configurations were determined by extensive physicochemical and spectroscopic analysis, as well as ECD calculation and X-ray diffraction crystallography. All the novel compounds 1–11 possessed polymethylated skeleton. Compound 11 exhibited cytotoxic activity against the tumor cell lines SF-268, MCF-7, HePG-2 with IC_50_ values of 27.73, 42.54, 25.12 µM, which might serve as a promising antitumor lead compound for the drug discovery.

## Data Availability

All datasets generated for this study are included in the article/Supplementary Material.
