# Supplementary material for: Cyclohexanone and Phenolic Acid Derivatives from Endophytic Fungus Diaporthe foeniculina
Source: Front Chem. 2021 Sep 1;9:738307. doi: 10.3389/fchem.2021.738307 (PMC8440800; doi:10.3389/fchem.2021.738307)
Supplement: Supplementary file 1 [file DataSheet1.PDF]

Cyclohexanone and Phenolic Acid Derivatives from Endophytic Fungus *Diaporthe foeniculina*

Xiuxiang Lu[1,4], Yanjiang Zhang[1,4], Wenge Zhang[1,4], Huan Wang[1,2], Jun Zhang[2], Sasa Wang[3*], Haibo Tan[1,2*]

[1]Key Laboratory of Plant Resources Conservation and Sustainable Utilization, Guangdong Provincial Key Laboratory of Applied Botany, South China Botanical Garden, Chinese Academy of Sciences, Guangzhou, China

[2]National Engineering Research Center of Navel Orange, Gannan Normal University, Ganzhou, China

[3]Key Laboratory of Chemistry and Engineering of Forest Products, Guangxi University for Nationalities, Nanning, China

[4]University of Chinese Academy of Sciences, Beijing 100049, China

[*]Corresponding authors. Tel/Fax: +86-20-37252958 (H. B. Tan)
E-mail address: wgsasa@163.com (S. S.W.); tanhaibo@scbg.ac.cn (H. B. Tan).

Contents

X-ray crytallographic data of compounds **1**, **2**, **5**, and **9**. 6
Figure S1. ^{1}H NMR spectrum (500 MHz, CD_3OD) of **1** 10
Figure S2. ^{13}C NMR Spectrum (125 MHz, $CDCl_3$) of **1** 10
Figure S3. ^{1}H-^{1}H COSY spectrum of **1** 11
Figure S4. HSQC spectrum of **1** 11
Figure S5. HMBC spectrum of **1** 12
Figure S6. NOESY spectrum of **1** 12
Figure S7. HRESIMS spectrum of **1** 13
Figure S8. UV spectrum of **1** 13
Figure S9. CD spectrum of **1** 14
Figure S10. IR spectrum of **1** 14
Figure S11. ^{1}H NMR spectrum (500 MHz, CD_3OD) of **2** 15
Figure S12. ^{13}C NMR Spectrum (125 MHz, $CDCl_3$) of **2** 15
Figure S13. ^{1}H-^{1}H COSY spectrum of **2** 16
Figure S14. HSQC spectrum of **2** 16
Figure S15. HMBC spectrum of **2** 17
Figure S16. NOESY spectrum of **2** 17
Figure S17. HRESIMS spectrum of **2** 18
Figure S18. UV spectrum of **2** 18
Figure S19. CD spectrum of **2** 19
Figure S20. IR spectrum of **2** 19
Figure S21. ^{1}H NMR spectrum (500 MHz, CD_3OD) of **3** 20
Figure S22. ^{13}C NMR Spectrum (125 MHz, $CDCl_3$) of **3** 20
Figure S23. ^{1}H-^{1}H COSY spectrum of **3** 21
Figure S24. HSQC spectrum of **3** 21
Figure S25. HMBC spectrum of **3** 22
Figure S26. NOESY spectrum of **3** 22
Figure S27. HRESIMS spectrum of **3** 23
Figure S28. UV spectrum of **3** 23
Figure S29. CD spectrum of **3** 24

Figure S30. IR spectrum of **3** 24
Figure S31. ^{1}H NMR spectrum (500 MHz, CD_3OD) of **4** 25
Figure S32. ^{13}C NMR Spectrum (125 MHz, $CDCl_3$) of **4** 25
Figure S33. ^{1}H-^{1}H COSY spectrum of **4** 26
Figure S34. HSQC spectrum of **4** 26
Figure S35. HMBC spectrum of **4** 27
Figure S36. NOESY spectrum of **4** 27
Figure S37. HRESIMS spectrum of **4** 28
Figure S38. UV spectrum of **4** 28
Figure S39. CD spectrum of **4** 29
Figure S40. IR spectrum of **4** 29
Figure S41. ^{1}H NMR spectrum (500 MHz, CD_3OD) of **5** 30
Figure S42. ^{13}C NMR Spectrum (125 MHz, $CDCl_3$) of **5** 30
Figure S43. ^{1}H-^{1}H COSY spectrum of **5** 31
Figure S44. HSQC spectrum of **5** 31
Figure S45. HMBC spectrum of **5** 32
Figure S46. NOESY spectrum of **5** 32
Figure S47. HRESIMS spectrum of **5** 33
Figure S48. UV spectrum of **5** 33
Figure S49. CD spectrum of **5** 34
Figure S50. IR spectrum of **5** 34
Figure S51. ^{1}H NMR spectrum (500 MHz, CD_3OD) of **6** 35
Figure S52. ^{13}C NMR Spectrum (125 MHz, $CDCl_3$) of **6** 35
Figure S53. ^{1}H-^{1}H COSY spectrum of **6** 36
Figure S54. HSQC spectrum of **6** 36
Figure S55. HMBC spectrum of **6** 37
Figure S56. NOESY spectrum of **6** 37
Figure S57. HRESIMS spectrum of **6** 38
Figure S58. UV spectrum of **6** 38
Figure S59. CD spectrum of **6** 39
Figure S60. ^{1}H NMR spectrum (500 MHz, CD_3OD) of **7** 39
Figure S61. ^{13}C NMR Spectrum (125 MHz, $CDCl_3$) of **7** 40

Figure S62. ^{1}H-^{1}H COSY spectrum of **7**......40
Figure S63. HSQC spectrum of **7**......41
Figure S64. HMBC spectrum of **7**......41
Figure S65. NOESY spectrum of **7**......42
Figure S66. HRESIMS spectrum of **7**......42
Figure S67. UV spectrum of **7**......43
Figure S68. CD spectrum of **7**......43
Figure S69. IR spectrum of **7**......44
Figure S70. ^{1}H NMR spectrum (500 MHz, CD_3OD) of **8**......44
Figure S71. ^{13}C NMR Spectrum (125 MHz, $CDCl_3$) of **8**......45
Figure S72. ^{1}H-^{1}H COSY spectrum of **8**......45
Figure S73. HSQC spectrum of **8**......46
Figure S74. HMBC spectrum of **8**......46
Figure S75. NOESY spectrum of **8**......47
Figure S76. HRESIMS spectrum of **8**......47
Figure S77. UV spectrum of **8**......48
Figure S78. CD spectrum of **8**......48
Figure S79. IR spectrum of **8**......49
Figure S80. ^{1}H NMR spectrum (500 MHz, CD_3OD) of **9**......49
Figure S81. ^{13}C NMR Spectrum (125 MHz, $CDCl_3$) of **9**......50
Figure S82. ^{1}H-^{1}H COSY spectrum of **9**......50
Figure S83. HSQC spectrum of **9**......51
Figure S84. HMBC spectrum of **9**......51
Figure S85. HRESIMS spectrum of **9**......52
Figure S86. UV spectrum of **9**......52
Figure S87. CD spectrum of **9**......53
Figure S88. ^{1}H NMR spectrum (500 MHz, CD_3OD) of **10**......53
Figure S89. ^{13}C NMR Spectrum (125 MHz, $CDCl_3$) of **10**......54
Figure S90. ^{1}H-^{1}H COSY spectrum of **10**......54
Figure S91. HSQC spectrum of **10**......55
Figure S92. HMBC spectrum of **10**......55
Figure S93. NOESY spectrum of **10**......56

Figure S94. HRESIMS spectrum of **10**..56
Figure S95. UV spectrum of **10** 57
Figure S96. CD spectrum of **10** 57
Figure S97. IR spectrum of **10**...58
Figure S98. ^{1}H-^{1}H COSY spectrum of **11**...58
Figure S99. HSQC spectrum of **11** 59
Figure S100. HMBC spectrum of **11** 59
Figure S101. NOESY spectrum of **11**..60
Figure S102. HRESIMS spectrum of **11**..60
Figure S103. UV spectrum of **11** 61
Figure S104. CD spectrum of **11** 61
Figure S105. IR spectrum of **11**..62

1. X-ray crytallographic data of compounds 1, **2**, **5**, and **9.**

The single-crystal X-ray diffraction data were collected at 100K for 1 on Agilent Xcalibur Nova single-crystal diffractometer using CuKα radiation. The crystal structure was refined by full-matrix least-squares calculation. Hydrogen atoms bonded to carbons were located by the geometrically ideal positions by the “ride on” method. Hydrogen atoms bonded to oxygen were placed on the difference Fourier method and were included in the calculation of structure factors S3 S4 with isotropic temperature factors. Crystallographic data for **1**, **2**, **5**, and **9** reported in this paper have been deposited in the Cambridge Crystallographic Data Centre. (Deposition number: CCDC 2008519 for **1**, CCDC 2008520 for **2**, CCDC 2047671 for **5**, and CCDC 2047672 for **9**). Copies of these data can be obtained free of charge via www.ccdc.cam.au.ck/conts/retrieving.html.

Table 1 Crystal data and structure refinement for 1.

Identification code	LXX-73_collect
Empirical formula	$C_{12}H_{18}O_4$
Formula weight	226.26
Temperature/K	105.9(6)
Crystal system	orthorhombic
Space group	$P2_12_12$
a/Å	18.34142(11)
b/Å	14.95605(9)
c/Å	4.78701(3)
α/°	90
β/°	90
γ/°	90
Volume/Å^3	1313.150(13)
Z	4
ρ_{calc}g/cm^3	1.281
μ/mm^{-1}	0.842
F(000)	548.0
Crystal size/mm^3	0.4 × 0.3 × 0.3
Radiation	CuKα (λ = 1.54184)
2Θ range for data collection/°	7.628 to 148.912
Index ranges	-22 ≤ h ≤ 22, -18 ≤ k ≤ 17, -5 ≤ l ≤ 5

Reflections collected	15461
Independent reflections	2638 [R_{int} = 0.0213, R_{sigma} = 0.0098]
Data/restraints/parameters	2638/0/173
Goodness-of-fit on F^2	1.042
Final R indexes [I>=2σ (I)]	R_1 = 0.0294, wR_2 = 0.0767
Final R indexes [all data]	R_1 = 0.0296, wR_2 = 0.0769
Largest diff. peak/hole / e $Å^{-3}$	0.28/-0.17
Flack parameter	0.02(5)

Table 2 Crystal data and structure refinement for 2.

Identification code	lxx-75_collect_tw
Empirical formula	$C_{12}H_{18}O_4$
Formula weight	226.26
Temperature/K	293(2)
Crystal system	monoclinic
Space group	$P2_1$
a/Å	5.5941(3)
b/Å	10.2949(7)
c/Å	10.2393(6)
α/°	90
β/°	94.955(5)
γ/°	90
Volume/$Å^3$	587.48(6)
Z	2
ρ_{calc}g/cm^3	1.279
μ/mm^{-1}	0.785
F(000)	244.0
Crystal size/mm^3	0.2 × 0.02 × 0.01
Radiation	CuKα (λ = 1.54184)
2Θ range for data collection/°	8.668 to 148.246
Index ranges	-6 ≤ h ≤ 6, -11 ≤ k ≤ 12, -9 ≤ l ≤ 12
Reflections collected	2073
Independent reflections	2073 [R_{int} = ?, R_{sigma} = 0.0313]
Data/restraints/parameters	2073/1/151
Goodness-of-fit on F^2	1.093
Final R indexes [I>=2σ (I)]	R_1 = 0.0760, wR_2 = 0.2407
Final R indexes [all data]	R_1 = 0.0772, wR_2 = 0.2416

Largest diff. peak/hole / e Å^{-3}	0.60/-0.38
Flack parameter	-0.1(4)

Table 3 Crystal data and structure refinement for 5.

Identification code	luxiuxiang_78_collect2
Empirical formula	$C_{12}H_{18}O_3$
Formula weight	210.26
Temperature/K	100.01(10)
Crystal system	orthorhombic
Space group	$P2_12_12_1$
a/Å	4.74020(10)
b/Å	10.1082(2)
c/Å	23.7681(4)
α/°	90
β/°	90
γ/°	90
Volume/Å^3	1138.85(4)
Z	4
ρ_{calc}g/cm^3	1.226
μ/mm^{-1}	0.703
F(000)	456.0
Crystal size/mm^3	0.2 × 0.06 × 0.04
Radiation	CuKα (λ = 1.54184)
2Θ range for data collection/°	7.438 to 148.23
Index ranges	$-3 \le h \le 5$, $-10 \le k \le 12$, $-29 \le l \le 29$
Reflections collected	6658
Independent reflections	2257 [R_{int} = 0.0333, R_{sigma} = 0.0294]
Data/restraints/parameters	2257/0/140
Goodness-of-fit on F^2	1.058
Final R indexes [I>=2σ (I)]	R_1 = 0.0341, wR_2 = 0.0869
Final R indexes [all data]	R_1 = 0.0351, wR_2 = 0.0875
Largest diff. peak/hole / e Å^{-3}	0.15/-0.17
Flack parameter	0.04(9)

Table 4 Crystal data and structure refinement for 9.

Identification code	lxx-9_collect
Empirical formula	$C_{12}H_{14}O_3$
Formula weight	206.23
Temperature/K	100.01(10)
Crystal system	monoclinic
Space group	$P2_1/c$
a/Å	11.1945(3)
b/Å	13.5226(3)
c/Å	7.14770(10)
α/°	90
β/°	91.387(2)
γ/°	90
Volume/Å^3	1081.69(4)
Z	4
ρ_{calc}g/cm^3	1.266
μ/mm^{-1}	0.740
F(000)	440.0
Crystal size/mm^3	0.5 × 0.1 × 0.05
Radiation	CuKα (λ = 1.54184)
2Θ range for data collection/°	7.9 to 147.988
Index ranges	$-10 \le h \le 13$, $-12 \le k \le 16$, $-8 \le l \le 8$
Reflections collected	5484
Independent reflections	2148 [R_{int} = 0.0297, R_{sigma} = 0.0396]
Data/restraints/parameters	2148/0/140
Goodness-of-fit on F^2	1.063
Final R indexes [I>=2σ (I)]	R_1 = 0.0589, wR_2 = 0.1641
Final R indexes [all data]	R_1 = 0.0658, wR_2 = 0.1711
Largest diff. peak/hole / e Å^{-3}	0.61/-0.33

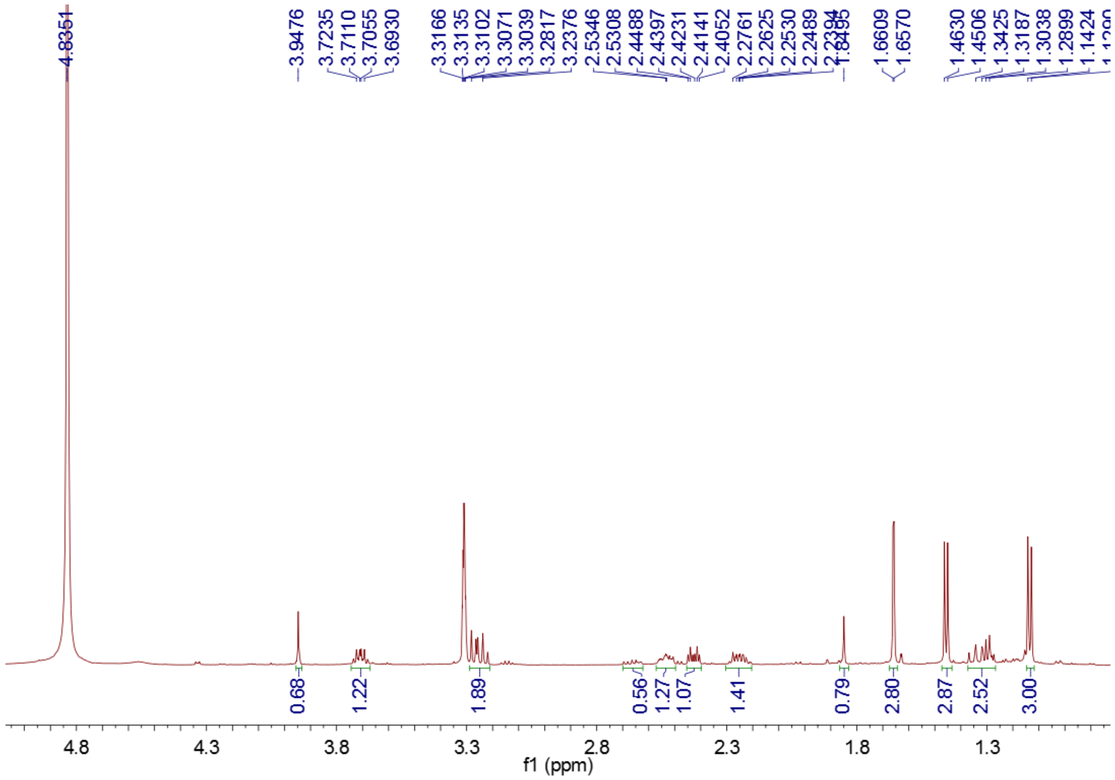

Figure S1. ^{1}H NMR spectrum (500 MHz, CD_3OD) of **1**.

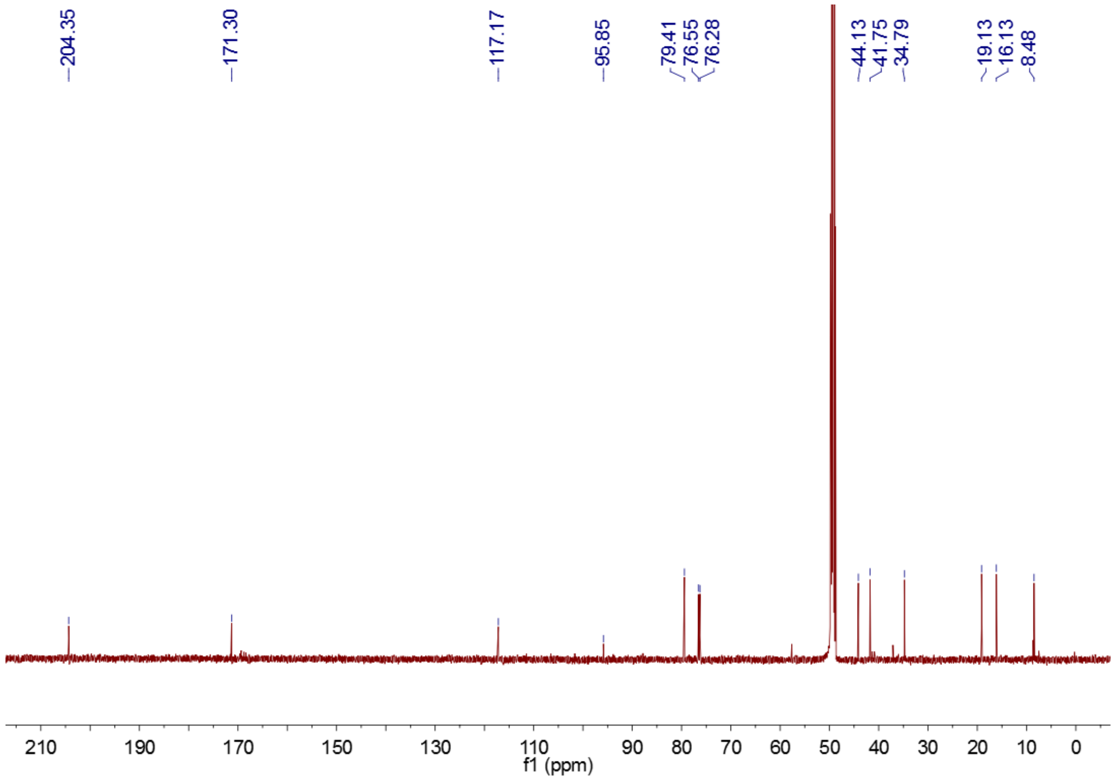

Figure S2. ^{13}C NMR spectrum (125 MHz, CD_3OD) of **1**.

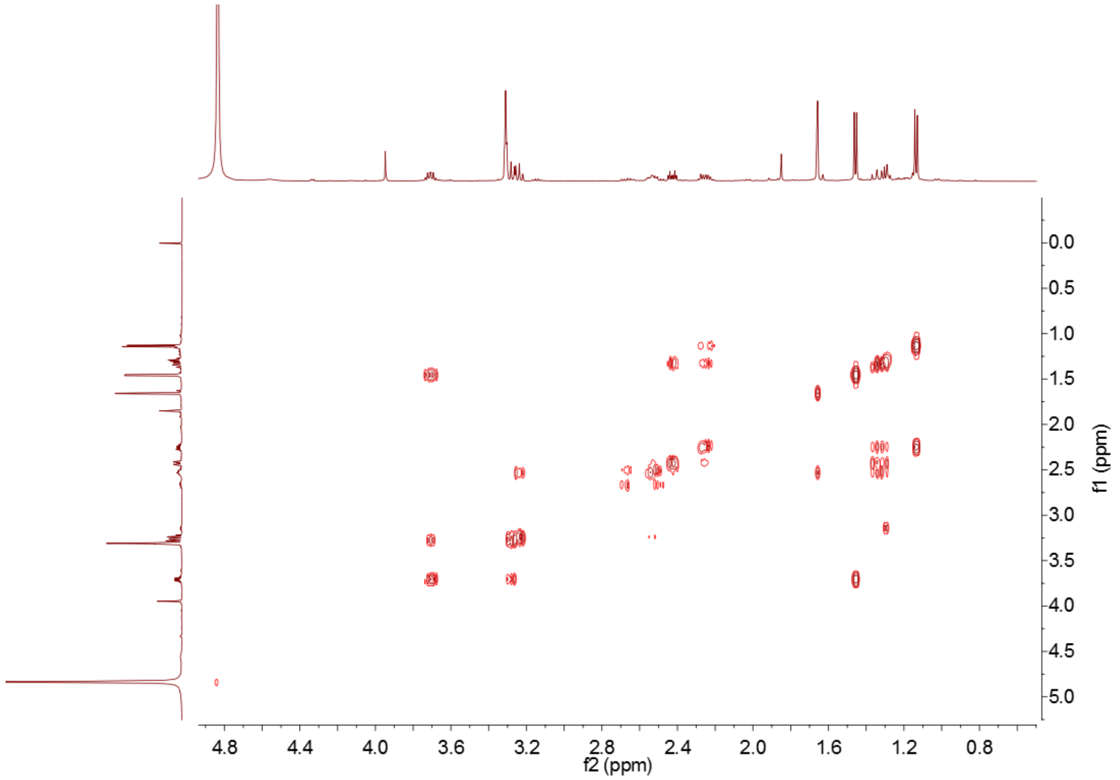

Figure S3. ^{1}H-^{1}H COSY spectrum of **1**.

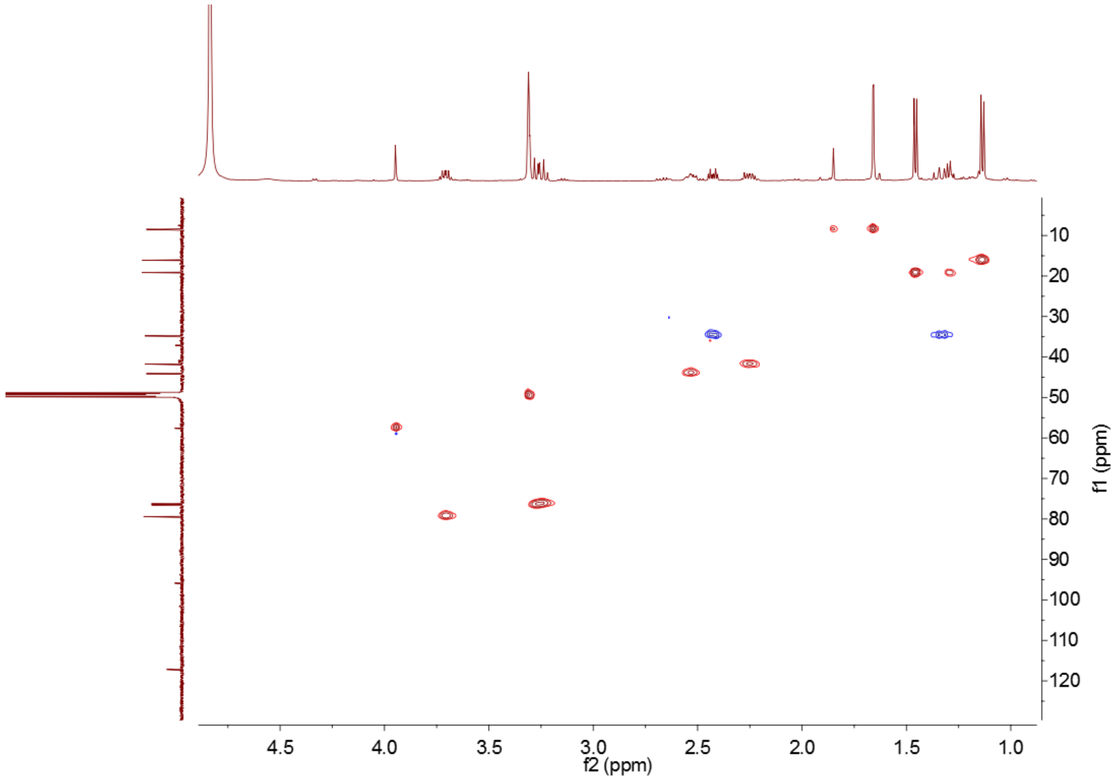

Figure S4. HSQC spectrum of **1**.

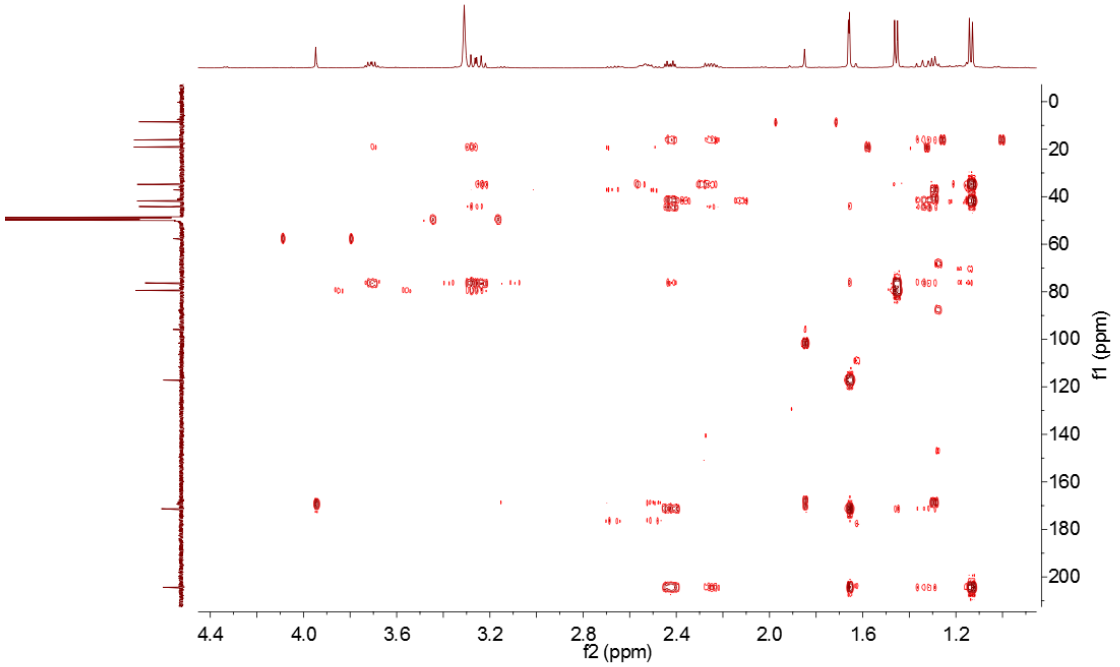

Figure S5. HMBC spectrum of **1**.

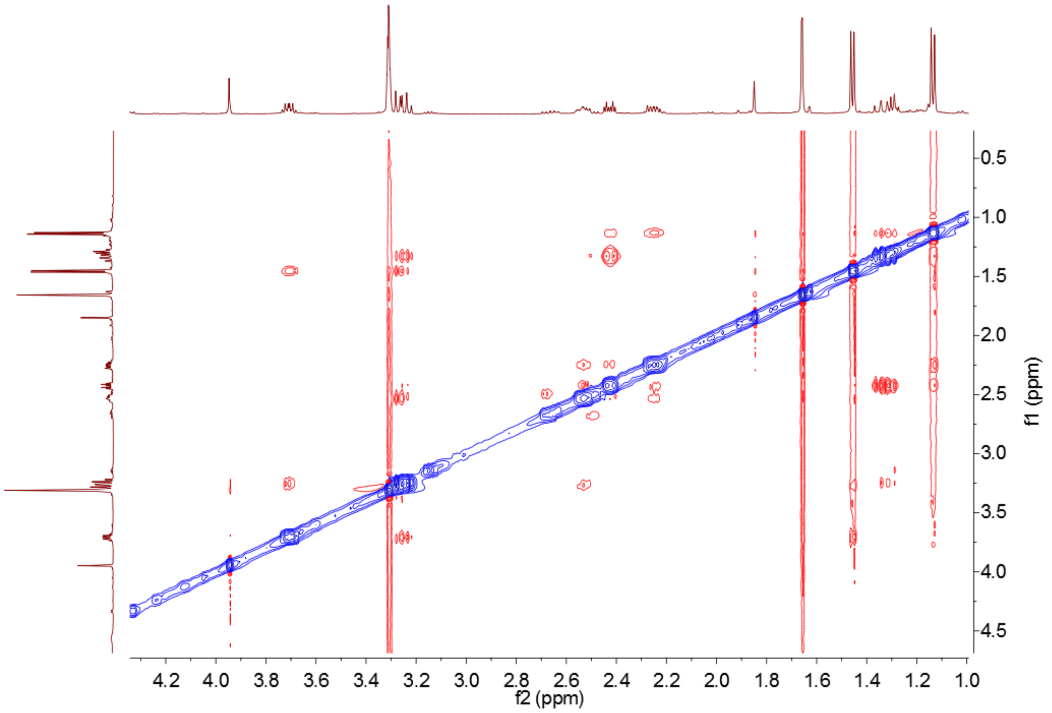

Figure S6. NOESY spectrum of **1**.

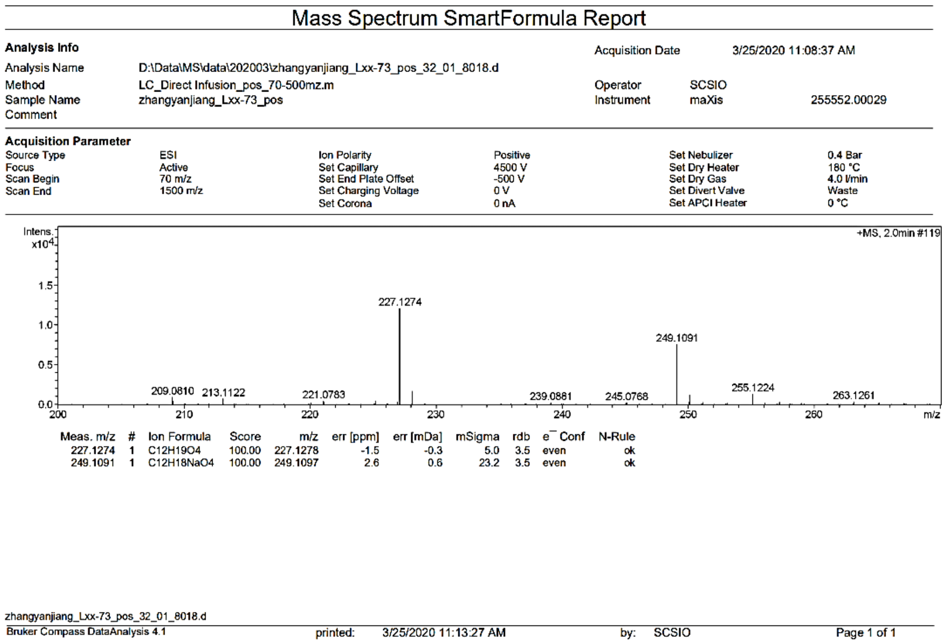

Mass Spectrum SmartFormula Report

Analysis Info

Analysis Name	D:\Data\MS\data\202003\zhangyanjiang_Lxx-73_pos_32_01_8018.d	Acquisition Date	3/25/2020 11:08:37 AM
Method	LC_Direct Infusion_pos_70-500mz.m	Operator	SCSIO
Sample Name	zhangyanjiang_Lxx-73_pos	Instrument	maXis 255552.00029
Comment			

Acquisition Parameter

Source Type	ESI	Ion Polarity	Positive	Set Nebulizer	0.4 Bar
Focus	Active	Set Capillary	4500 V	Set Dry Heater	180 °C
Scan Begin	70 m/z	Set End Plate Offset	-500 V	Set Dry Gas	4.0 l/min
Scan End	1500 m/z	Set Charging Voltage	0 V	Set Divert Valve	Waste
		Set Corona	0 nA	Set APCI Heater	0 °C

Meas. m/z	#	Ion Formula	Score	m/z	err [ppm]	err [mDa]	mSigma	rdb	e⁻ Conf	N-Rule
227.1274	1	C12H19O4	100.00	227.1278	-1.5	-0.3	5.0	3.5	even	ok
249.1091	1	C12H18NaO4	100.00	249.1097	2.6	0.6	23.2	3.5	even	ok

zhangyanjiang_Lxx-73_pos_32_01_8018.d
Bruker Compass DataAnalysis 4.1 printed: 3/25/2020 11:13:27 AM by: SCSIO Page 1 of 1

Figure S7. HRESIMS spectrum of **1**.

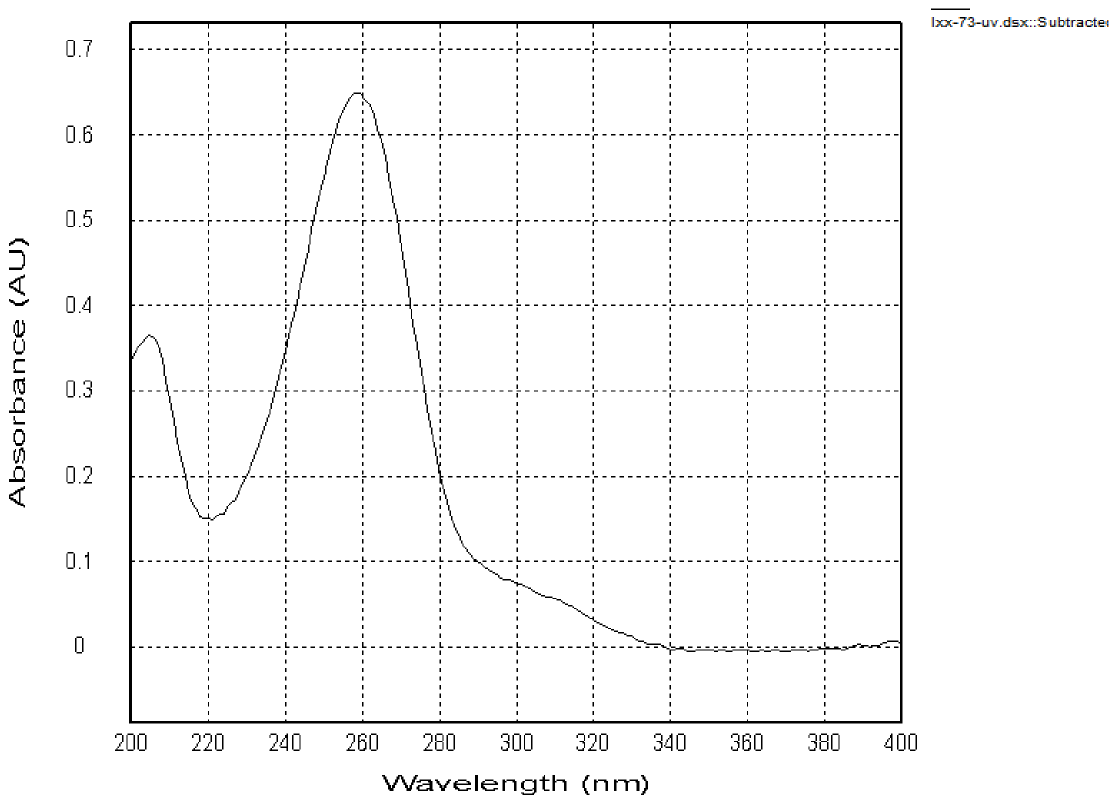

Figure S8. UV spectrum of **1**.

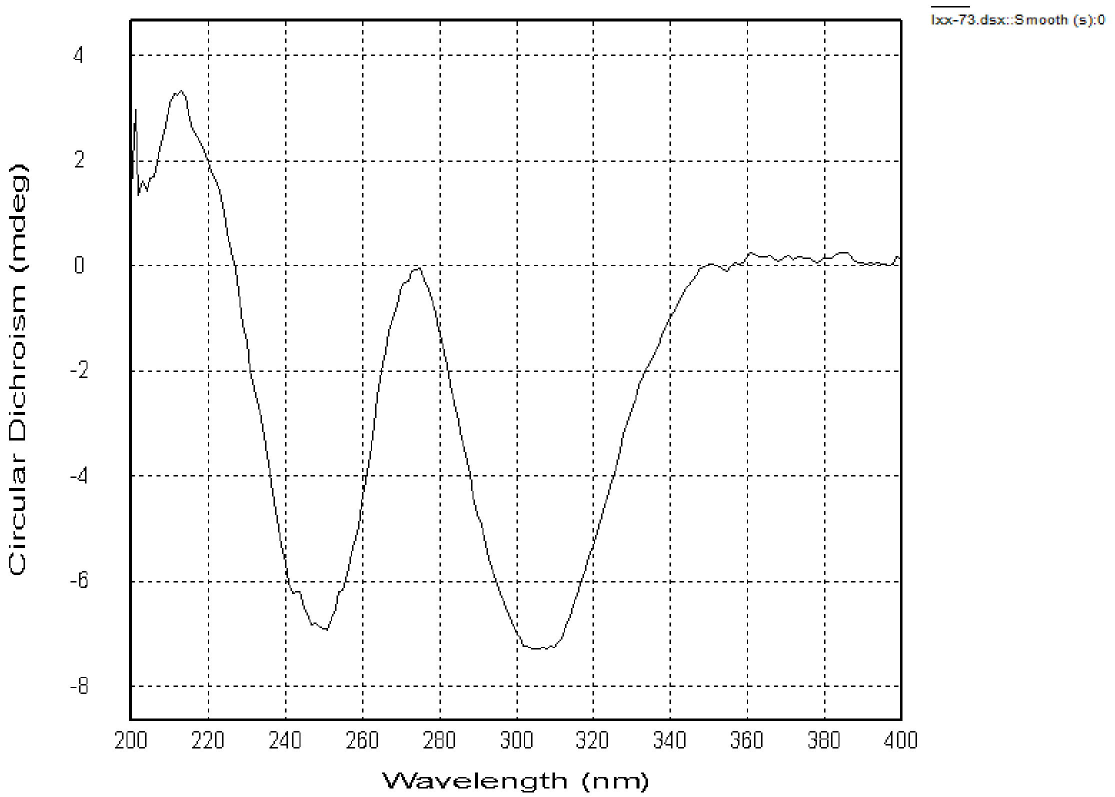

Figure S9. CD spectrum of **1**.

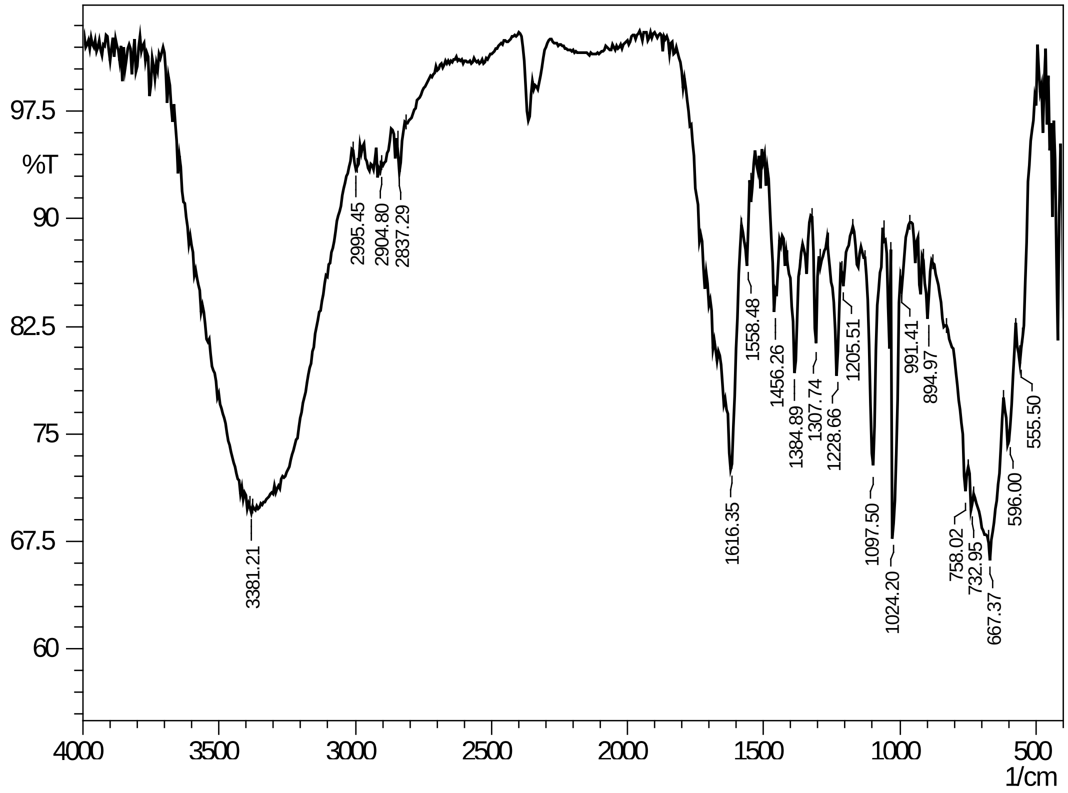

Figure S10. IR spectrum of **1**.

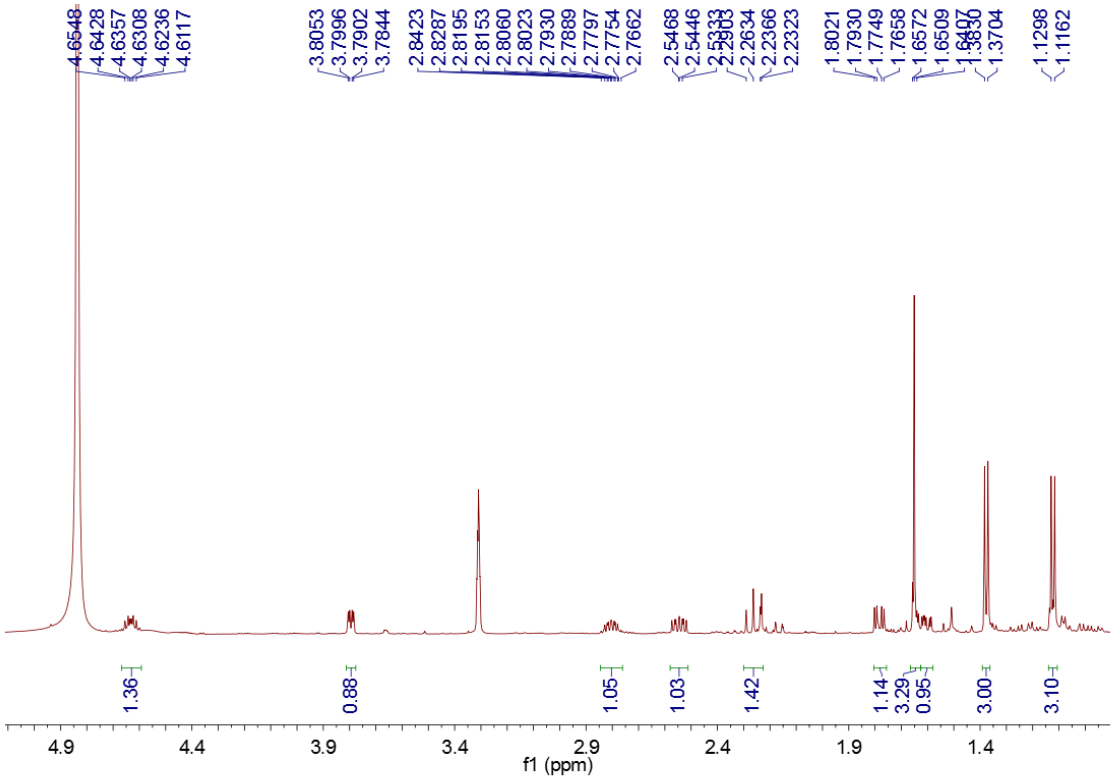

Figure S11. ^{1}H NMR spectrum (500 MHz, CD_3OD) of **2**.

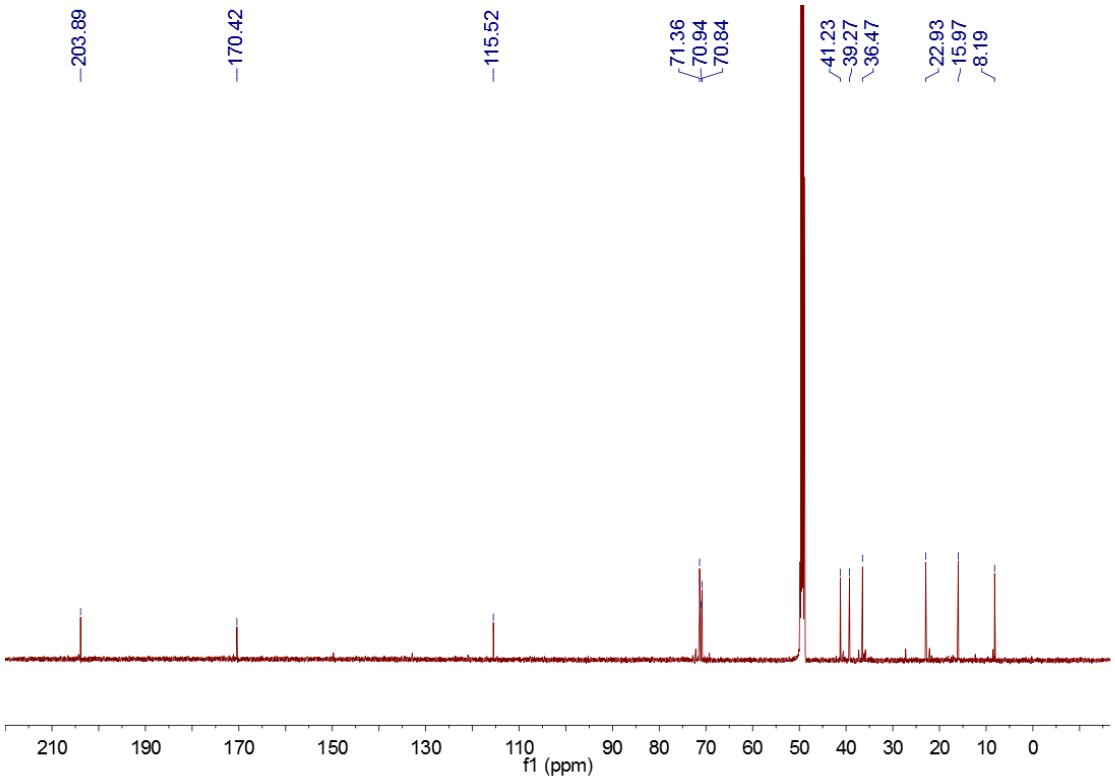

Figure S12. ^{13}C NMR spectrum (125 MHz, CD_3OD) of **2**.

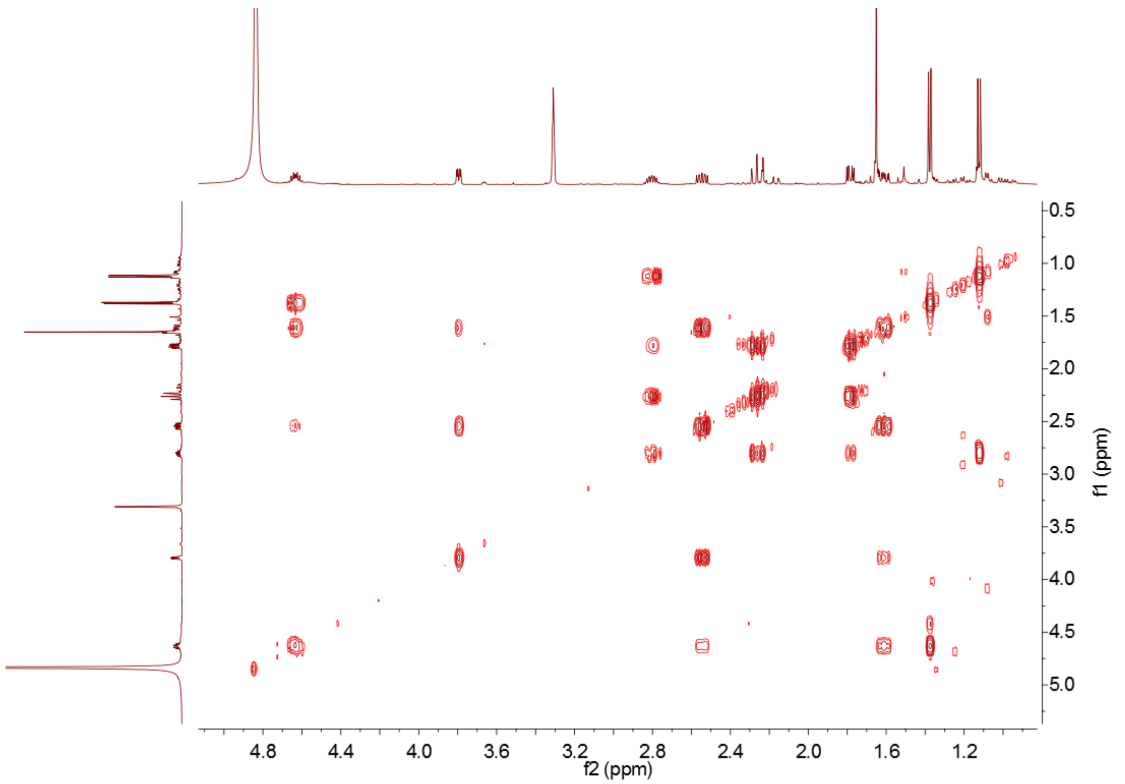

Figure S13. ^{1}H-^{1}H COSY spectrum of **2**.

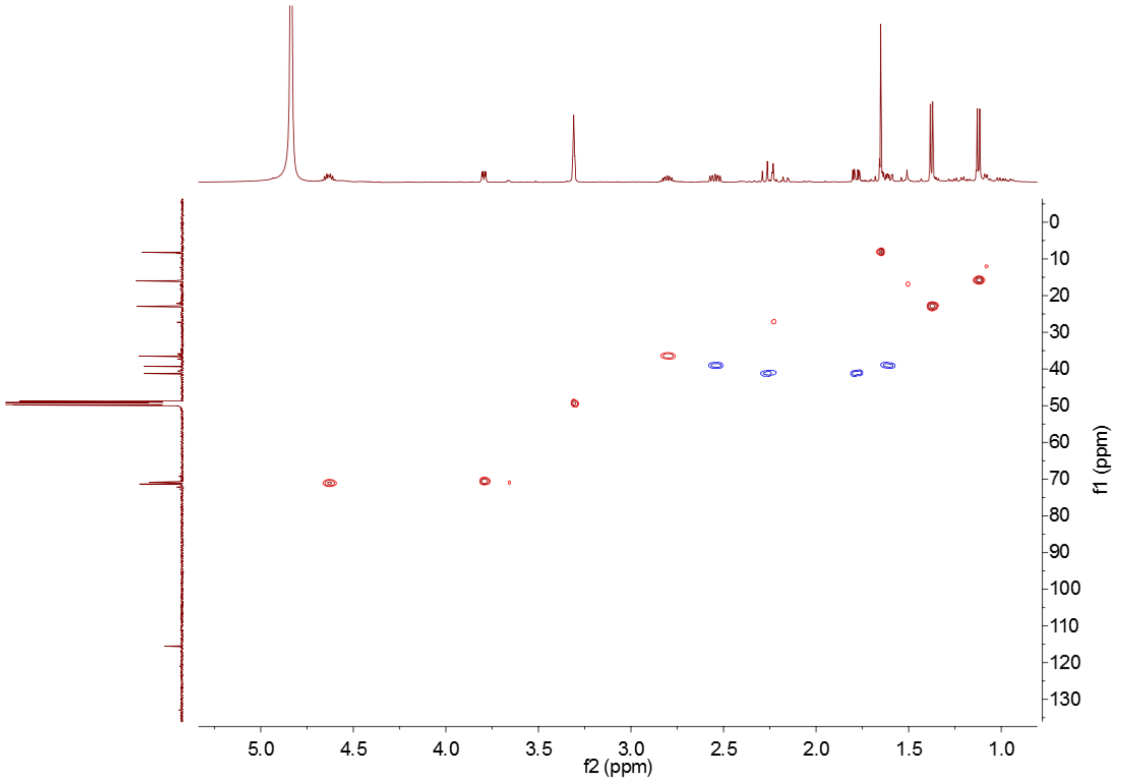

Figure S14. HSQC spectrum of **2**.

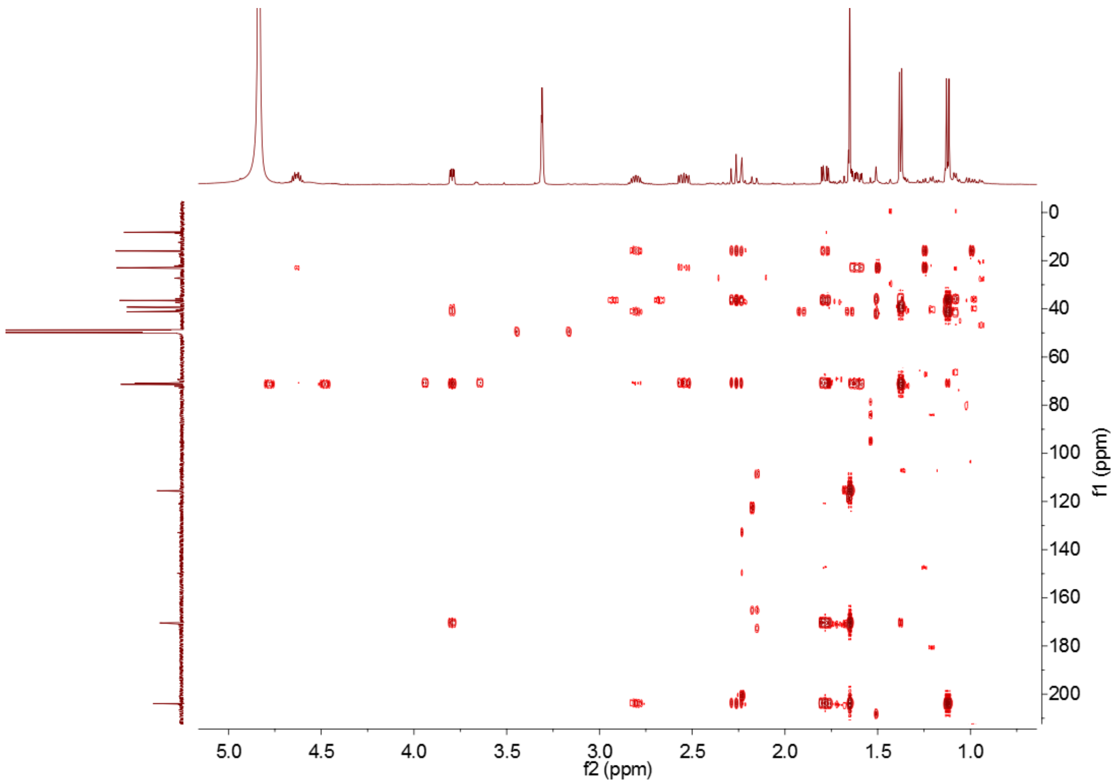

Figure S15. HMBC spectrum of **2**.

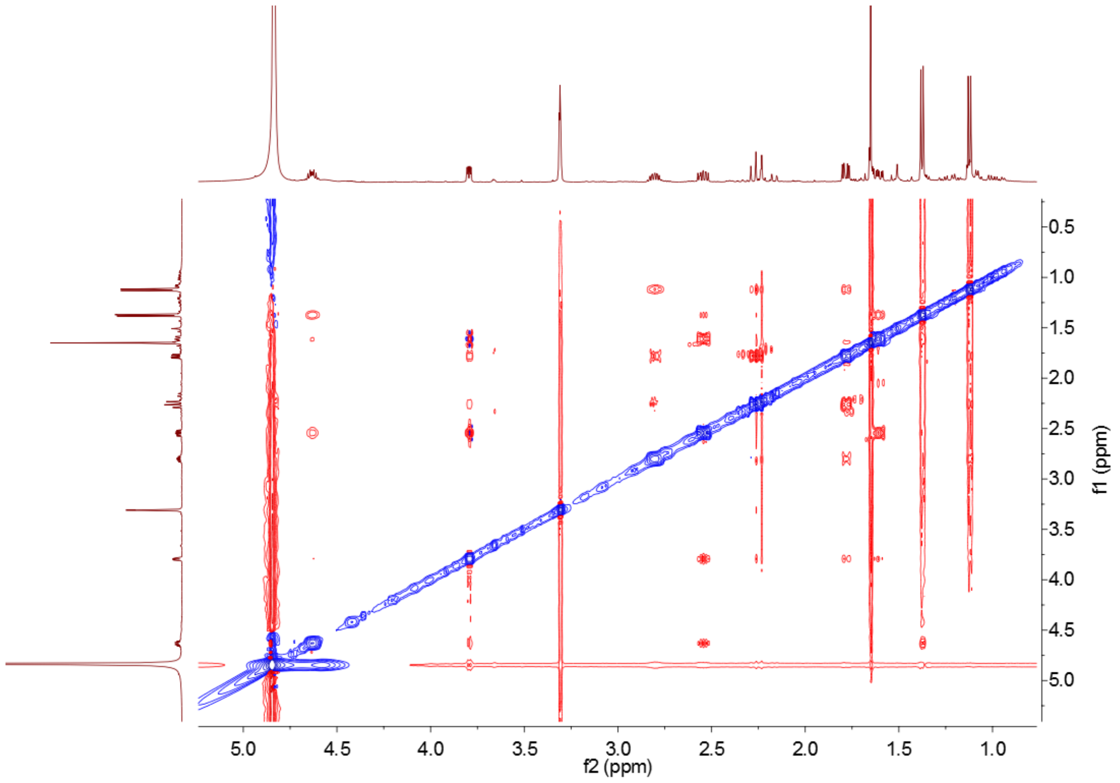

Figure S16. NOESY spectrum of **2**.

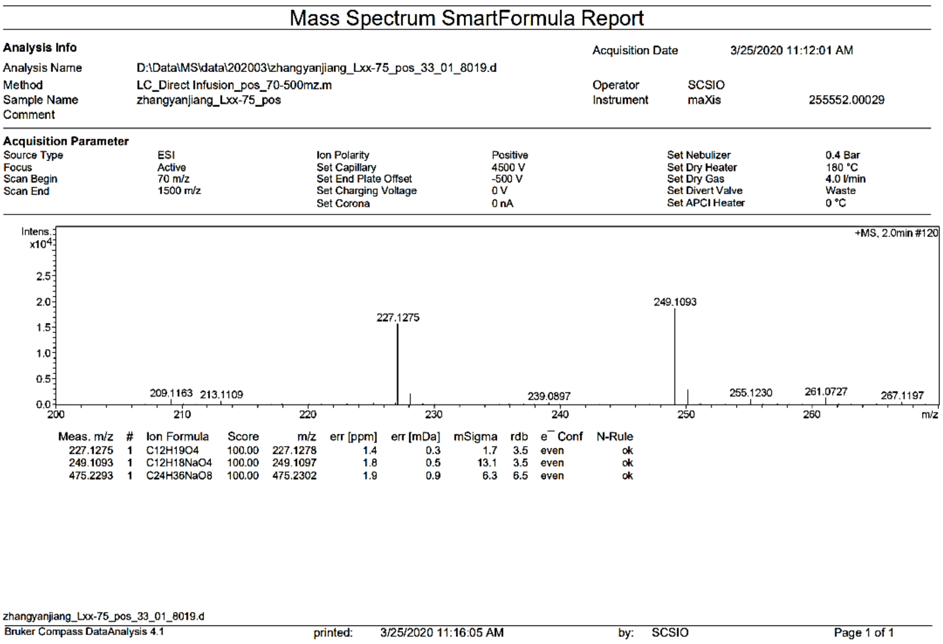

Mass Spectrum SmartFormula Report

Analysis Info

Analysis Name	D:\Data\MS\data\202003\zhangyanjiang_Lxx-75_pos_33_01_8019.d	Acquisition Date	3/25/2020 11:12:01 AM	
Method	LC_Direct Infusion_pos_70-500mz.m	Operator	SCSIO	
Sample Name	zhangyanjiang_Lxx-75_pos	Instrument	maXis	255552.00029
Comment				

Acquisition Parameter

Source Type	ESI	Ion Polarity	Positive	Set Nebulizer	0.4 Bar
Focus	Active	Set Capillary	4500 V	Set Dry Heater	180 °C
Scan Begin	70 m/z	Set End Plate Offset	-500 V	Set Dry Gas	4.0 l/min
Scan End	1500 m/z	Set Charging Voltage	0 V	Set Divert Valve	Waste
		Set Corona	0 nA	Set APCI Heater	0 °C

Meas. m/z	#	Ion Formula	Score	m/z	err [ppm]	err [mDa]	mSigma	rdb	e⁻ Conf	N-Rule
227.1275	1	C12H19O4	100.00	227.1278	1.4	0.3	1.7	3.5	even	ok
249.1093	1	C12H18NaO4	100.00	249.1097	1.8	0.5	13.1	3.5	even	ok
475.2293	1	C24H36NaO8	100.00	475.2302	1.9	0.9	6.3	6.5	even	ok

zhangyanjiang_Lxx-75_pos_33_01_8019.d
Bruker Compass DataAnalysis 4.1 printed: 3/25/2020 11:16:05 AM by: SCSIO Page 1 of 1

Figure S17. HRESIMS spectrum of **2**.

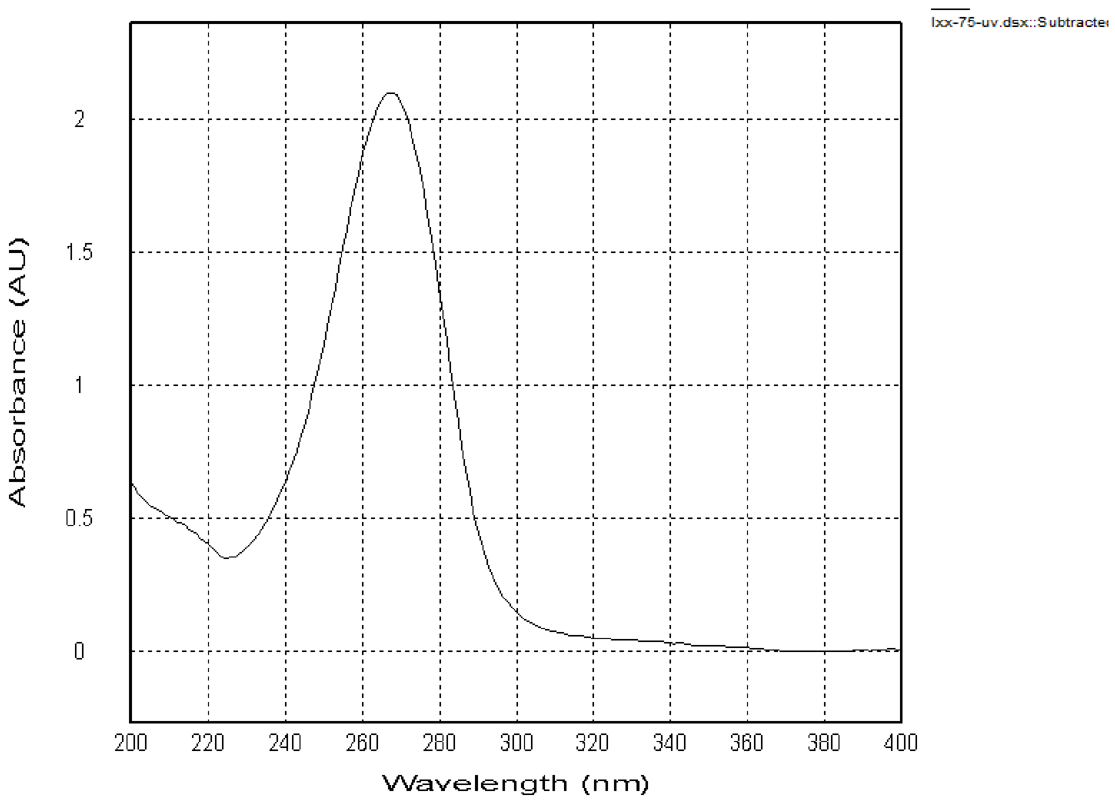

Figure S18. UV spectrum of **2**.

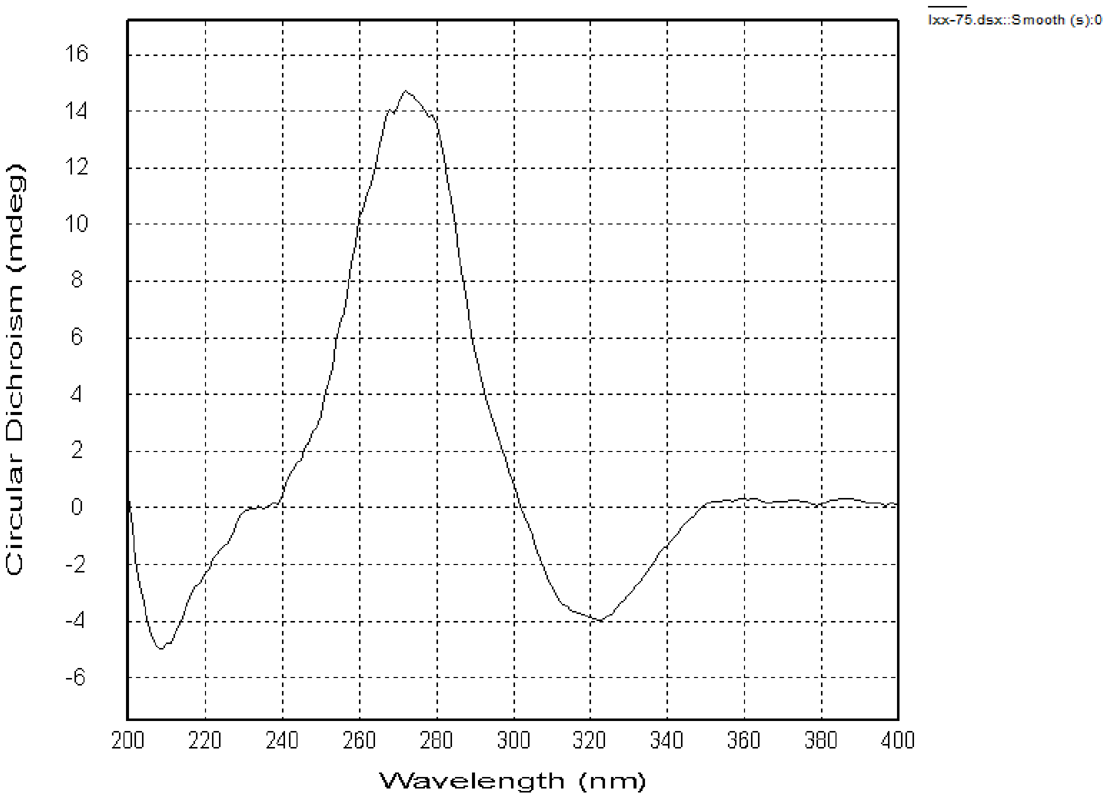

Figure S19. CD spectrum of **2**.

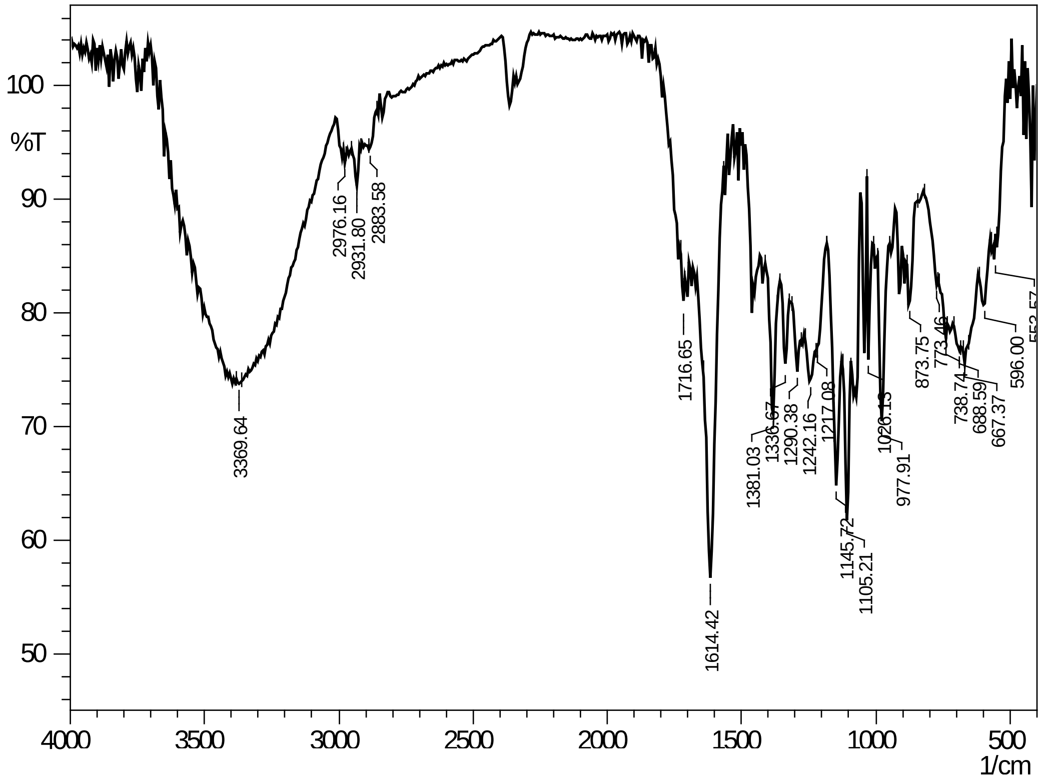

Figure S20. IR spectrum of **2**.

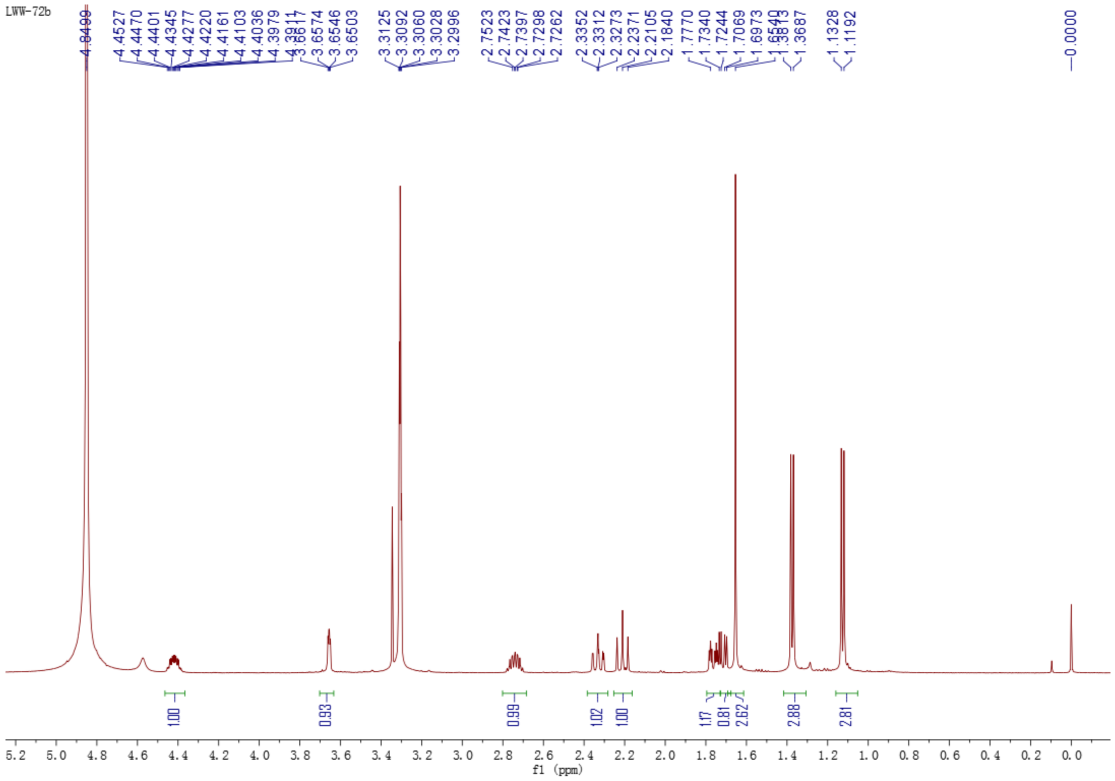

Figure S21. ^{1}H NMR spectrum (500 MHz, CD_3OD) of **3**.

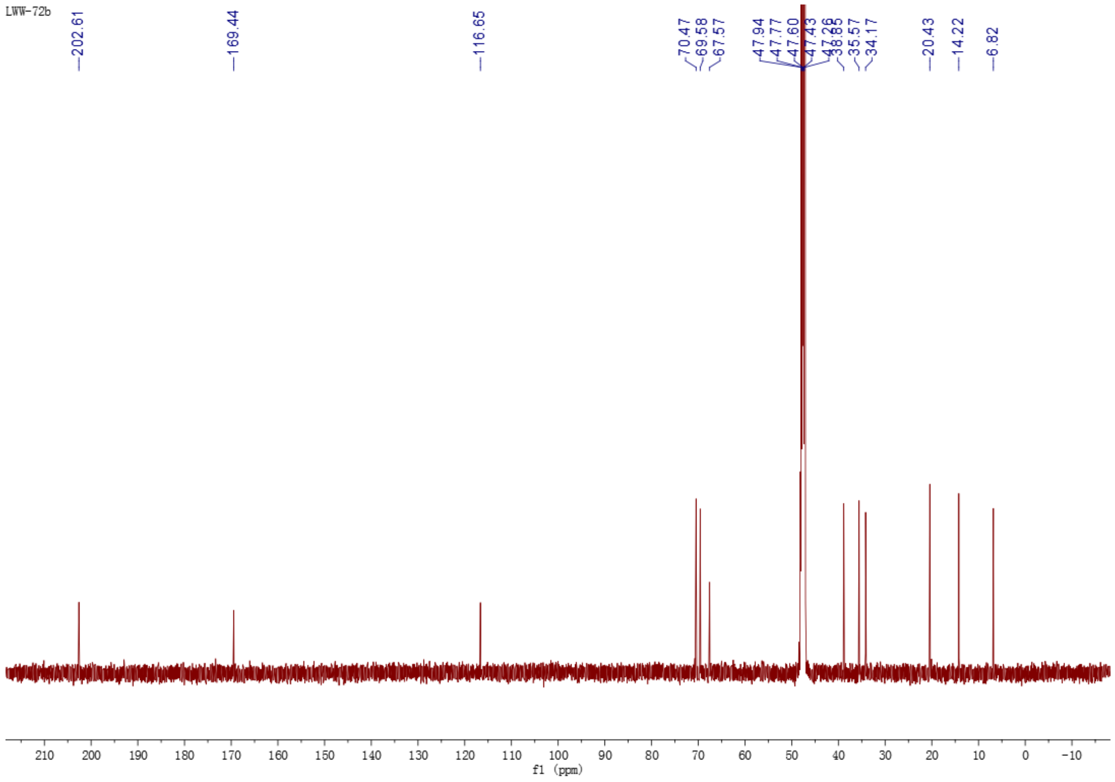

Figure S22. ^{13}C NMR spectrum (125 MHz, CD_3OD) of **3**.

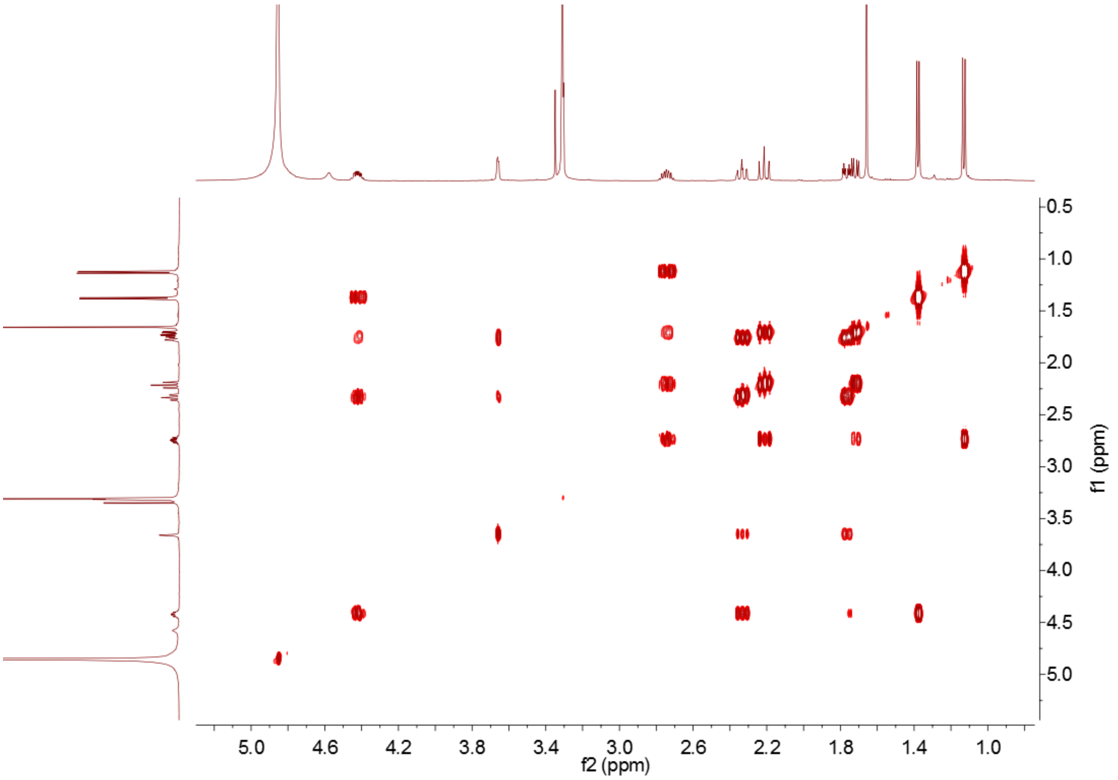

Figure S23. ^{1}H-^{1}H COSY spectrum of **3**.

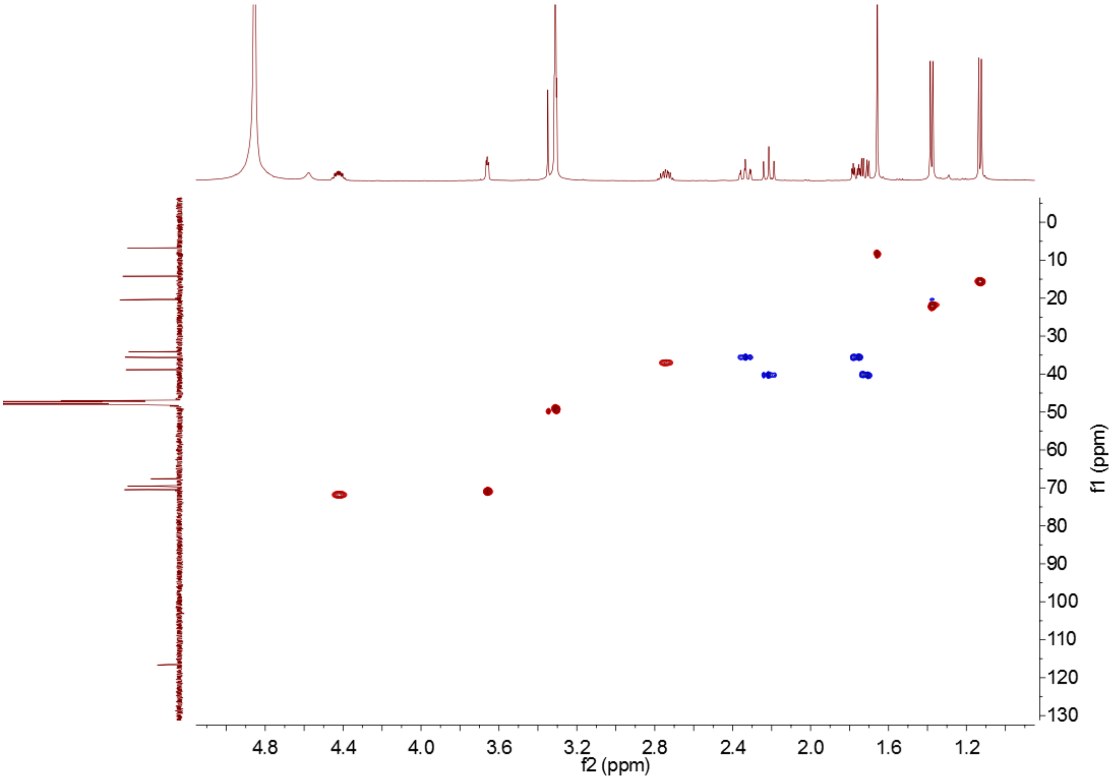

Figure S24. HSQC spectrum of **3**.

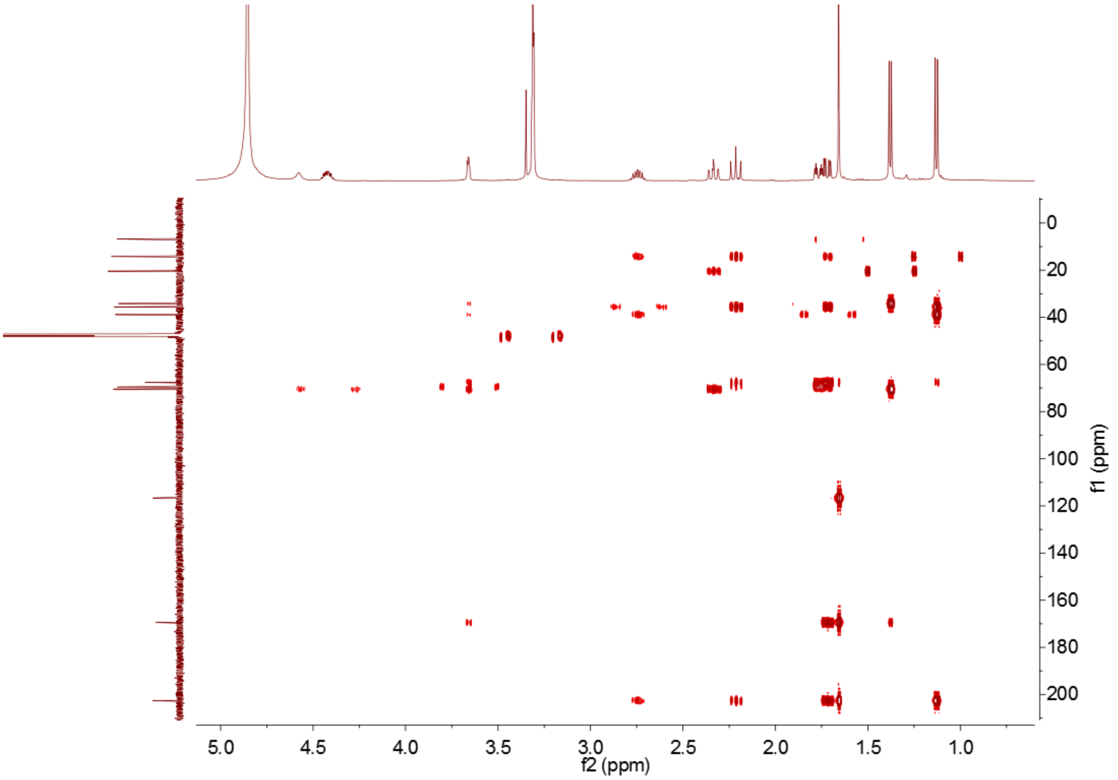

Figure S25. HMBC spectrum of **3**.

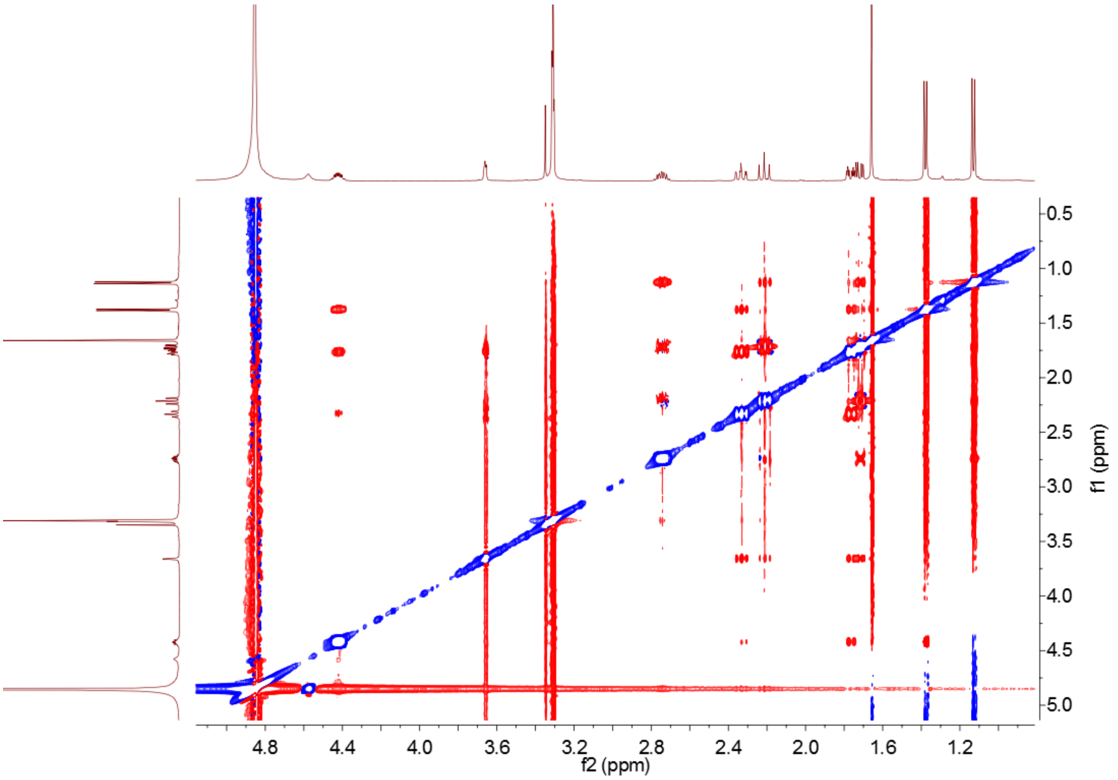

Figure S26. NOESY spectrum of **3**.

Mass Spectrum SmartFormula Report

Analysis Info

Analysis Name	D:\Data\MS\data\202011\luxiuxiang_LWW-72b_pos_1_01_9191.d	Acquisition Date	11/10/2020 10:58:00 AM
Method	LC_Direct Infusion_pos_70-500mz.m	Operator	SCSIO
Sample Name	luxiuxiang_LWW-72b_pos	Instrument	maXis 255552.00029
Comment			

Acquisition Parameter

Source Type	ESI	Ion Polarity	Positive	Set Nebulizer	0.4 Bar
Focus	Active	Set Capillary	4500 V	Set Dry Heater	180 °C
Scan Begin	70 m/z	Set End Plate Offset	-500 V	Set Dry Gas	4.0 l/min
Scan End	1500 m/z	Set Charging Voltage	0 V	Set Divert Valve	Waste
		Set Corona	0 nA	Set APCI Heater	0 °C

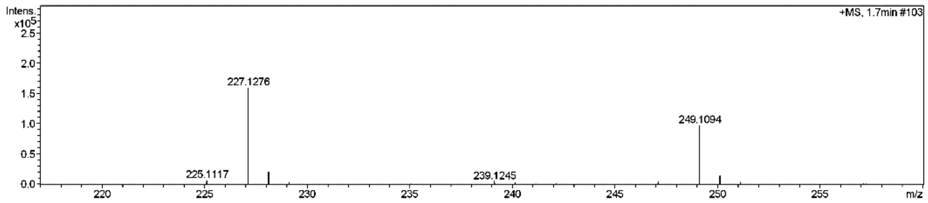

Meas. m/z	#	Ion Formula	Score	m/z	err [ppm]	err [mDa]	mSigma	rdb	e⁻ Conf	N-Rule
227.1276	1	C12H19O4	100.00	227.1278	0.8	0.2	2.4	3.5	even	ok
249.1094	1	C12H18NaO4	100.00	249.1097	1.4	0.4	8.1	3.5	even	ok
475.2286	1	C24H36NaO8	100.00	475.2302	-3.5	-1.7	5.8	6.5	even	ok

luxiuxiang_LWW-72b_pos_1_01_9191.d

Bruker Compass DataAnalysis 4.1 printed: 11/10/2020 11:02:54 AM by: SCSIO Page 1 of 1

Figure S27. HRESIMS spectrum of **3**.

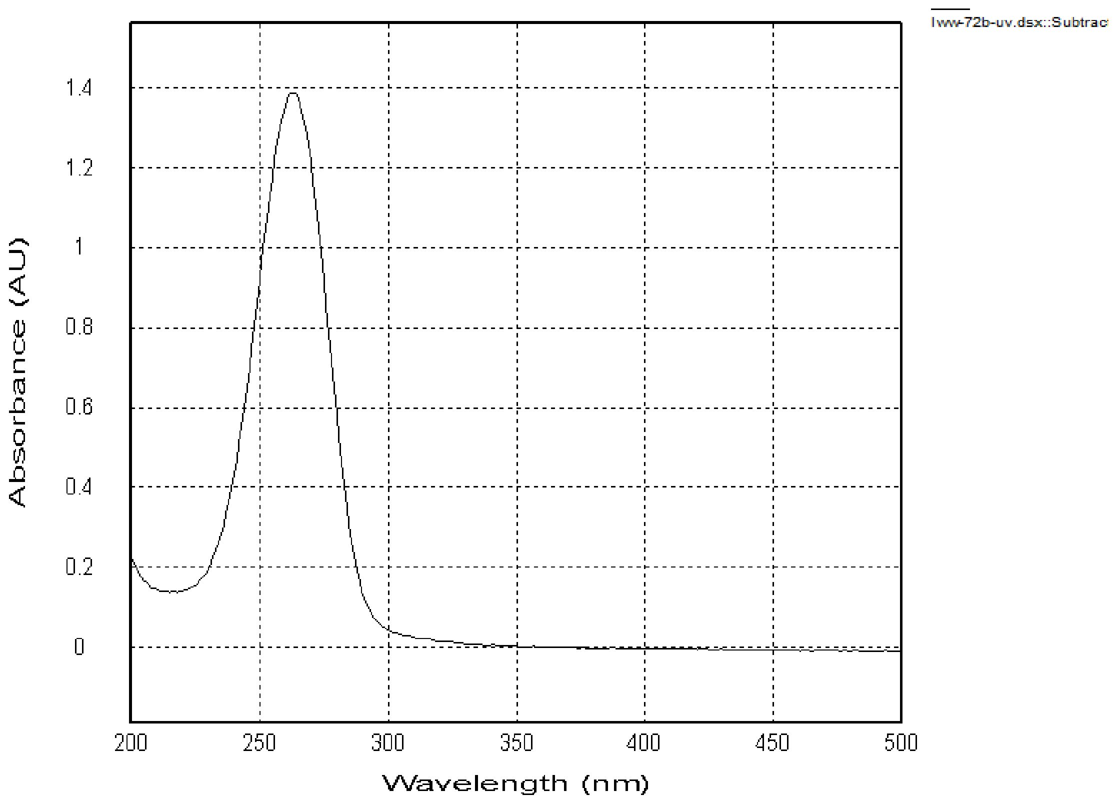

Figure S28. UV spectrum of **3**.

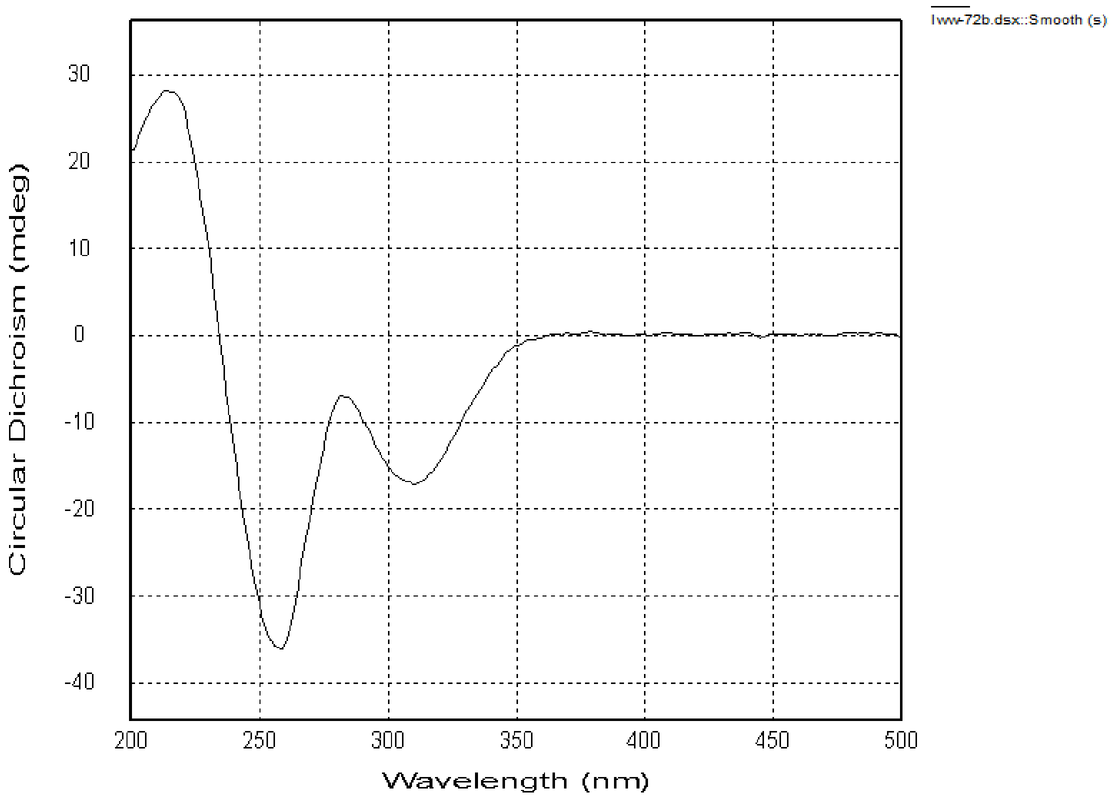

Figure S29. CD spectrum of **3**.

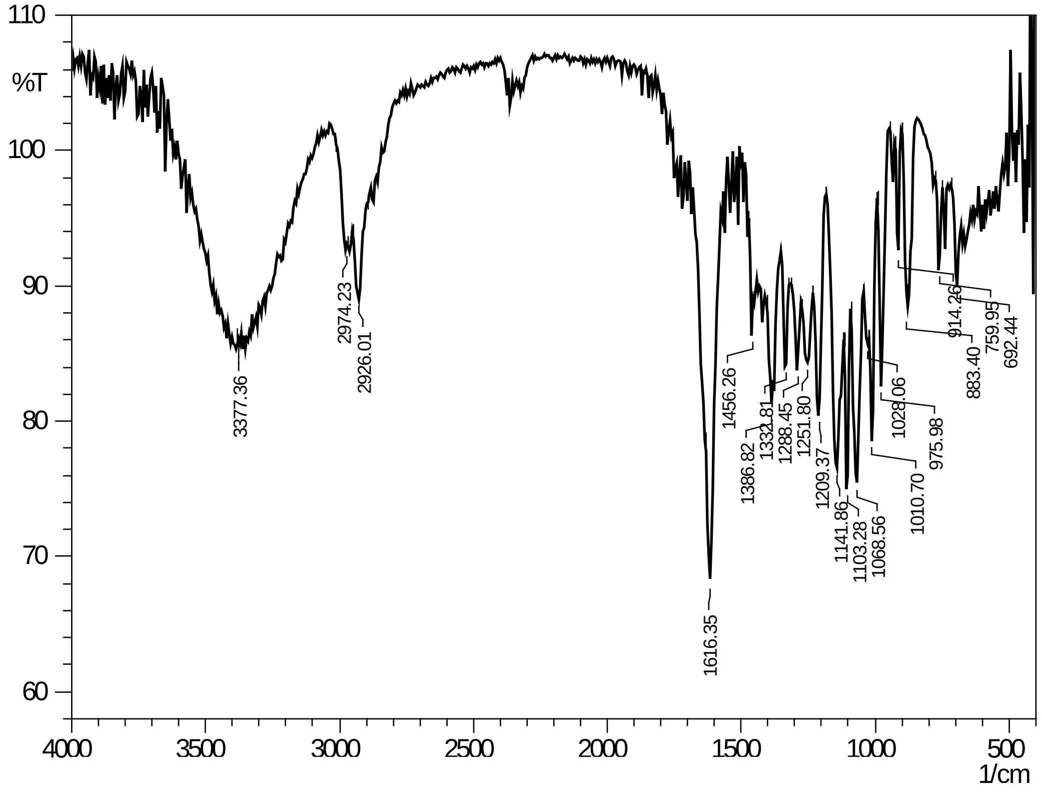

Figure S30. IR spectrum of **3**.

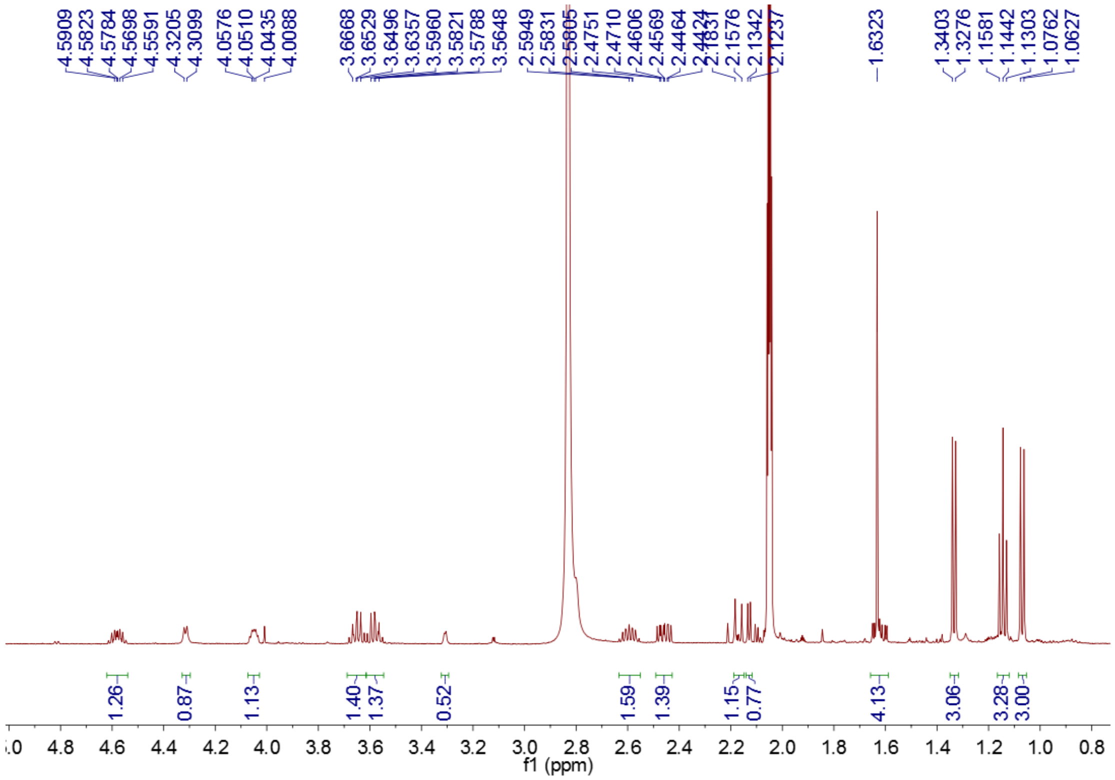

Figure S31. ^{1}H NMR spectrum (500 MHz, CD_3COCD_3) of **4**.

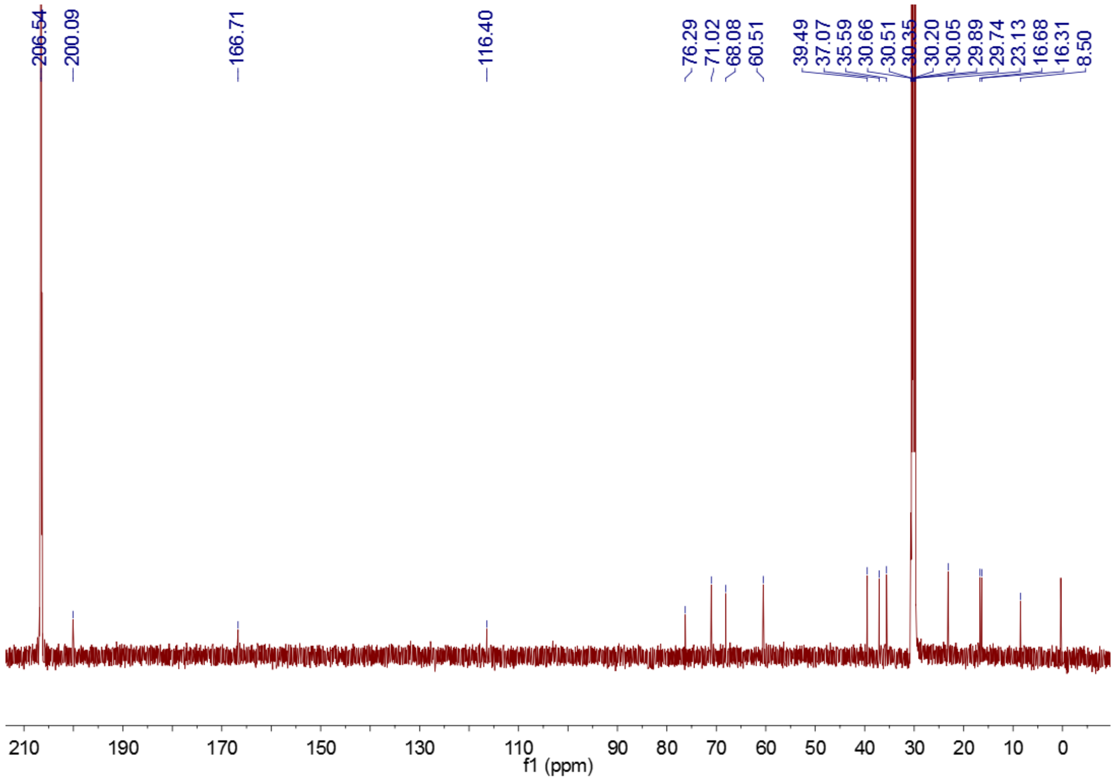

Figure S32. ^{13}C NMR spectrum (125 MHz, CD_3COCD_3) of **4**.

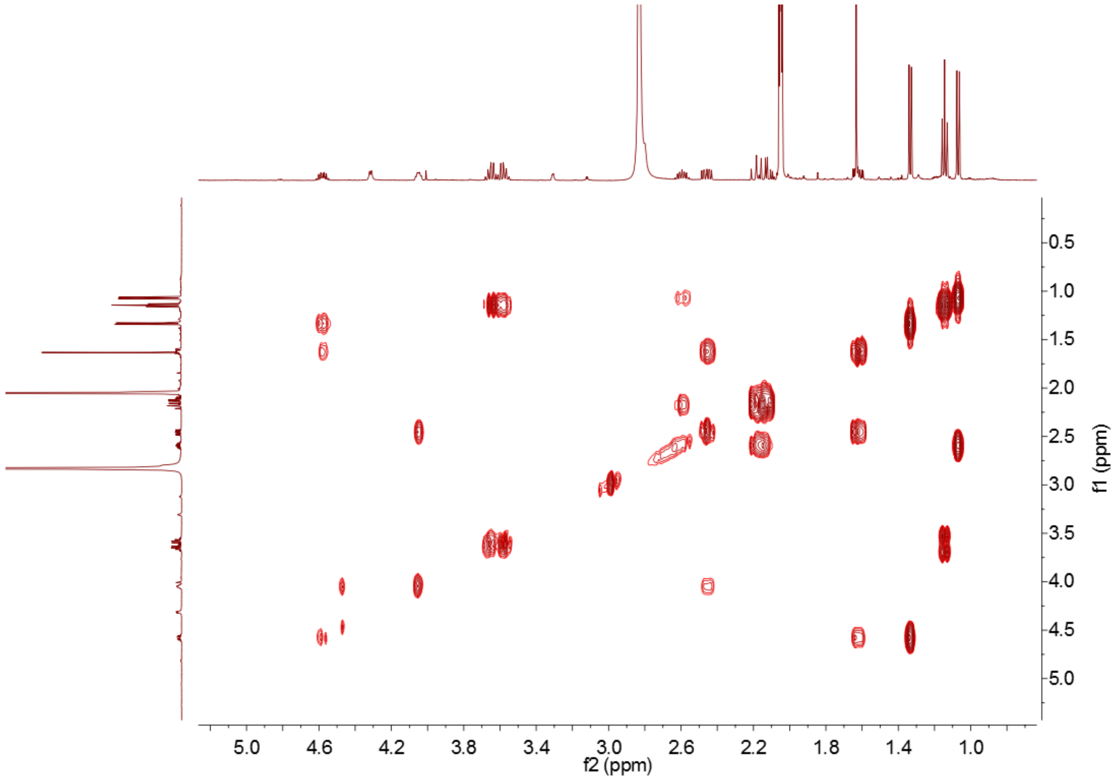

Figure S33. ^{1}H-^{1}H COSY spectrum of **4**.

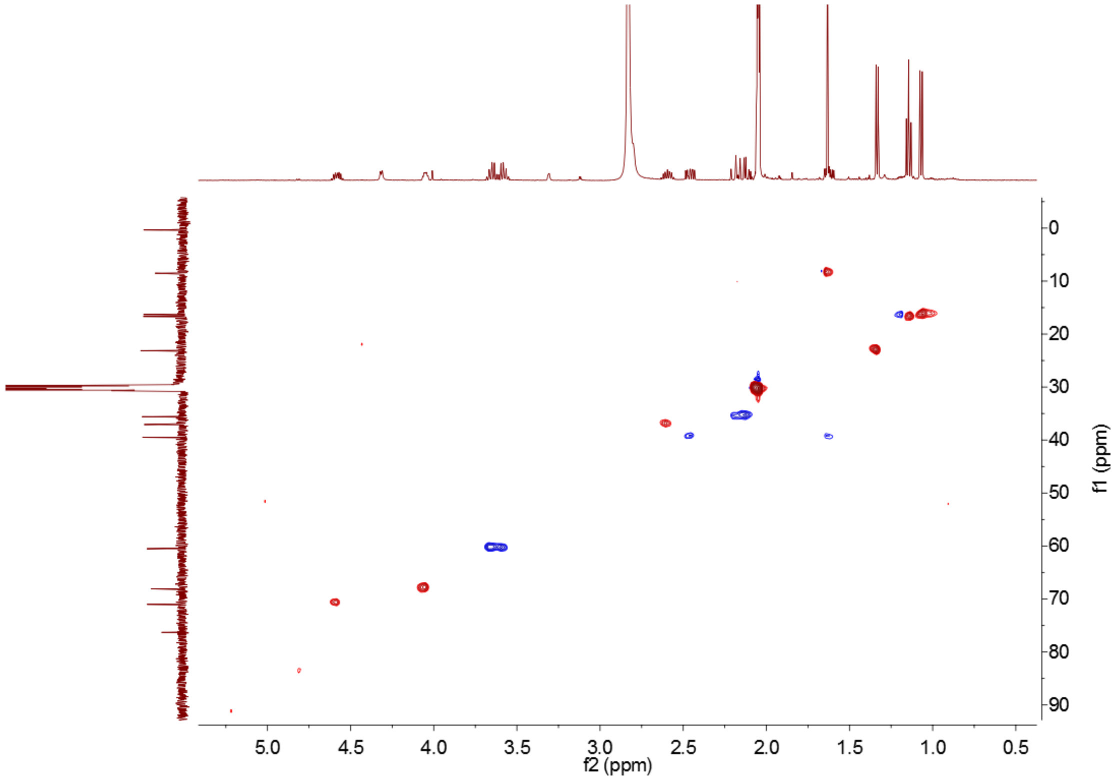

Figure S34. HSQC spectrum of **4**.

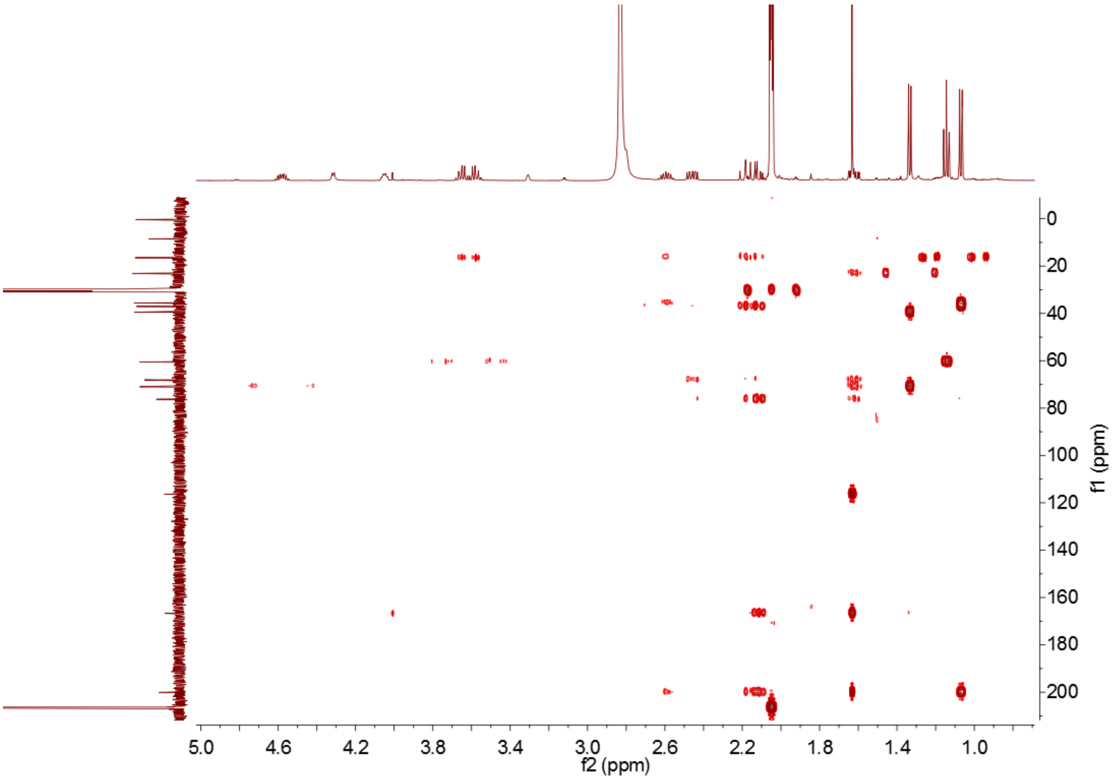

Figure S35. HMBC spectrum of **4**.

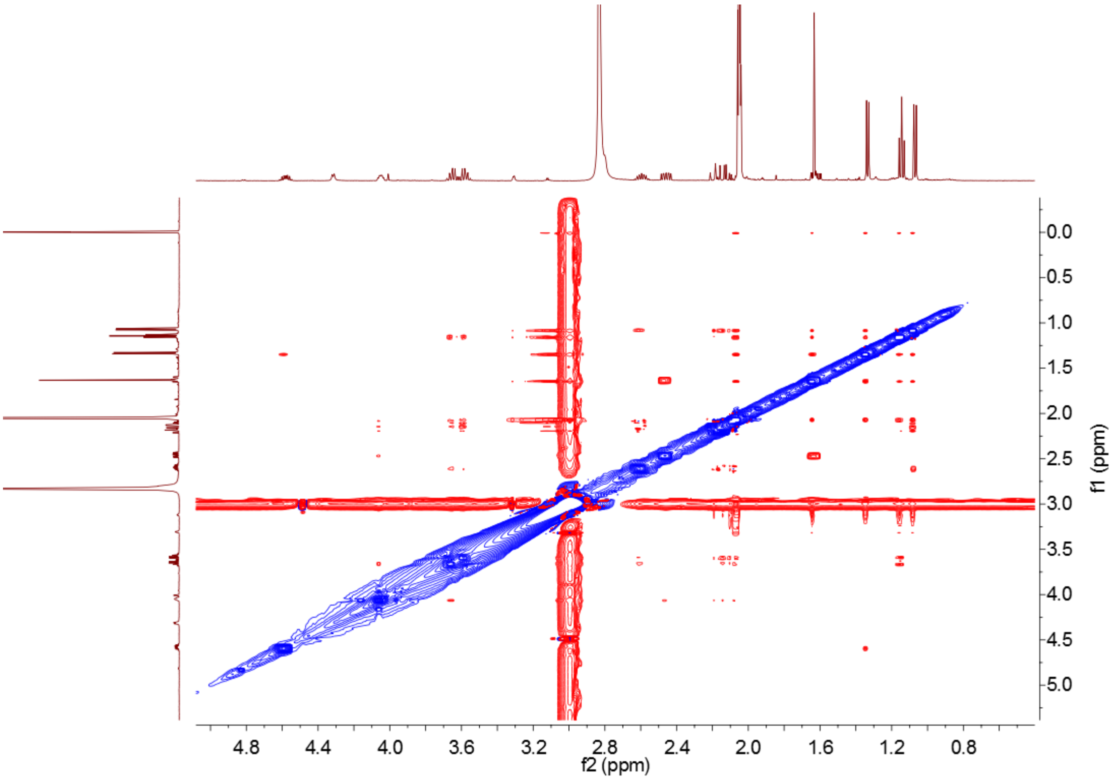

Figure S36. NOESY spectrum of **4**.

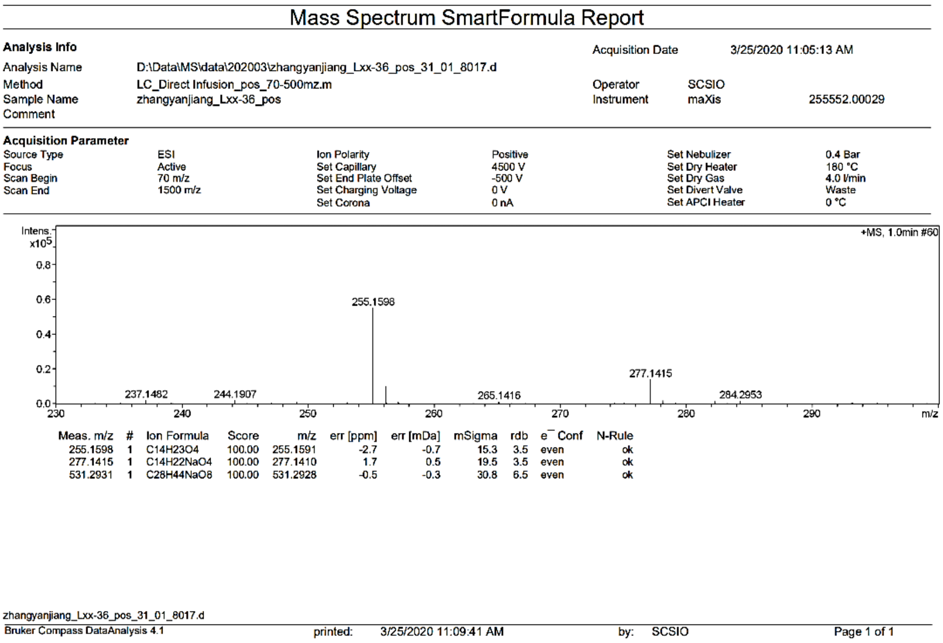

Mass Spectrum SmartFormula Report

**Analysis Info**		Acquisition Date	3/25/2020 11:05:13 AM
Analysis Name	D:\Data\MS\data\202003\zhangyanjiang_Lxx-36_pos_31_01_8017.d		
Method	LC_Direct Infusion_pos_70-500mz.m	Operator	SCSIO
Sample Name	zhangyanjiang_Lxx-36_pos	Instrument	maXis 255552.00029
Comment			

**Acquisition Parameter**					
Source Type	ESI	Ion Polarity	Positive	Set Nebulizer	0.4 Bar
Focus	Active	Set Capillary	4500 V	Set Dry Heater	180 °C
Scan Begin	70 m/z	Set End Plate Offset	-500 V	Set Dry Gas	4.0 l/min
Scan End	1500 m/z	Set Charging Voltage	0 V	Set Divert Valve	Waste
		Set Corona	0 nA	Set APCI Heater	0 °C

Meas. m/z	#	Ion Formula	Score	m/z	err [ppm]	err [mDa]	mSigma	rdb	e⁻ Conf	N-Rule
255.1598	1	C14H23O4	100.00	255.1591	-2.7	-0.7	15.3	3.5	even	ok
277.1415	1	C14H22NaO4	100.00	277.1410	1.7	0.5	19.5	3.5	even	ok
531.2931	1	C28H44NaO8	100.00	531.2928	-0.5	-0.3	30.8	6.5	even	ok

zhangyanjiang_Lxx-36_pos_31_01_8017.d
Bruker Compass DataAnalysis 4.1 printed: 3/25/2020 11:09:41 AM by: SCSIO Page 1 of 1

Figure S37. HRESIMS spectrum of **4**.

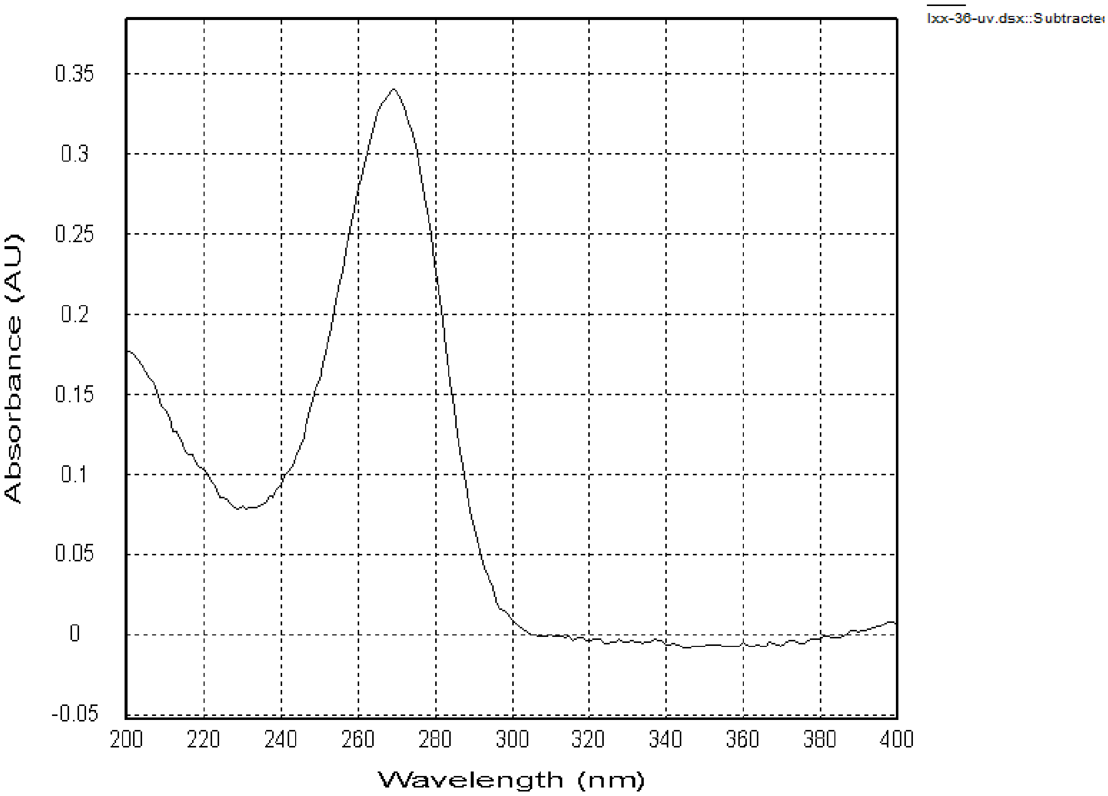

Figure S38. UV spectrum of **4**.

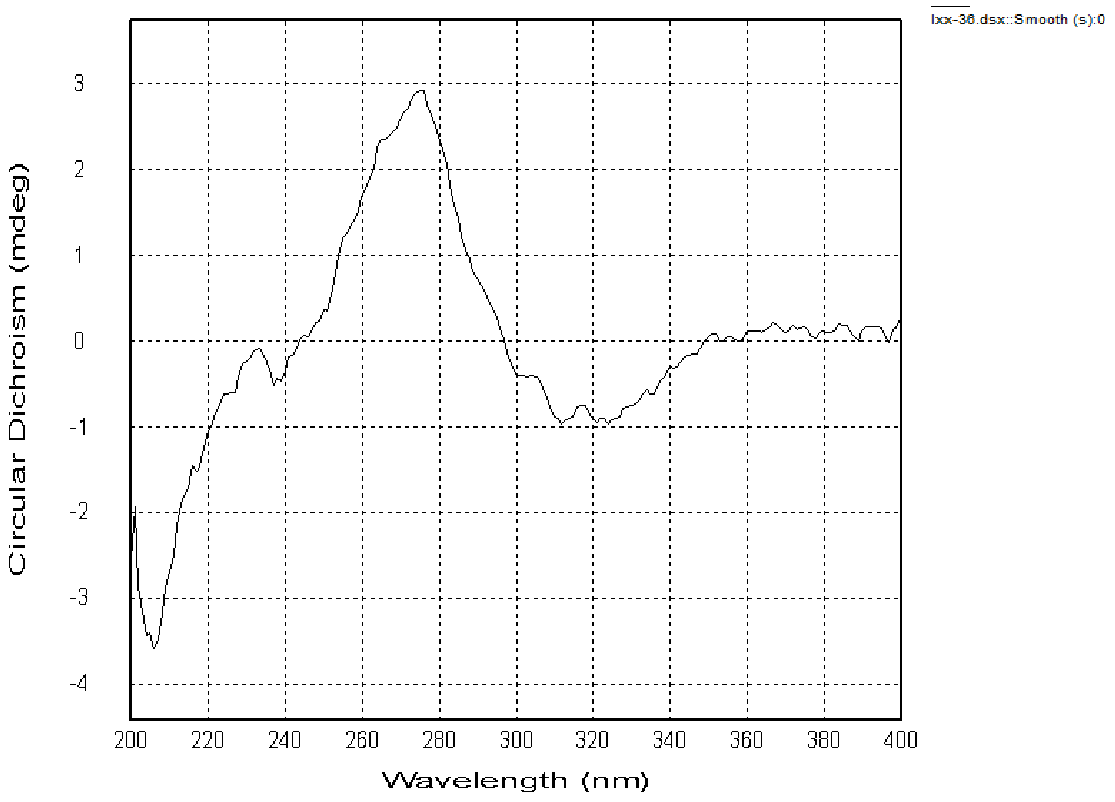

Figure S39. CD spectrum of **4**.

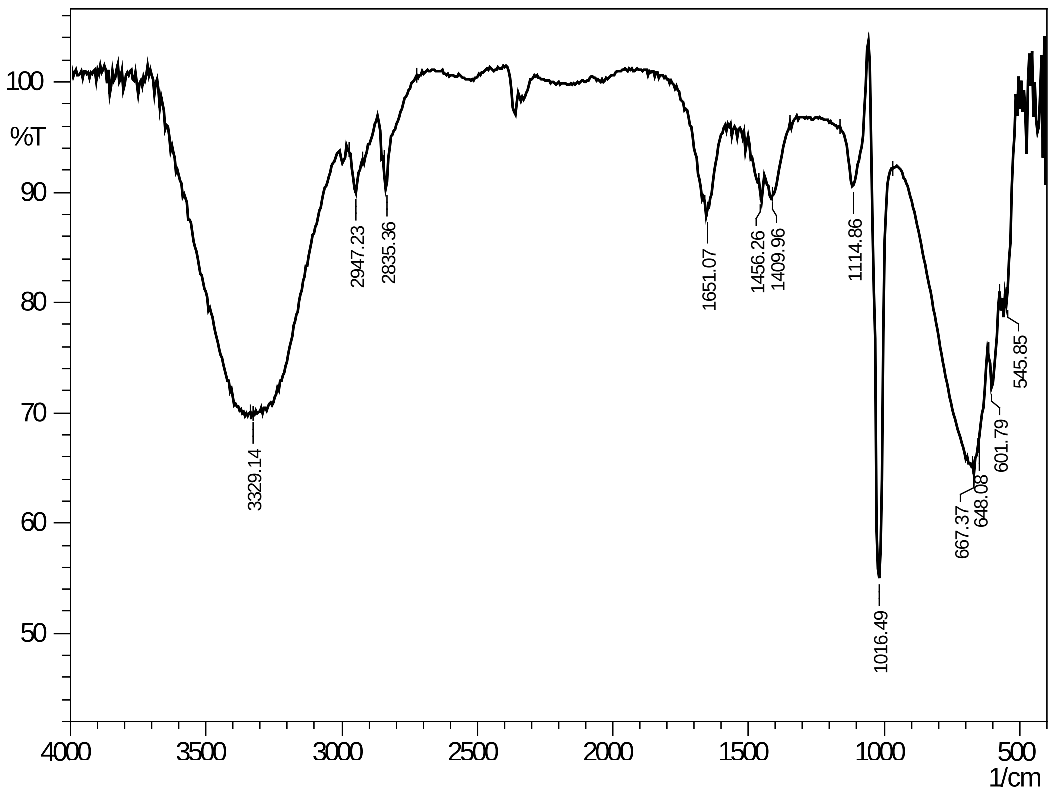

Figure S40. IR spectrum of **4**.

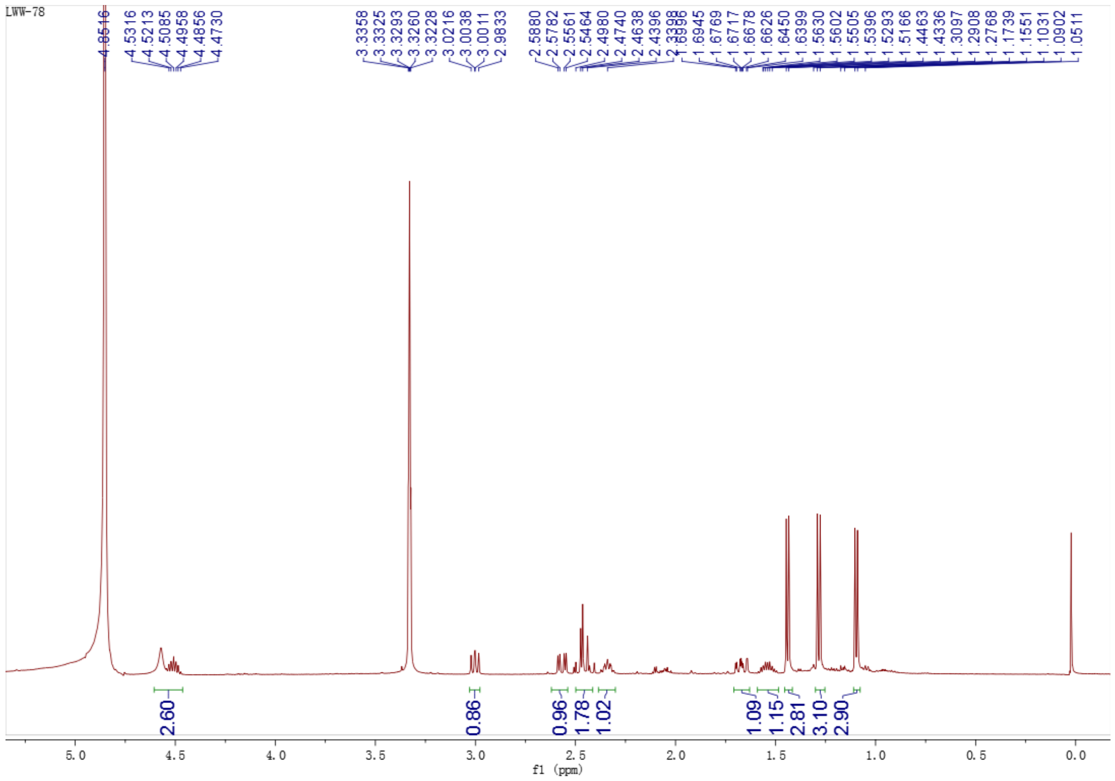

Figure S41. ^{1}H NMR spectrum (500 MHz, CD_3OD) of **5**.

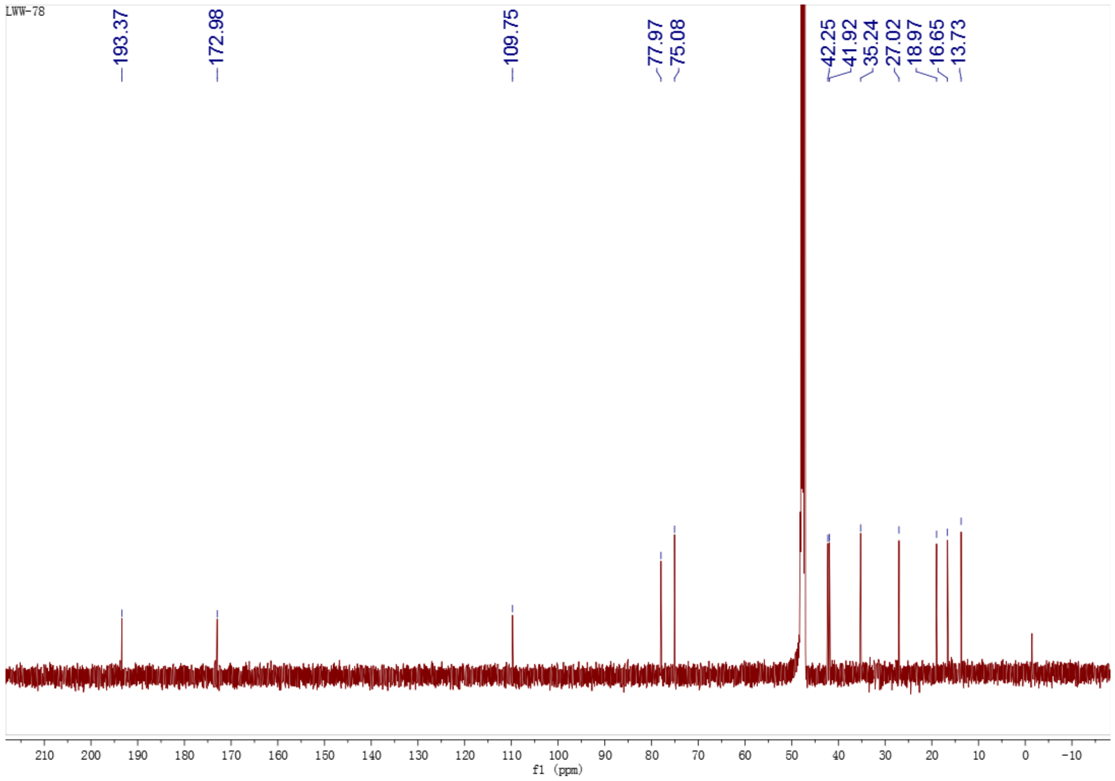

Figure S42. ^{13}C NMR spectrum (125 MHz, CD_3OD) of **5**.

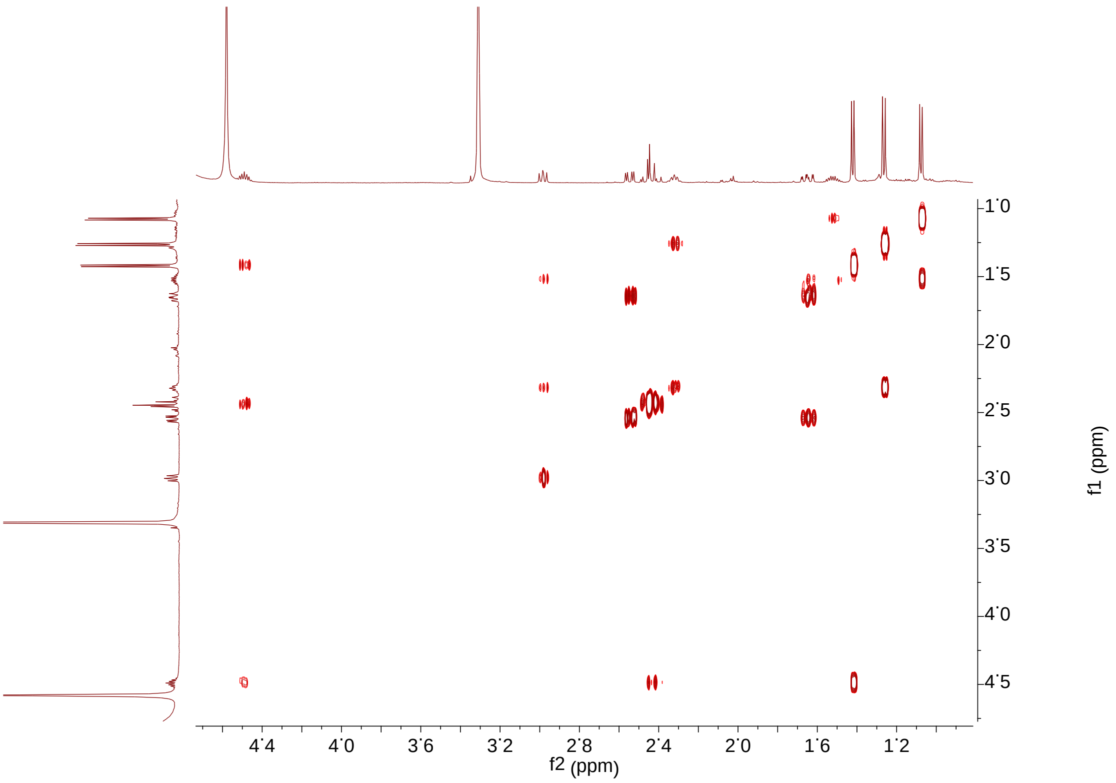

Figure S43. ^{1}H-^{1}H COSY spectrum of **5**.

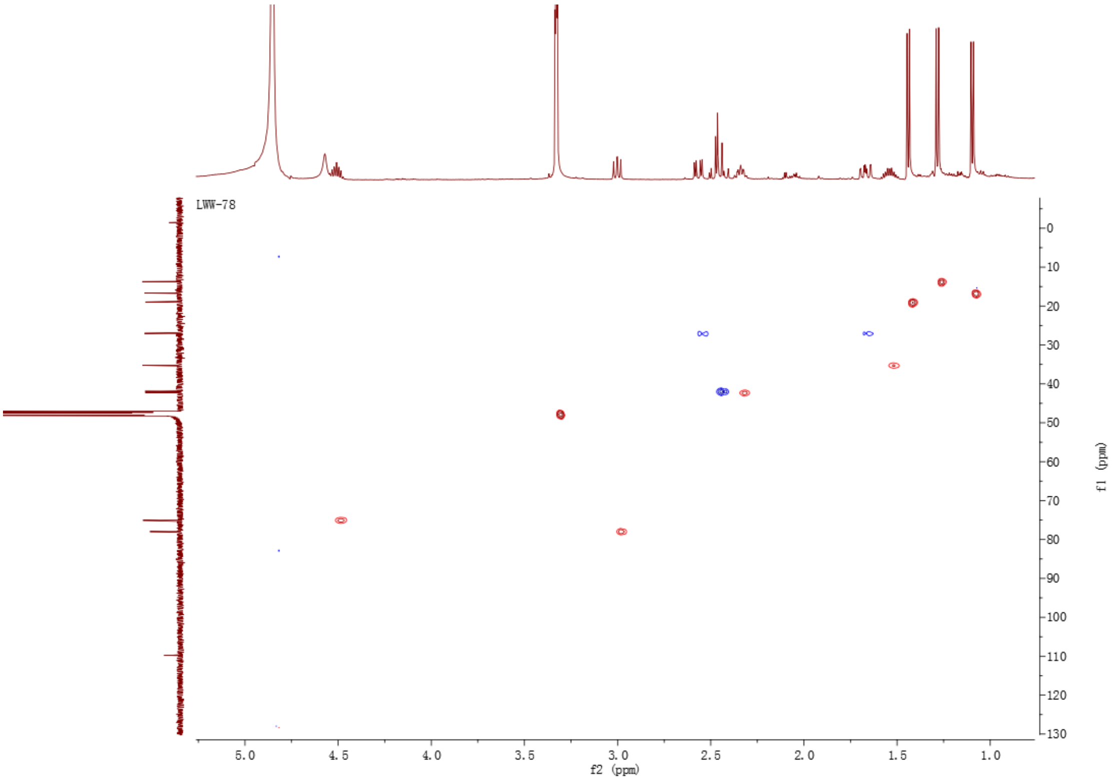

Figure S44. HSQC spectrum of **5**.

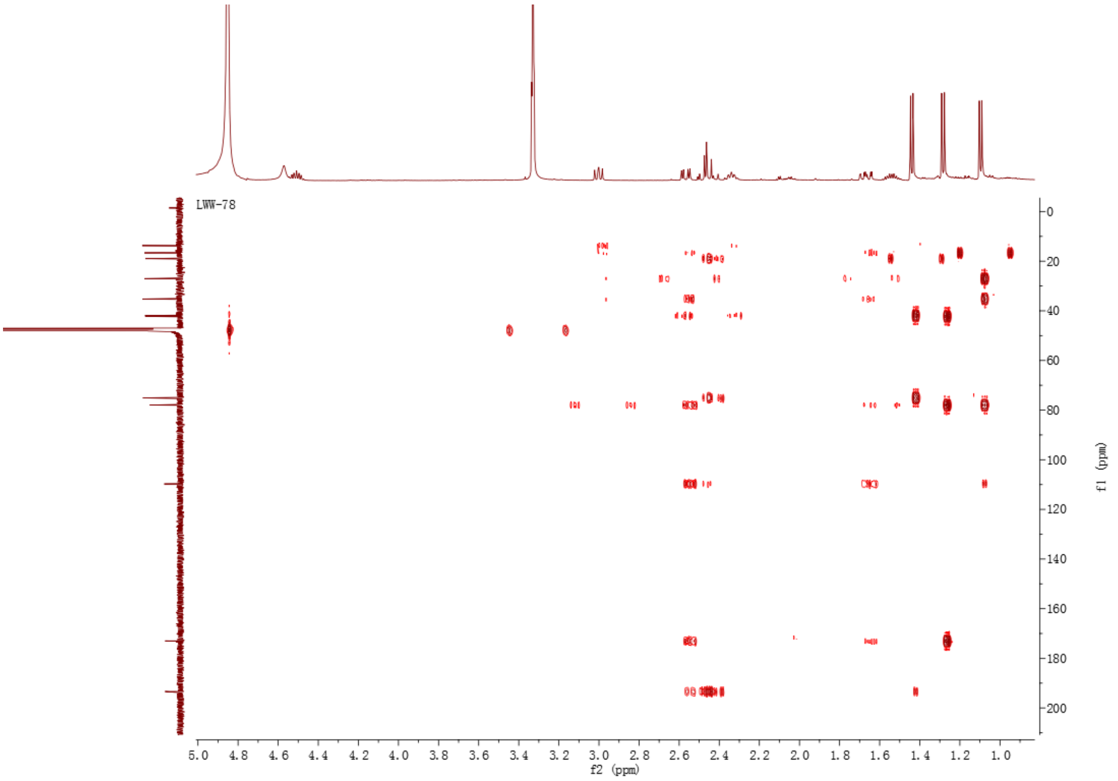

Figure S45. HMBC spectrum of **5**.

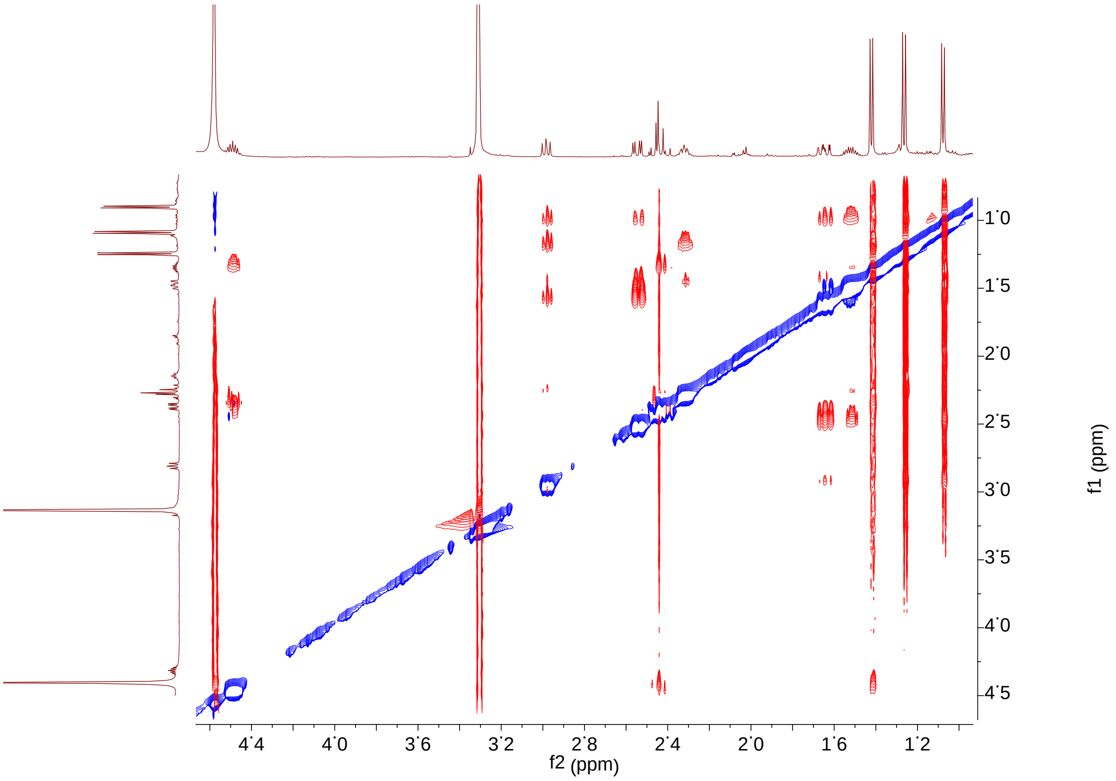

Figure S46. NOESY spectrum of **5**.

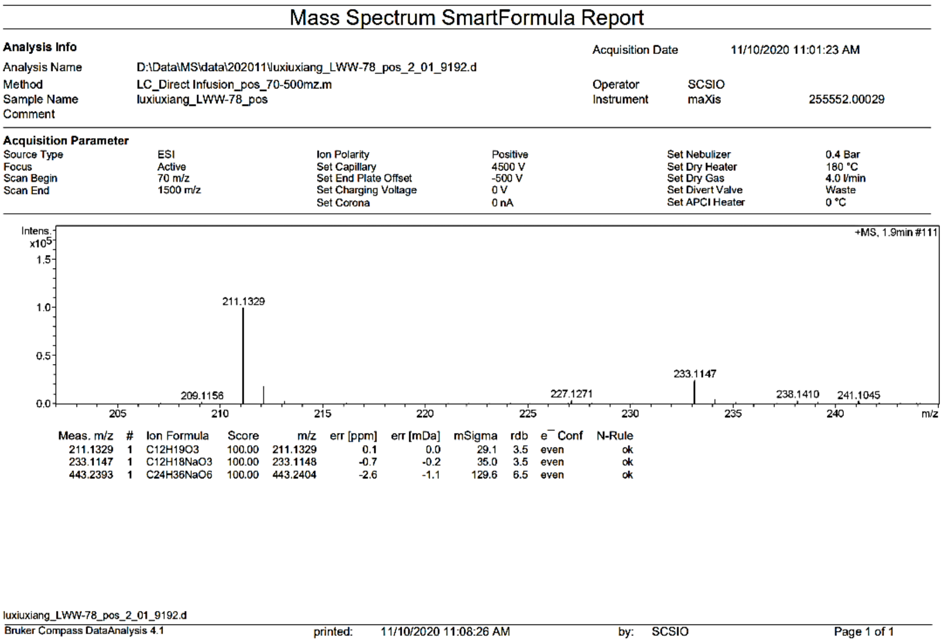

Mass Spectrum SmartFormula Report

Analysis Info

Analysis Name	D:\Data\MS\data\202011\luxiuxiang_LWW-78_pos_2_01_9192.d	Acquisition Date	11/10/2020 11:01:23 AM
Method	LC_Direct Infusion_pos_70-500mz.m	Operator	SCSIO
Sample Name	luxiuxiang_LWW-78_pos	Instrument	maXis 255552.00029
Comment			

Acquisition Parameter

Source Type	ESI	Ion Polarity	Positive	Set Nebulizer	0.4 Bar
Focus	Active	Set Capillary	4500 V	Set Dry Heater	180 °C
Scan Begin	70 m/z	Set End Plate Offset	-500 V	Set Dry Gas	4.0 l/min
Scan End	1500 m/z	Set Charging Voltage	0 V	Set Divert Valve	Waste
		Set Corona	0 nA	Set APCI Heater	0 °C

Meas. m/z	#	Ion Formula	Score	m/z	err [ppm]	err [mDa]	mSigma	rdb	e⁻ Conf	N-Rule
211.1329	1	C12H19O3	100.00	211.1329	0.1	0.0	29.1	3.5	even	ok
233.1147	1	C12H18NaO3	100.00	233.1148	-0.7	-0.2	35.0	3.5	even	ok
443.2393	1	C24H36NaO6	100.00	443.2404	-2.6	-1.1	129.6	6.5	even	ok

luxiuxiang_LWW-78_pos_2_01_9192.d
Bruker Compass DataAnalysis 4.1 printed: 11/10/2020 11:08:26 AM by: SCSIO Page 1 of 1

Figure S47. HRESIMS spectrum of **5**.

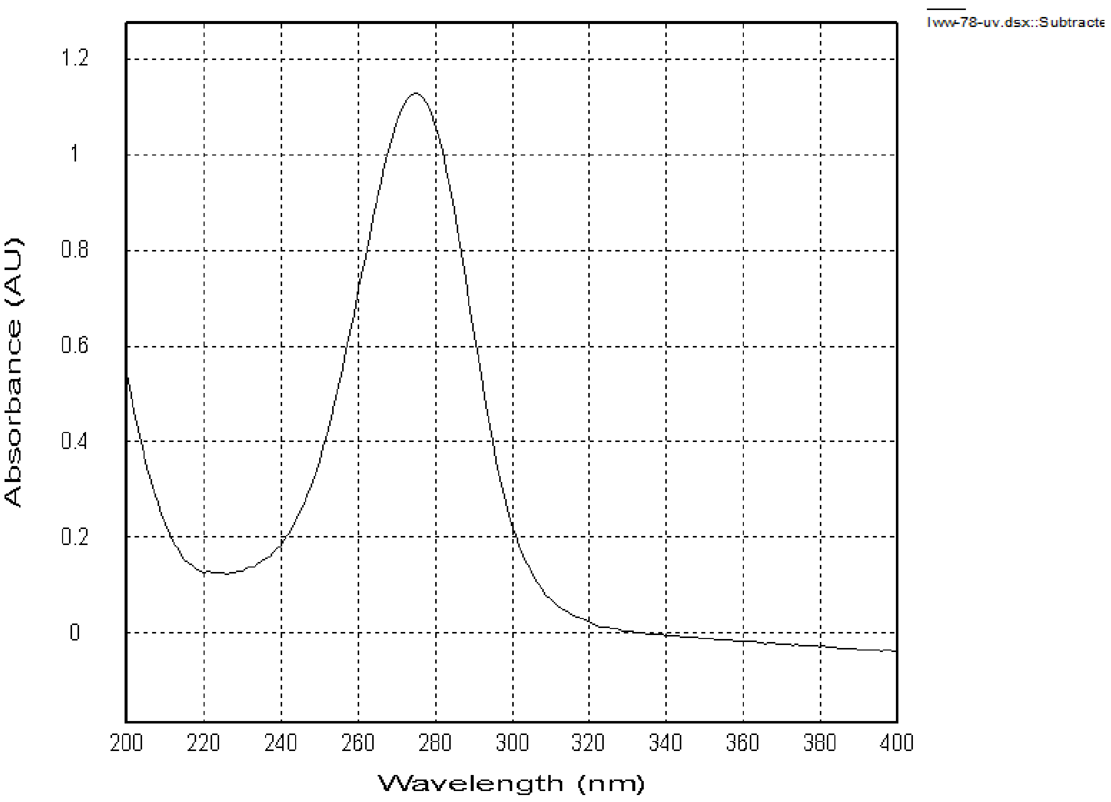

Figure S48. UV spectrum of **5.**

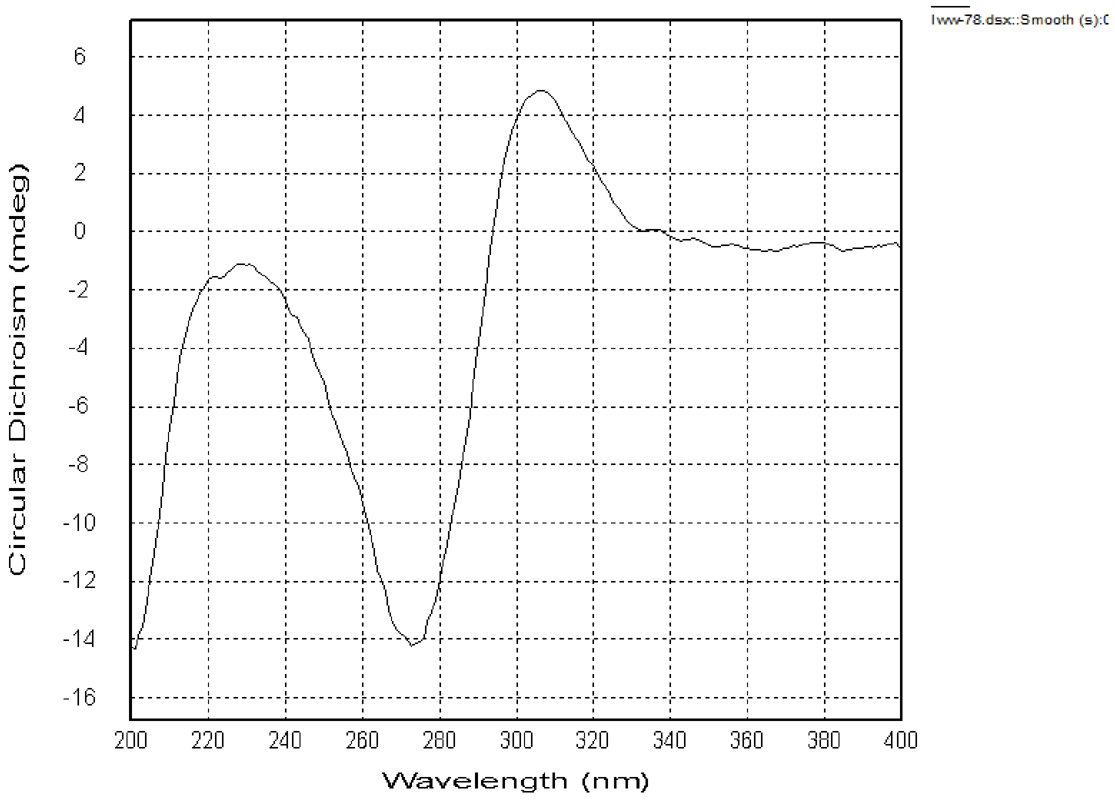

Figure S49. CD spectrum of **5**.

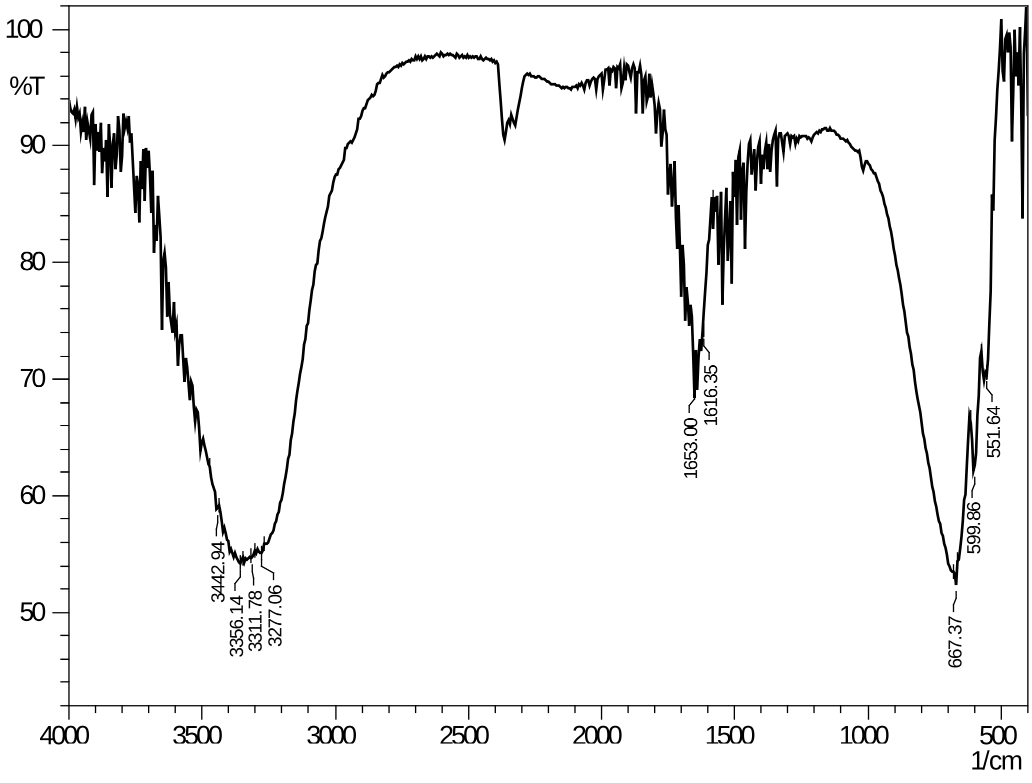

Figure S50. IR spectrum of **5**.

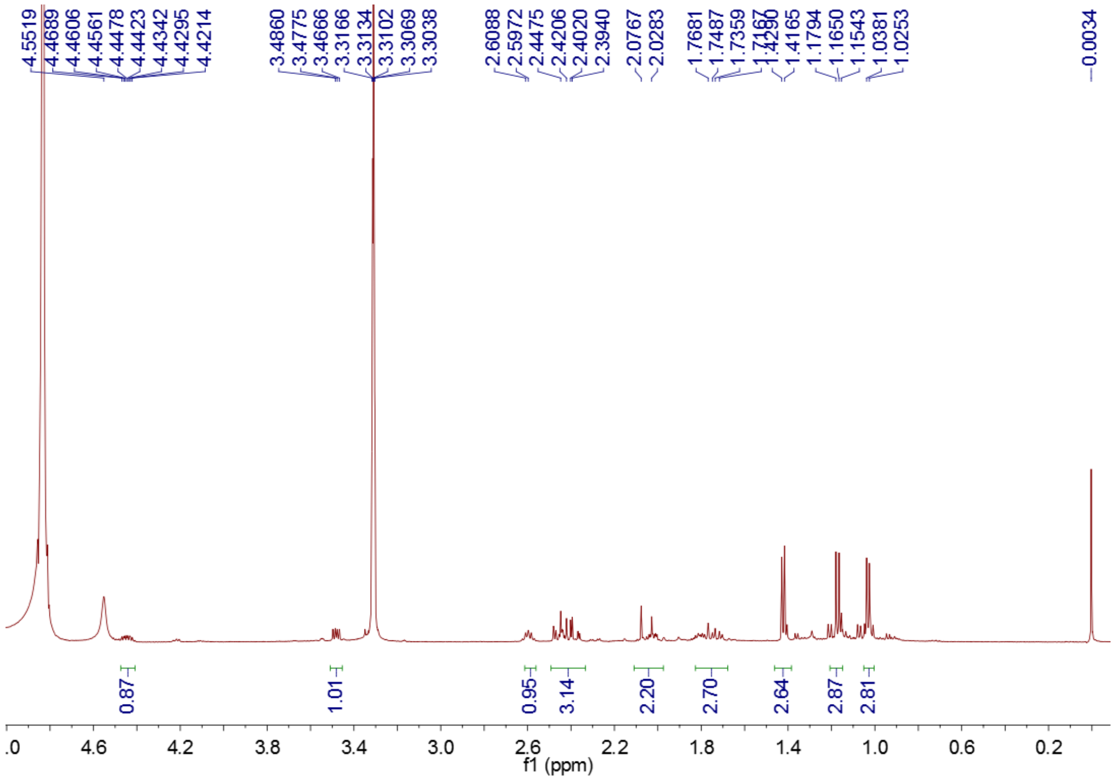

Figure S51. ^{1}H NMR spectrum (500 MHz, CD_3OD) of **6**.

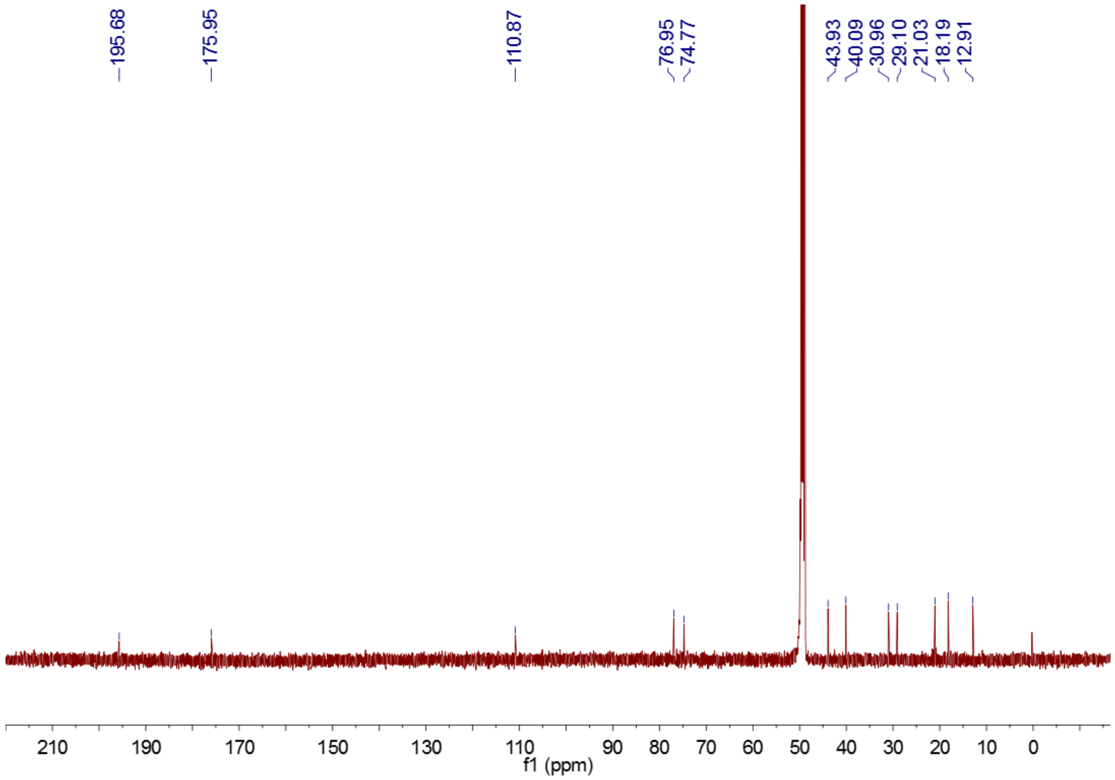

Figure S52. ^{13}C NMR spectrum (125 MHz, CD_3OD) of **6**.

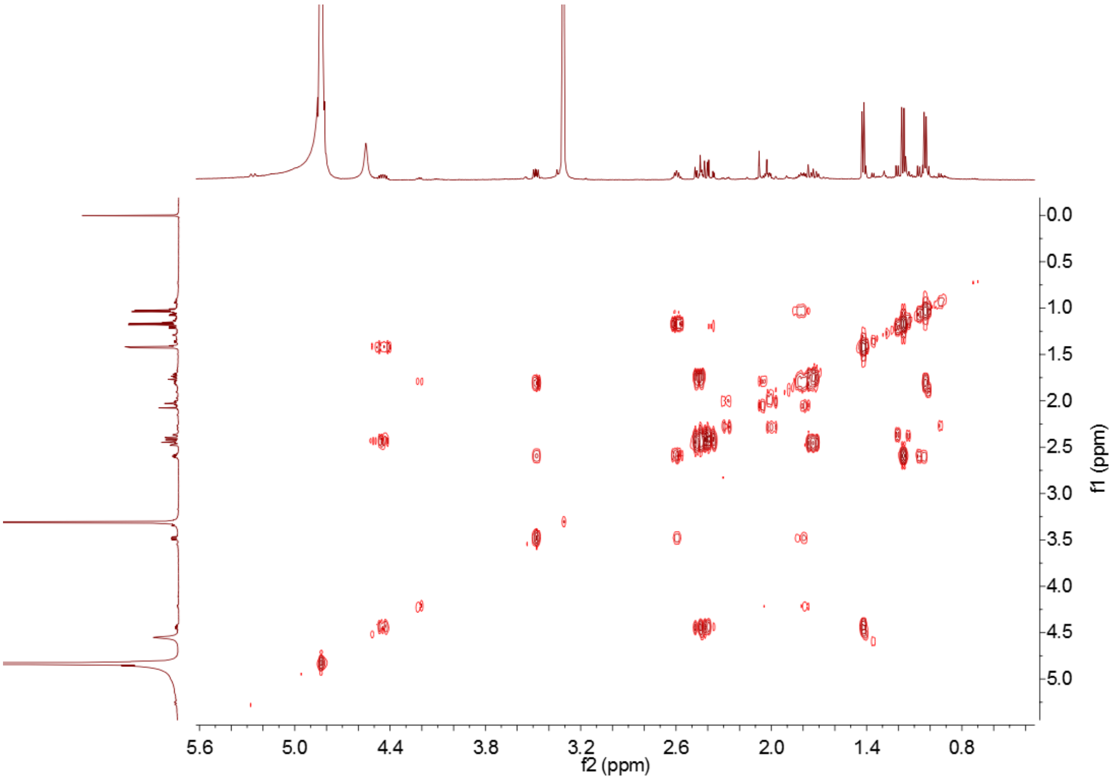

Figure S53. ^{1}H-^{1}H COSY spectrum of **6**.

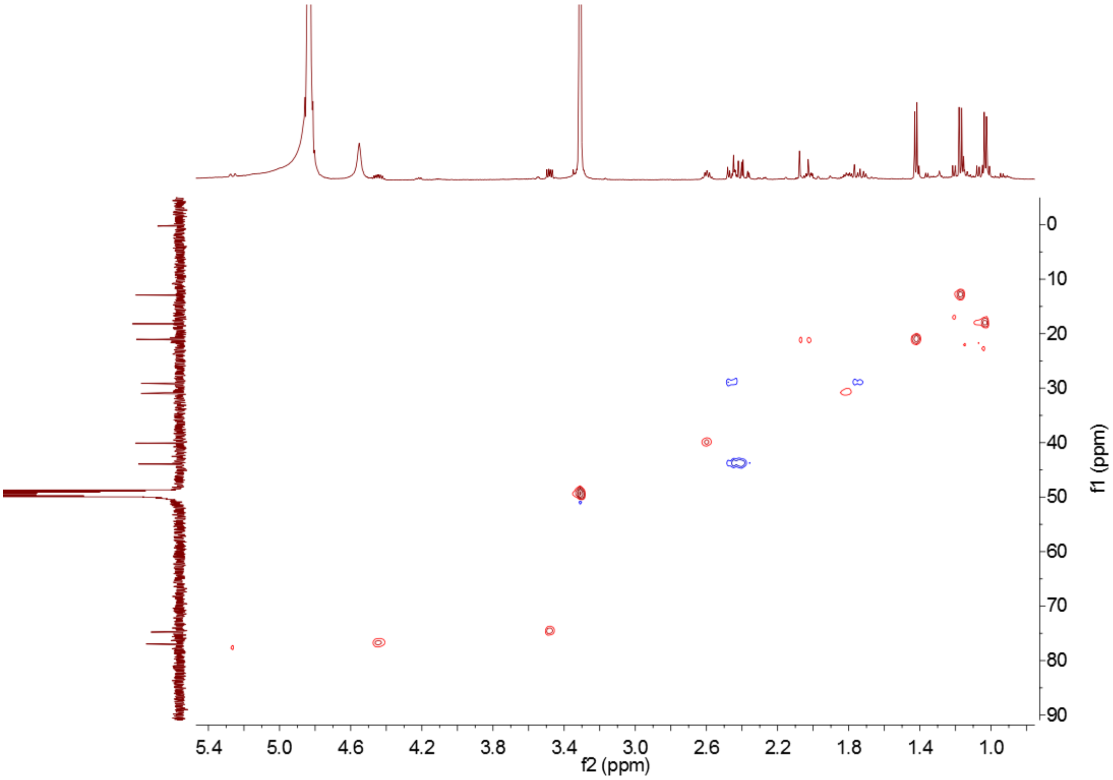

Figure S54. HSQC spectrum of **6**.

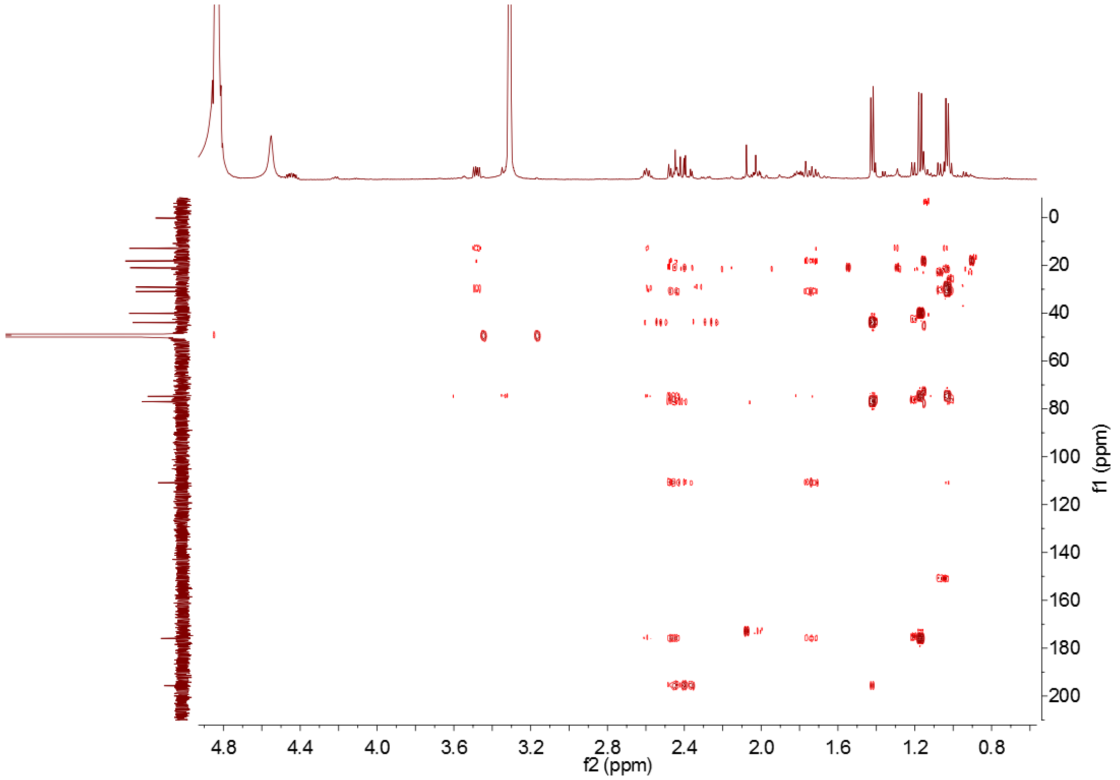

Figure S55. HMBC spectrum of **6**.

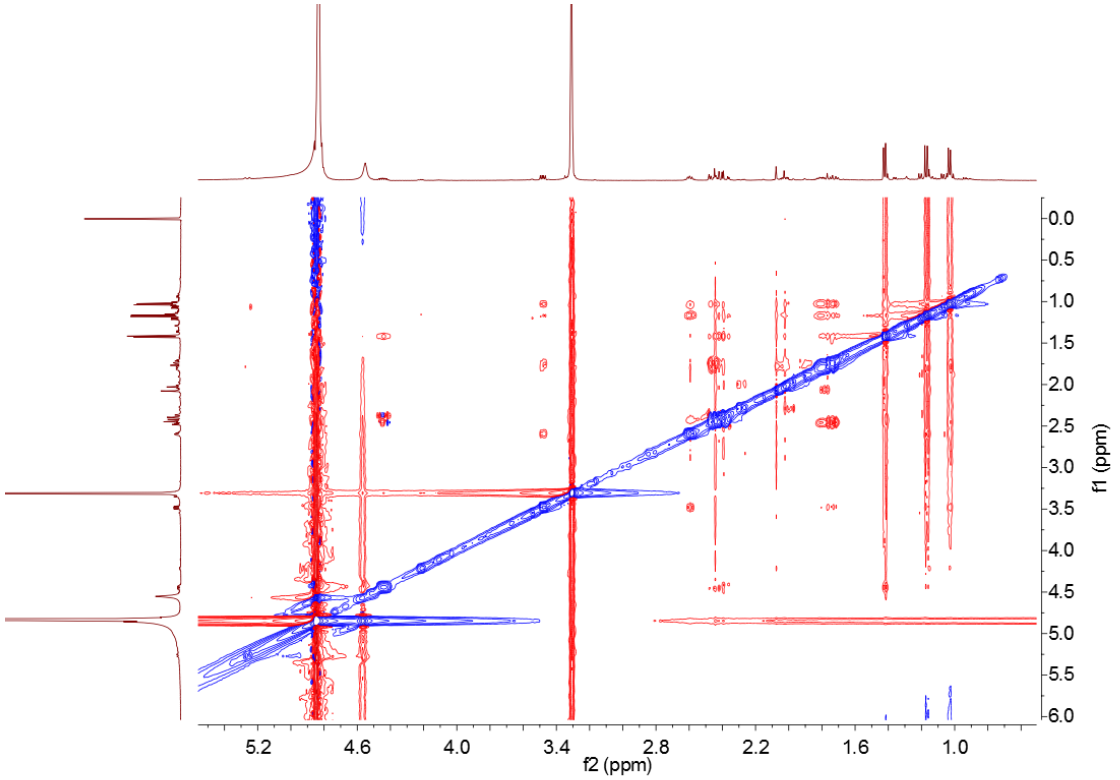

Figure S56. NOESY spectrum of **6**.

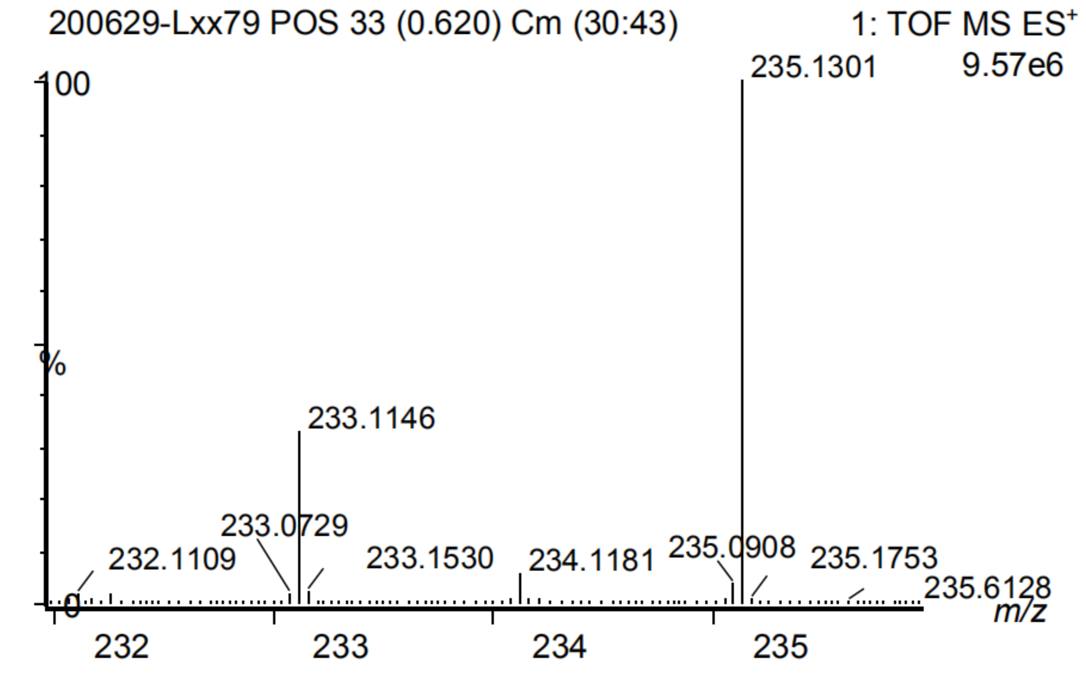

Figure S57. HRESIMS spectrum of **6**.

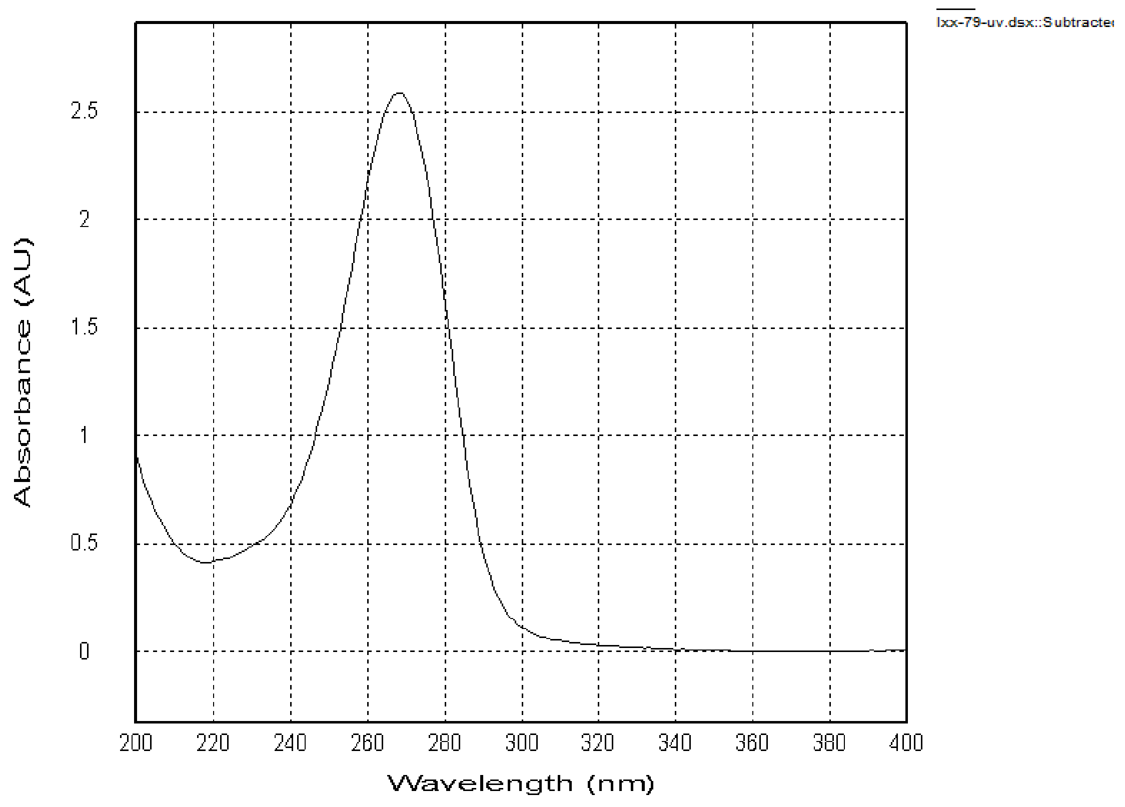

Figure S58. UV spectrum of **6**.

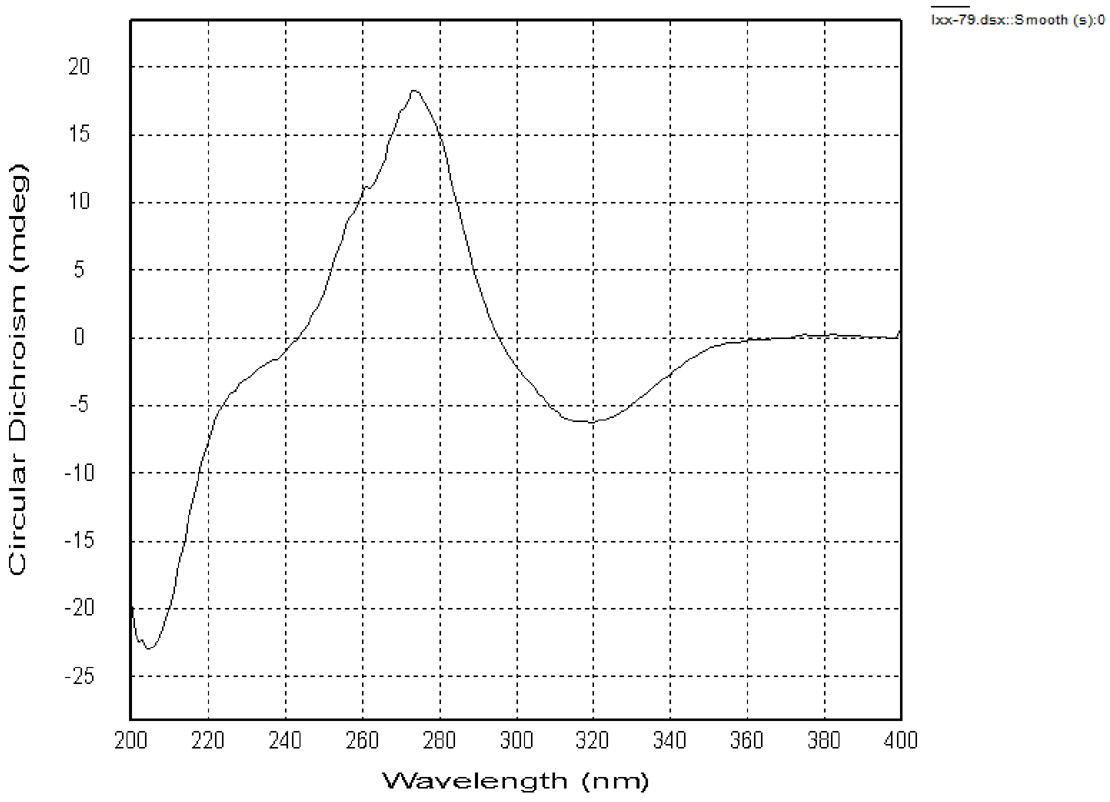

Figure S59. CD spectrum of **6**.

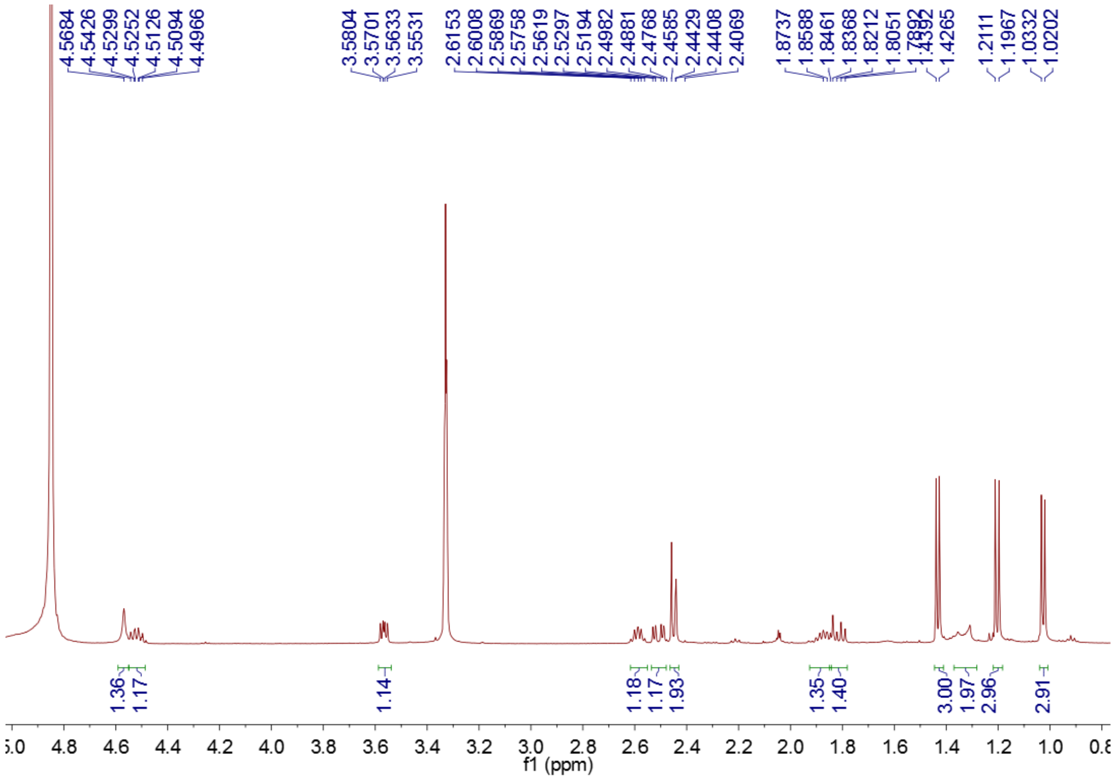

Figure S60. ^{1}H NMR spectrum (500 MHz, CD_3OD) of **7**.

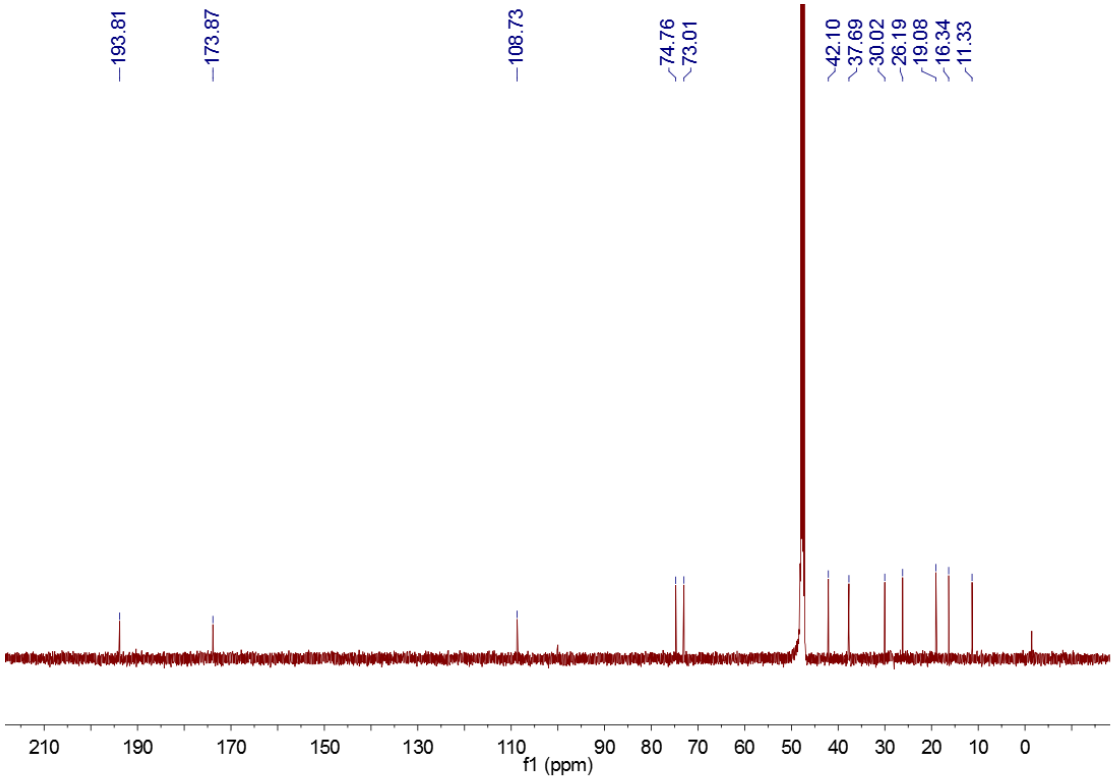

Figure S61. ^{13}C NMR spectrum (125 MHz, CD_3OD) of **7**.

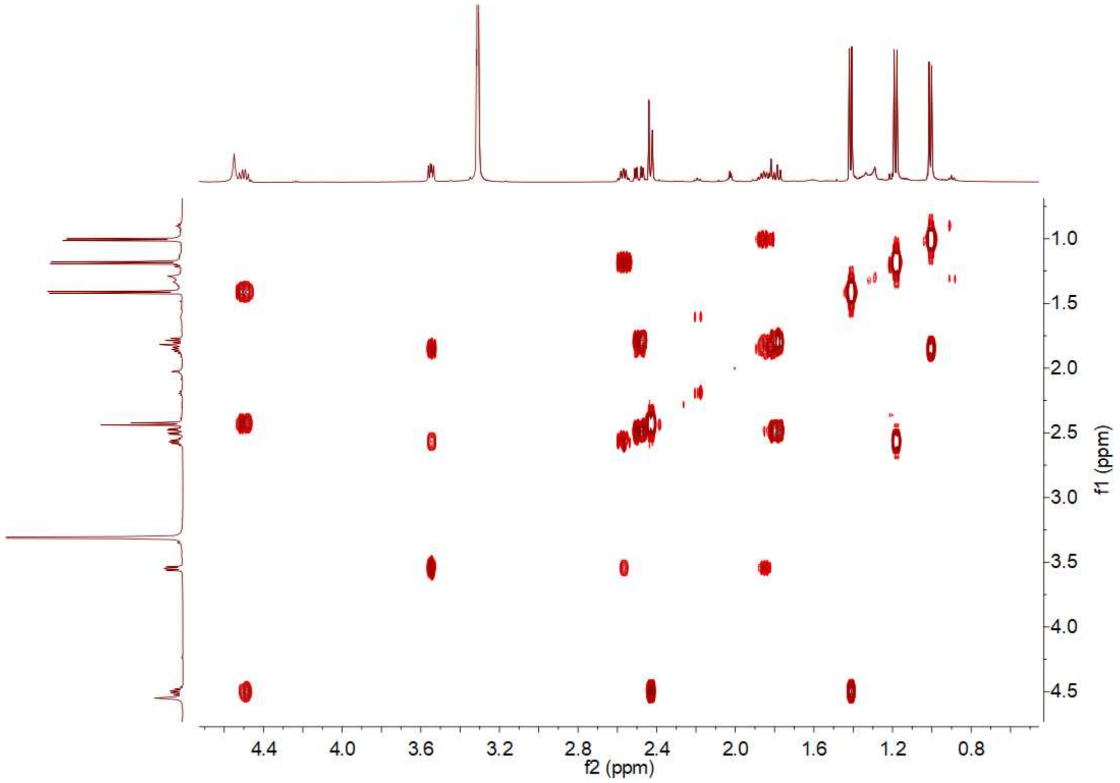

Figure S62. ^{1}H-^{1}H COSY spectrum of **7**.

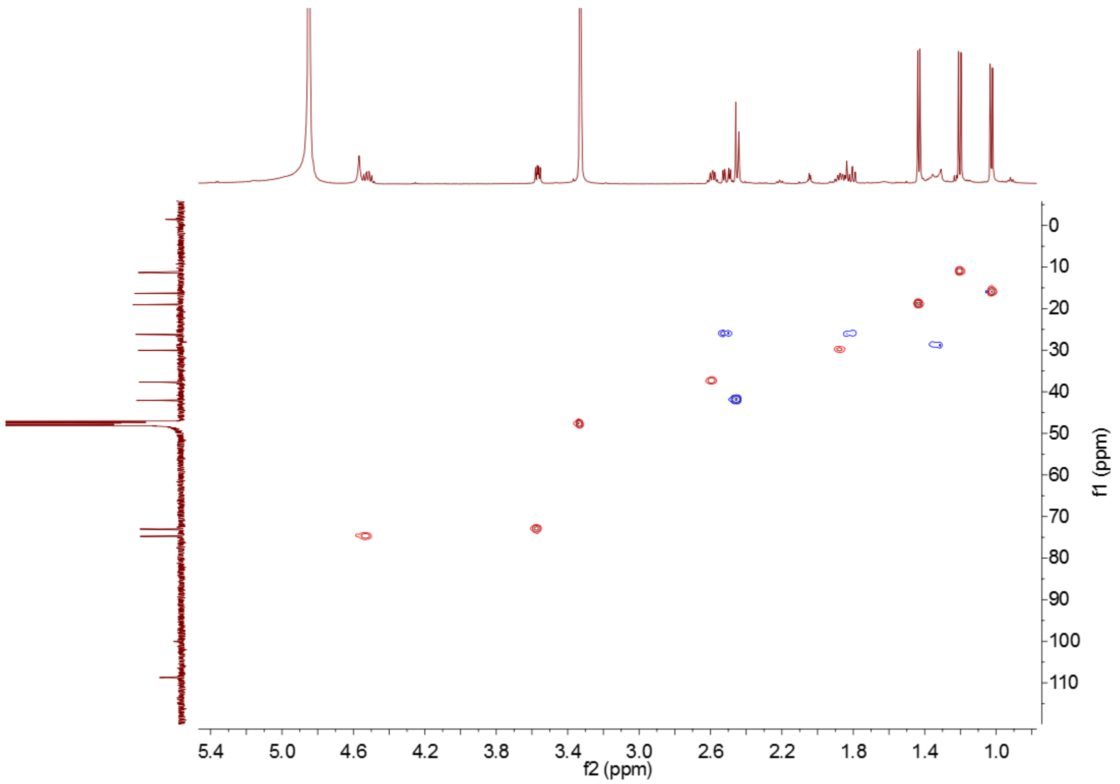

Figure S63. HSQC spectrum of **7**.

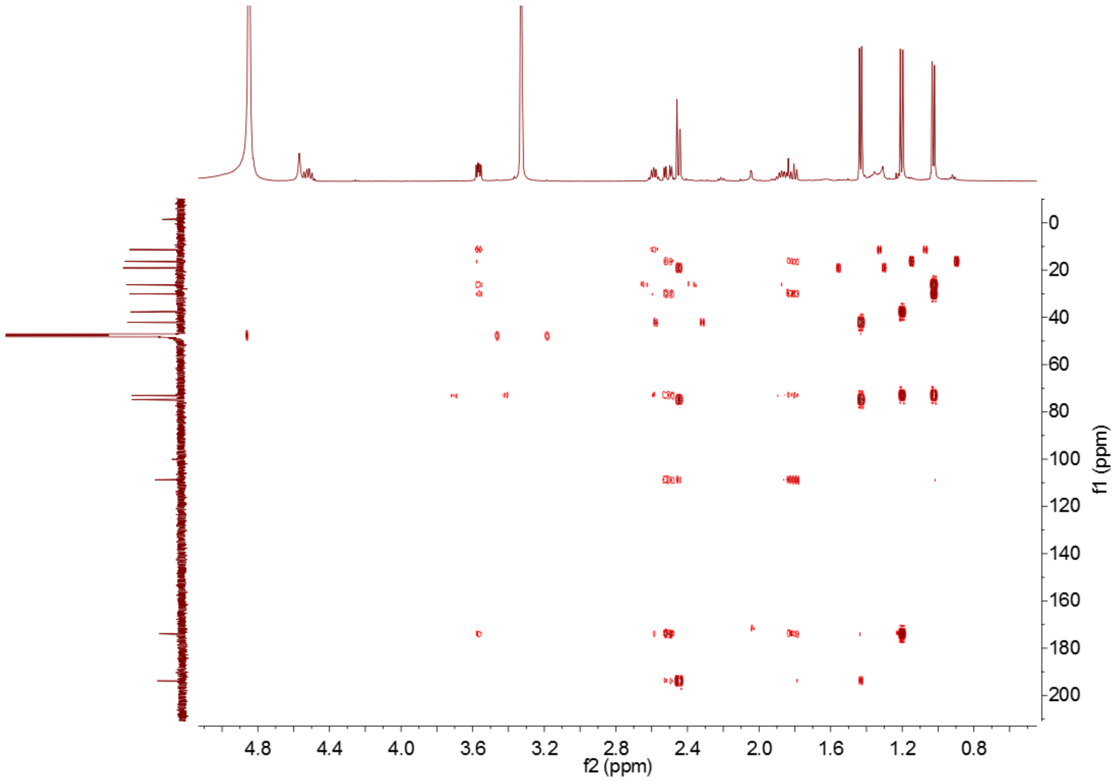

Figure S64. HMBC spectrum of **7**.

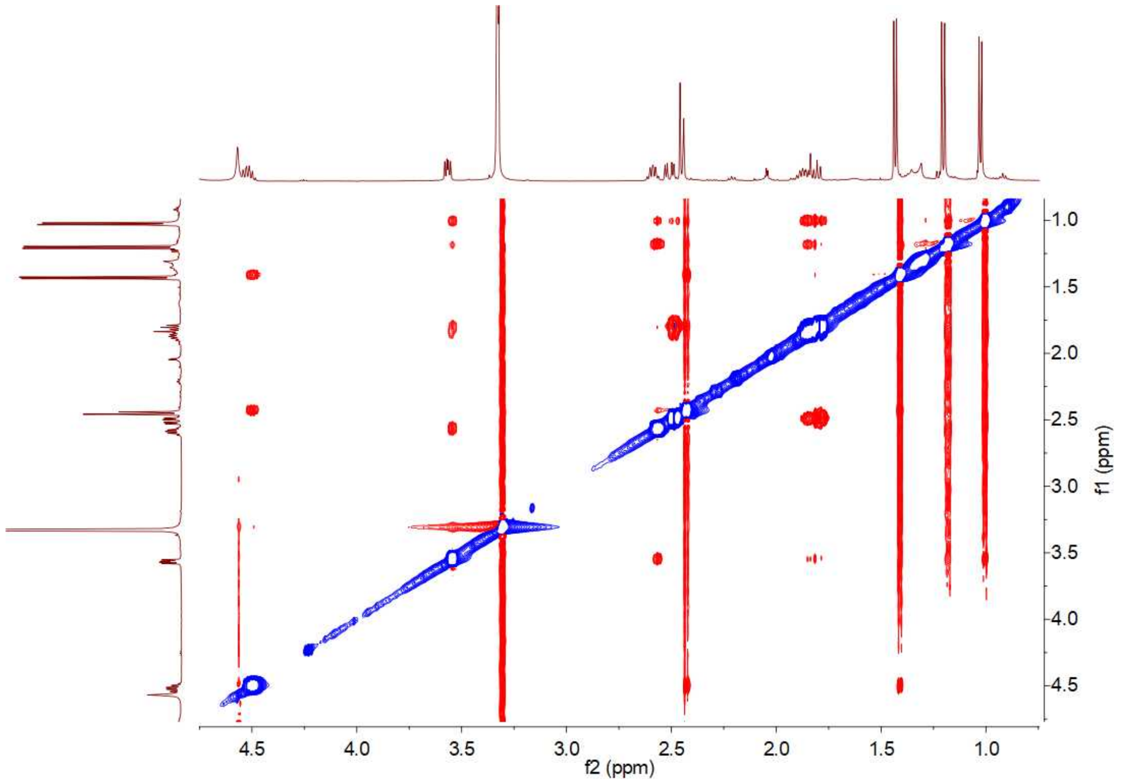

Figure S65. NOESY spectrum of **7**.

Mass Spectrum SmartFormula Report

Analysis Info

Analysis Name	D:\Data\MS\data\202008\liuxiuxiang_LWW-81_pos_73_01_8733.d	Acquisition Date	8/25/2020 9:29:30 AM
Method	LC_Direct Infusion_pos_70-500mz.m	Operator	SCSIO
Sample Name	liuxiuxiang_LWW-81_pos	Instrument	maXis 255552.00029
Comment			

Acquisition Parameter

Source Type	ESI	Ion Polarity	Positive	Set Nebulizer	0.4 Bar
Focus	Active	Set Capillary	4500 V	Set Dry Heater	180 °C
Scan Begin	70 m/z	Set End Plate Offset	-500 V	Set Dry Gas	4.0 l/min
Scan End	1500 m/z	Set Charging Voltage	0 V	Set Divert Valve	Waste
		Set Corona	0 nA	Set APCI Heater	0 °C

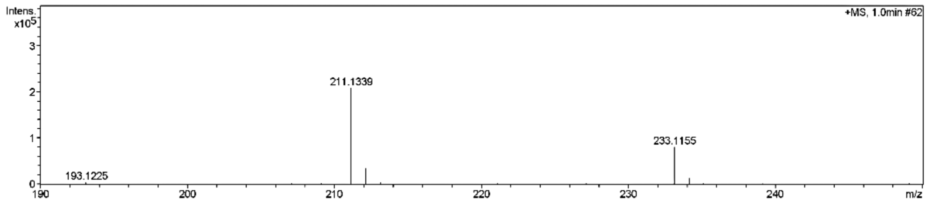

Meas. m/z	#	Ion Formula	Score	m/z	err [ppm]	err [mDa]	mSigma	rdb	e⁻ Conf	N-Rule
211.1339	1	C12H19O3	100.00	211.1329	4.6	1.0	20.0	3.5	even	ok
233.1155	1	C12H18NaO3	100.00	233.1148	-2.9	-0.7	15.9	3.5	even	ok
443.2406	1	C24H36NaO6	100.00	443.2404	0.4	0.2	21.9	6.5	even	ok

liuxiuxiang_LWW-81_pos_73_01_8733.d
Bruker Compass DataAnalysis 4.1 printed: 8/25/2020 9:39:11 AM by: SCSIO Page 1 of 1

Figure S66. HRESIMS spectrum of **7**.

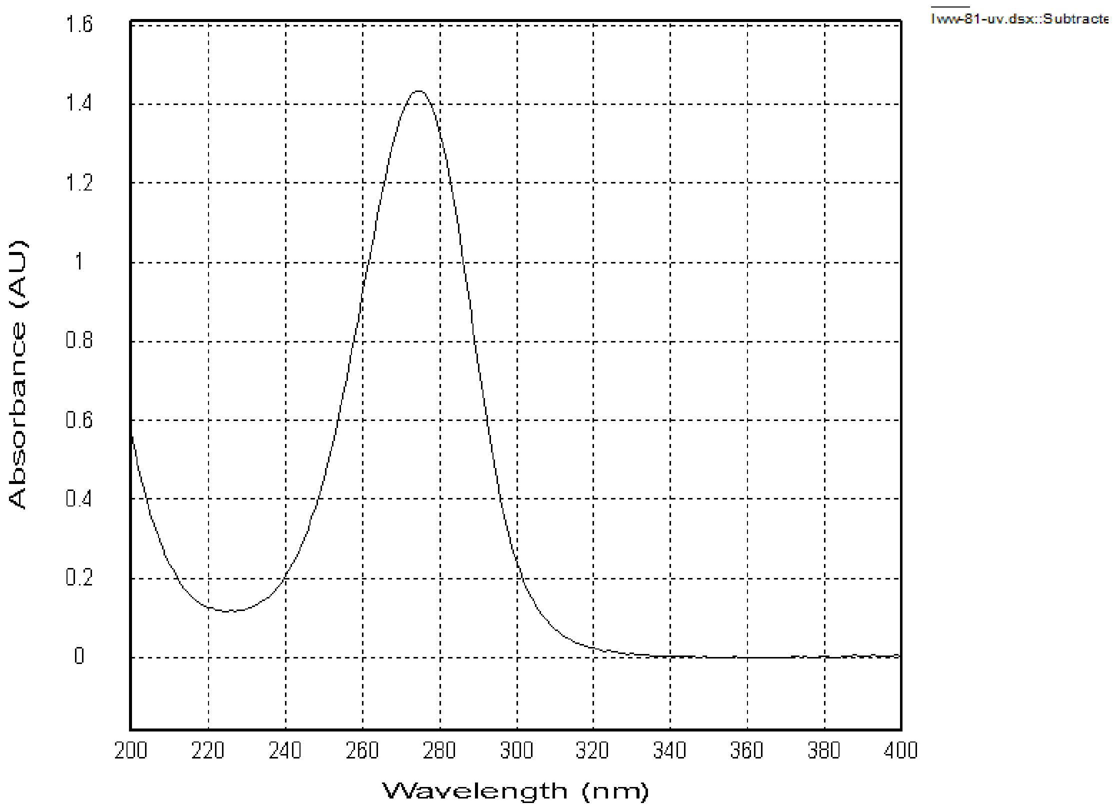

Figure S67. UV spectrum of **7.**

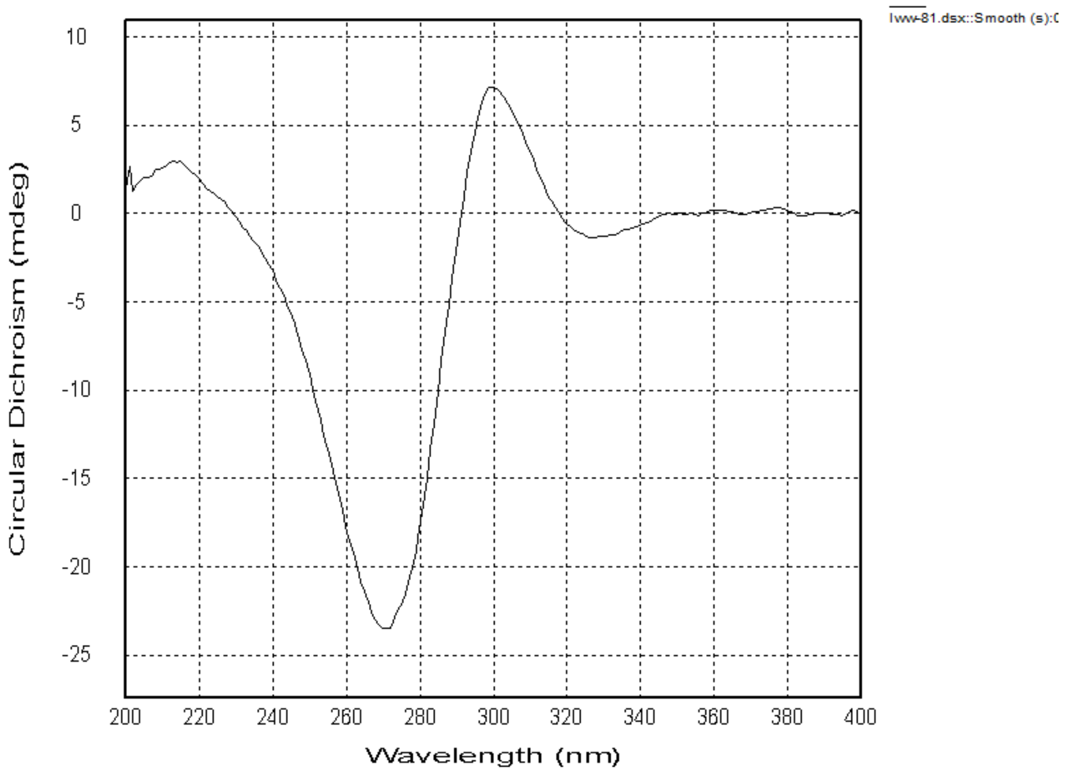

Figure S68. CD spectrum of **7**.

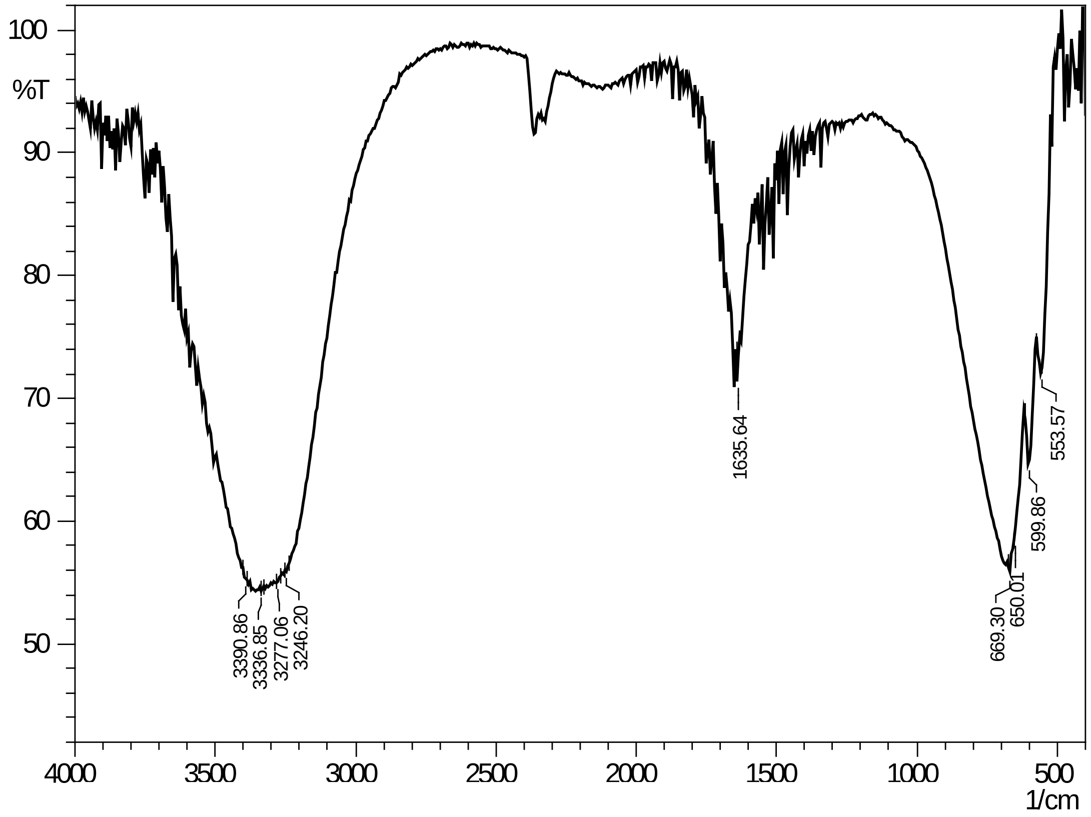

Figure S69. IR spectrum of **7**.

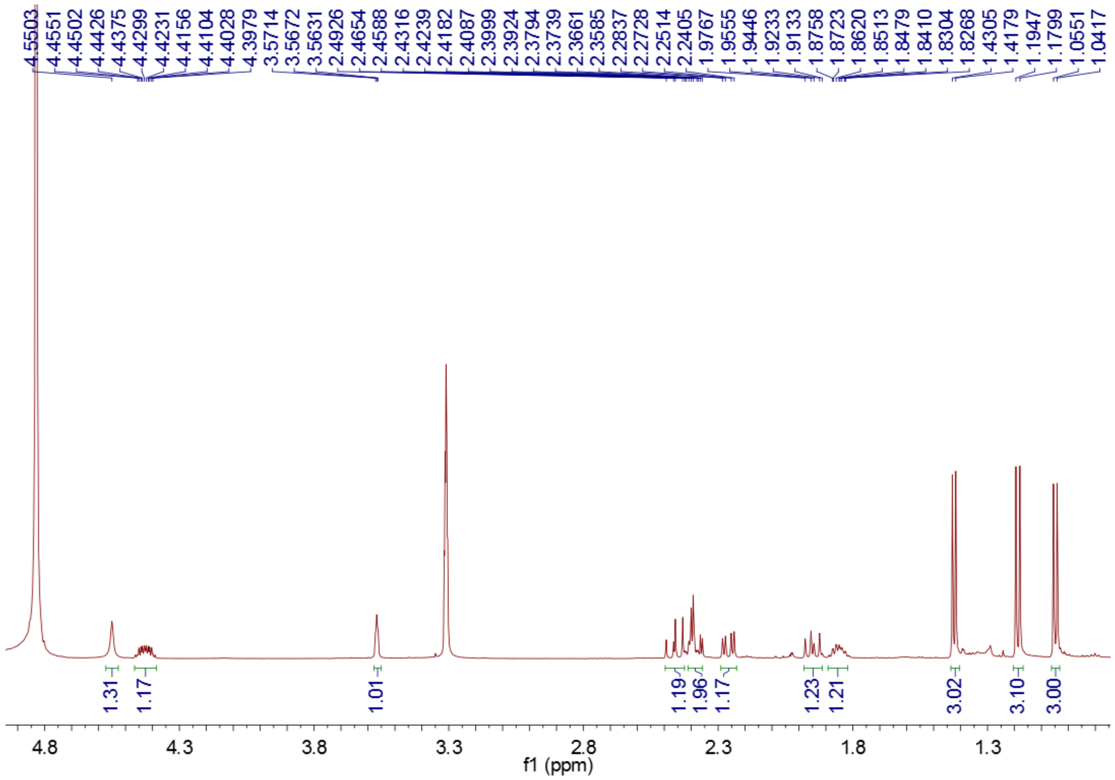

Figure S70. ^{1}H NMR spectrum (500 MHz, CD_3OD) of **8**.

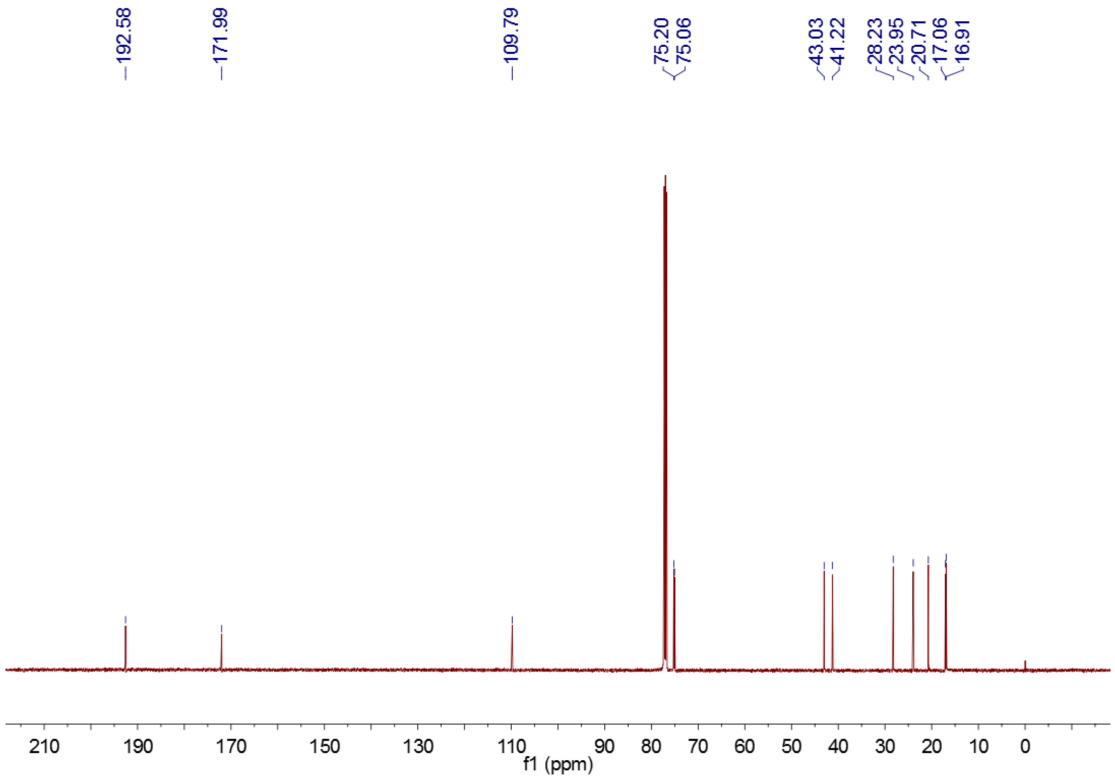

Figure S71. ^{13}C NMR spectrum (125 MHz, CD_3OD) of **8**.

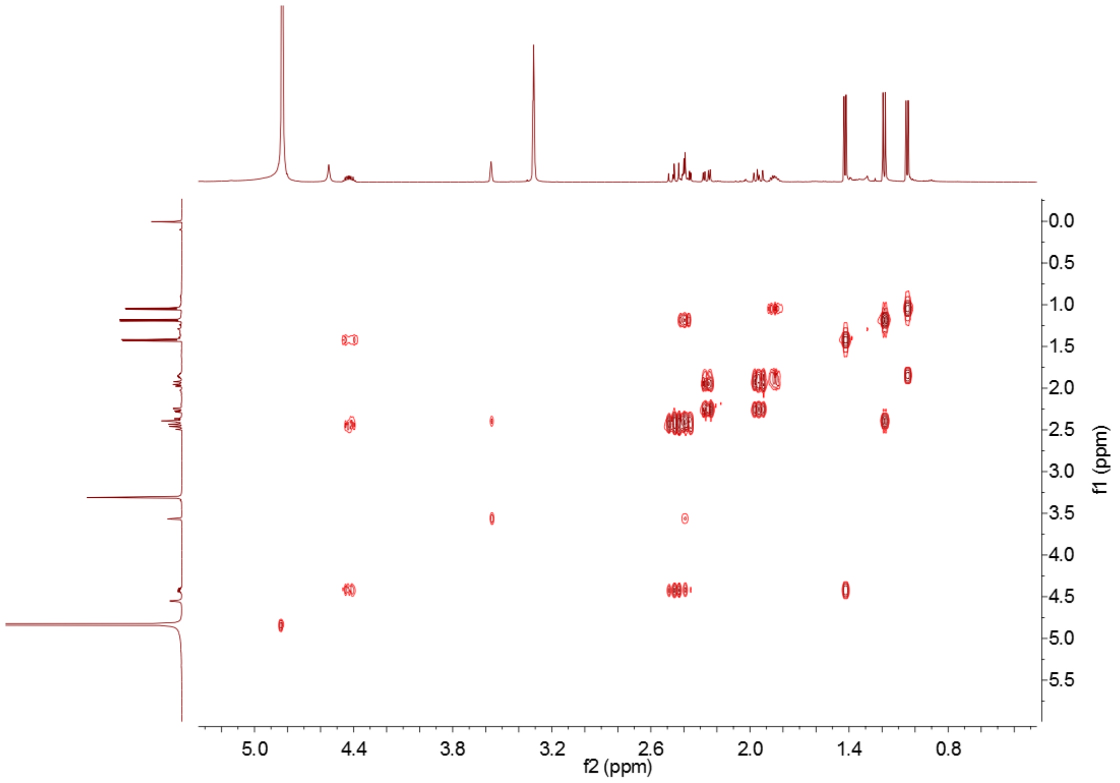

Figure S72. ^{1}H-^{1}H COSY spectrum of **8**.

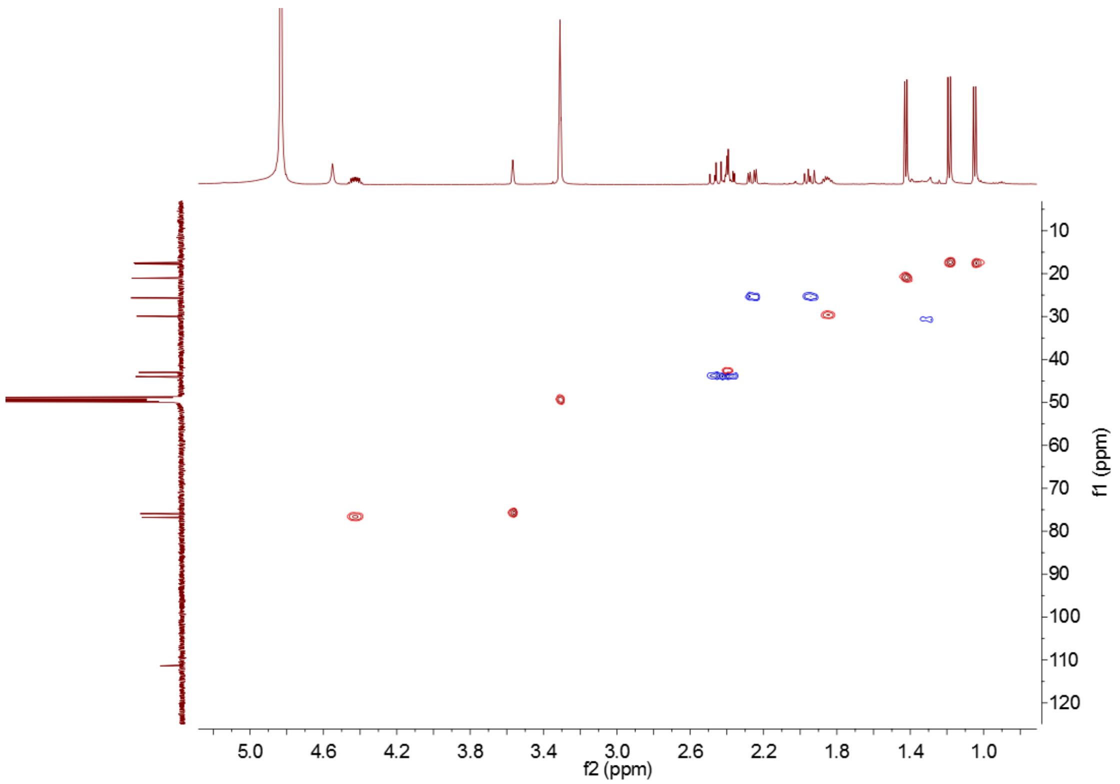

Figure S73. HSQC spectrum of **8**.

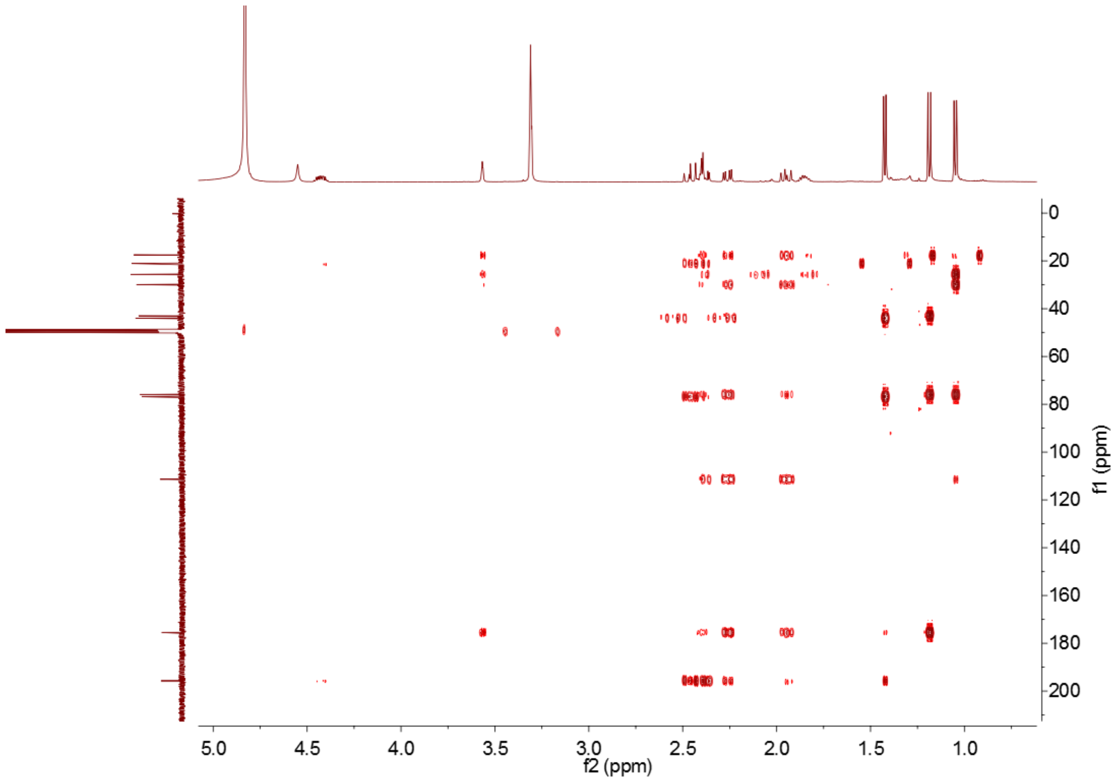

Figure S74. HMBC spectrum of **8**.

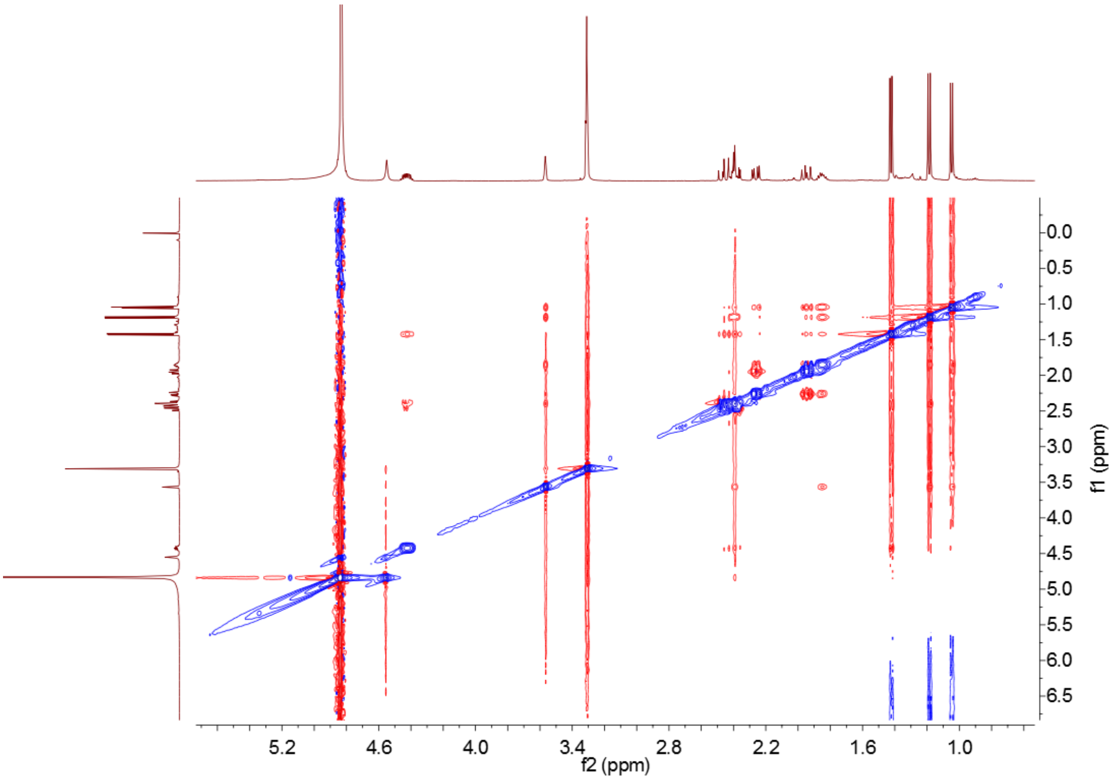

Figure S75. NOESY spectrum of **8**.

Mass Spectrum SmartFormula Report

**Analysis Info**		Acquisition Date	8/25/2020 11:34:24 AM
Analysis Name	D:\Data\MS\data\202008\liuxiuxiang_LWW-82_pos_77_01_8741.d		
Method	LC_Direct Infusion_pos_70-500mz.m	Operator	SCSIO
Sample Name	liuxiuxiang_LWW-82_pos	Instrument	maXis 255552.00029
Comment			

Acquisition Parameter

Source Type	ESI	Ion Polarity	Positive	Set Nebulizer	0.4 Bar
Focus	Active	Set Capillary	4500 V	Set Dry Heater	180 °C
Scan Begin	70 m/z	Set End Plate Offset	-500 V	Set Dry Gas	4.0 l/min
Scan End	1500 m/z	Set Charging Voltage	0 V	Set Divert Valve	Waste
		Set Corona	0 nA	Set APCI Heater	0 °C

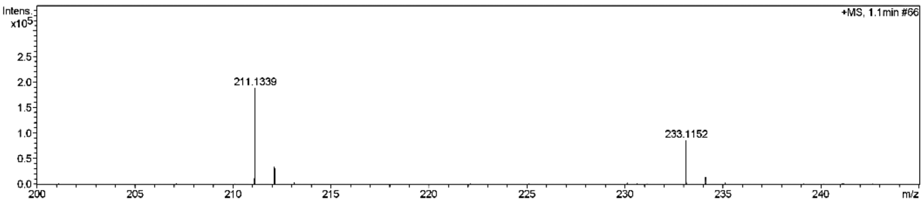

Meas. m/z	#	Ion Formula	Score	m/z	err [ppm]	err [mDa]	mSigma	rdb	e⁻ Conf	N-Rule
211.1339	1	C12H19O3	100.00	211.1329	-4.9	-1.0	27.8	3.5	even	ok
233.1152	1	C12H18NaO3	100.00	233.1148	-1.7	-0.4	19.6	3.5	even	ok
443.2405	1	C24H36NaO6	100.00	443.2404	-0.1	-0.0	42.2	6.5	even	ok

liuxiuxiang_LWW-82_pos_77_01_8741.d

Bruker Compass DataAnalysis 4.1 printed: 8/25/2020 11:39:11 AM by: SCSIO Page 1 of 1

Figure S76. HRESIMS spectrum of **8**.

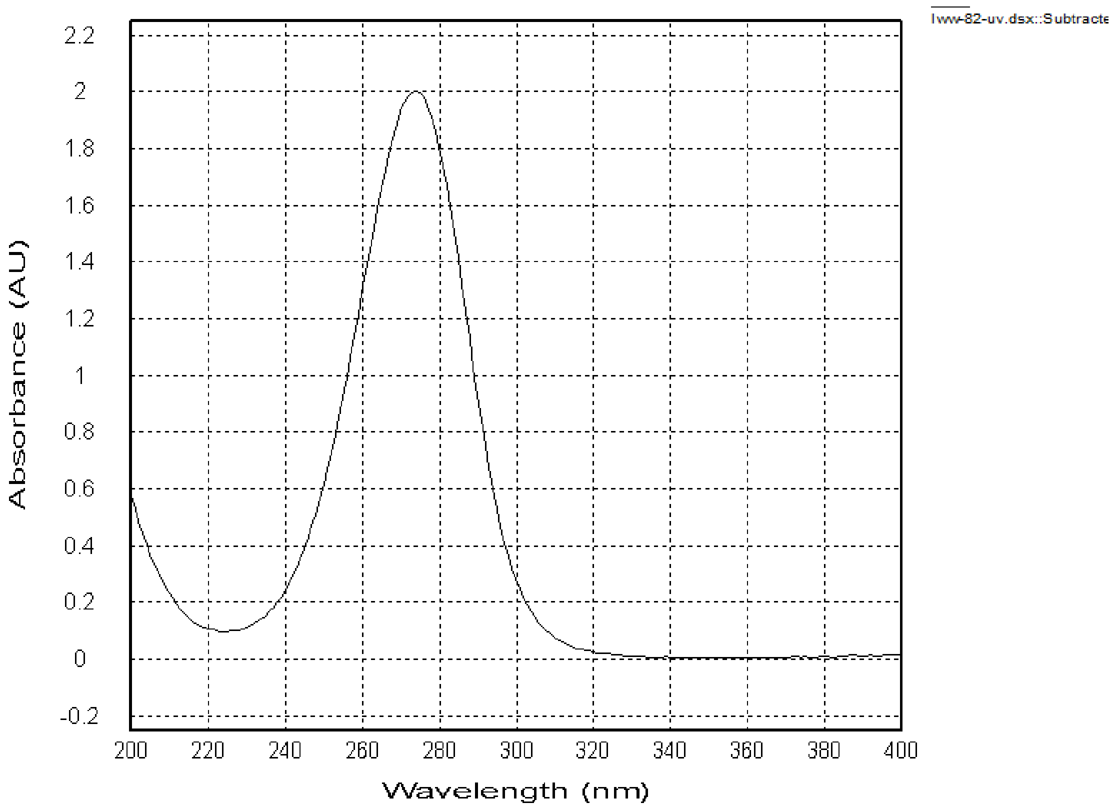

Figure S77. UV spectrum of **8**.

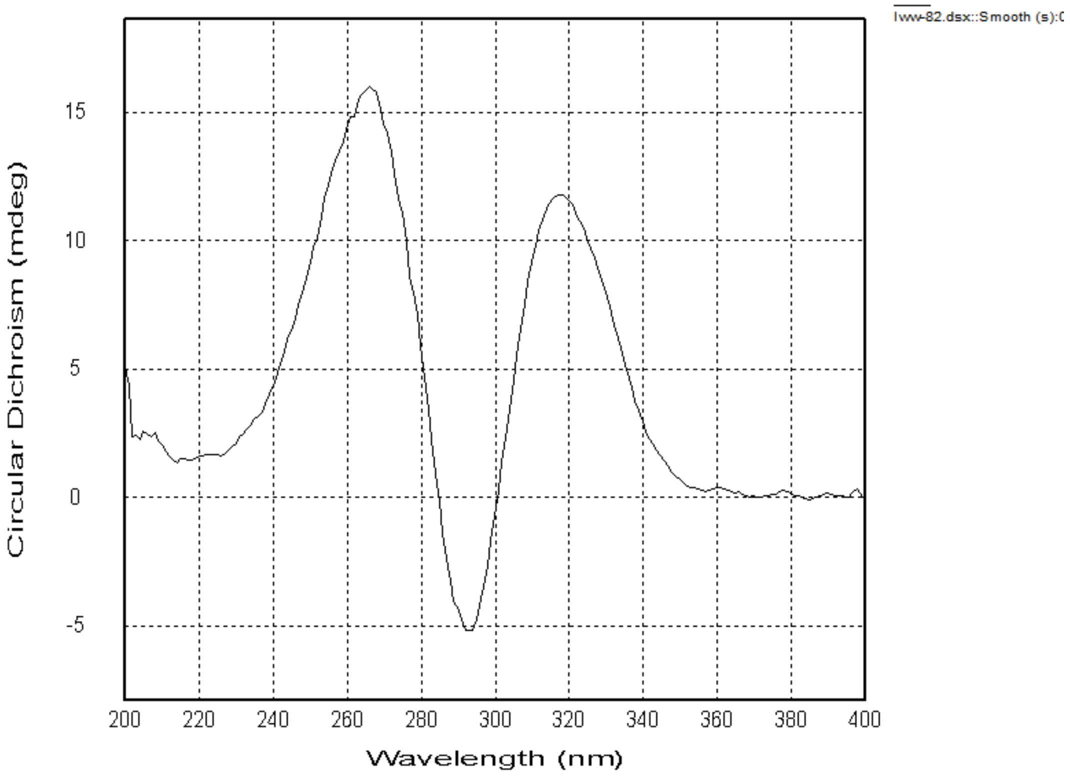

Figure S78. CD spectrum of **8**.

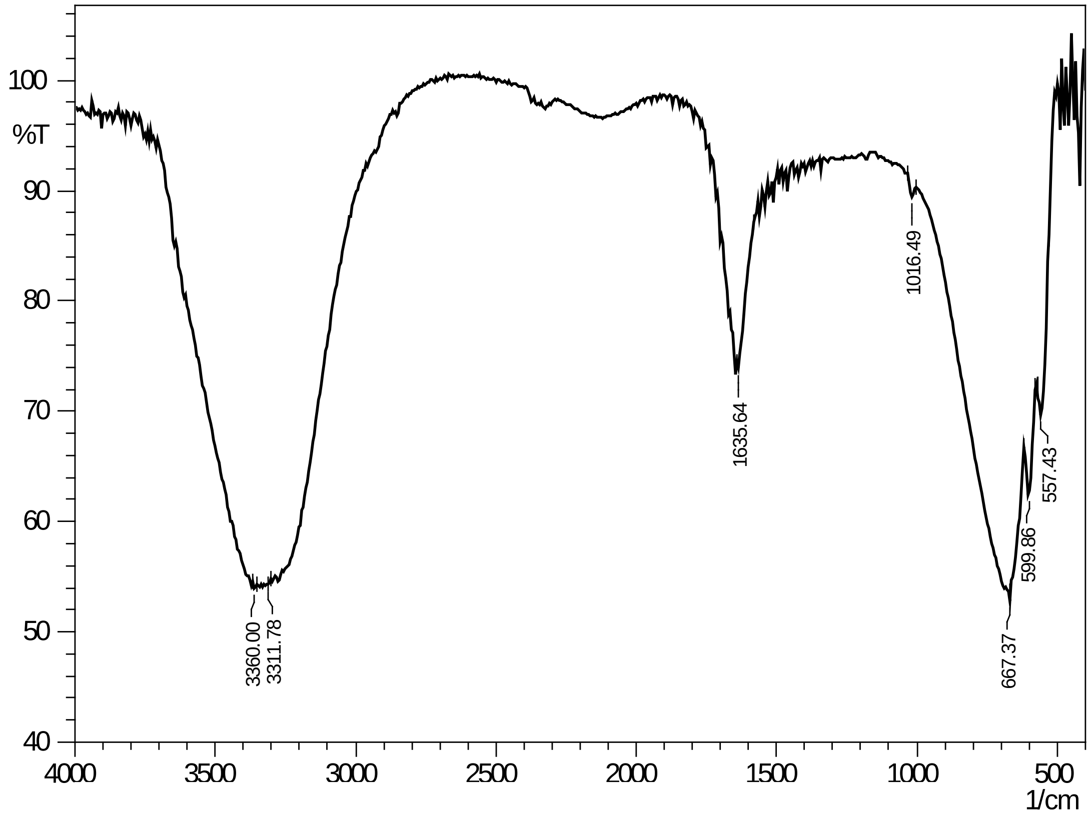

Figure S79. IR spectrum of **8**.

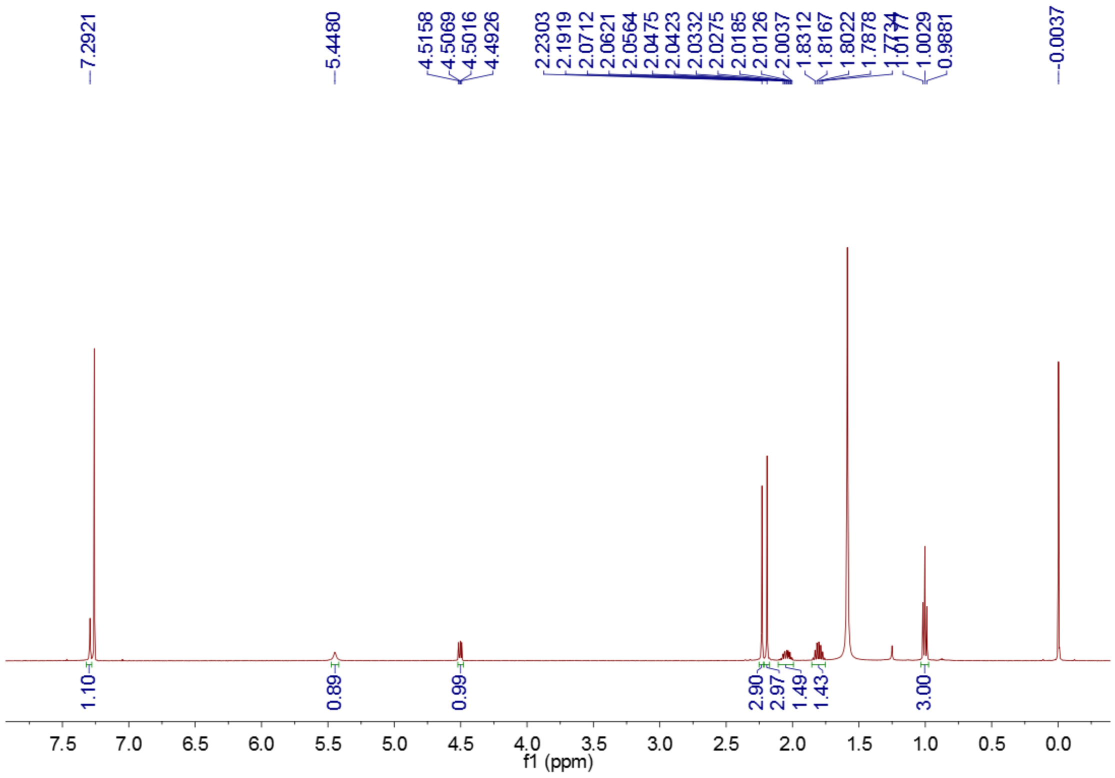

Figure S80. ^{1}H NMR spectrum (500 MHz, $CDCl_3$) of **9**.

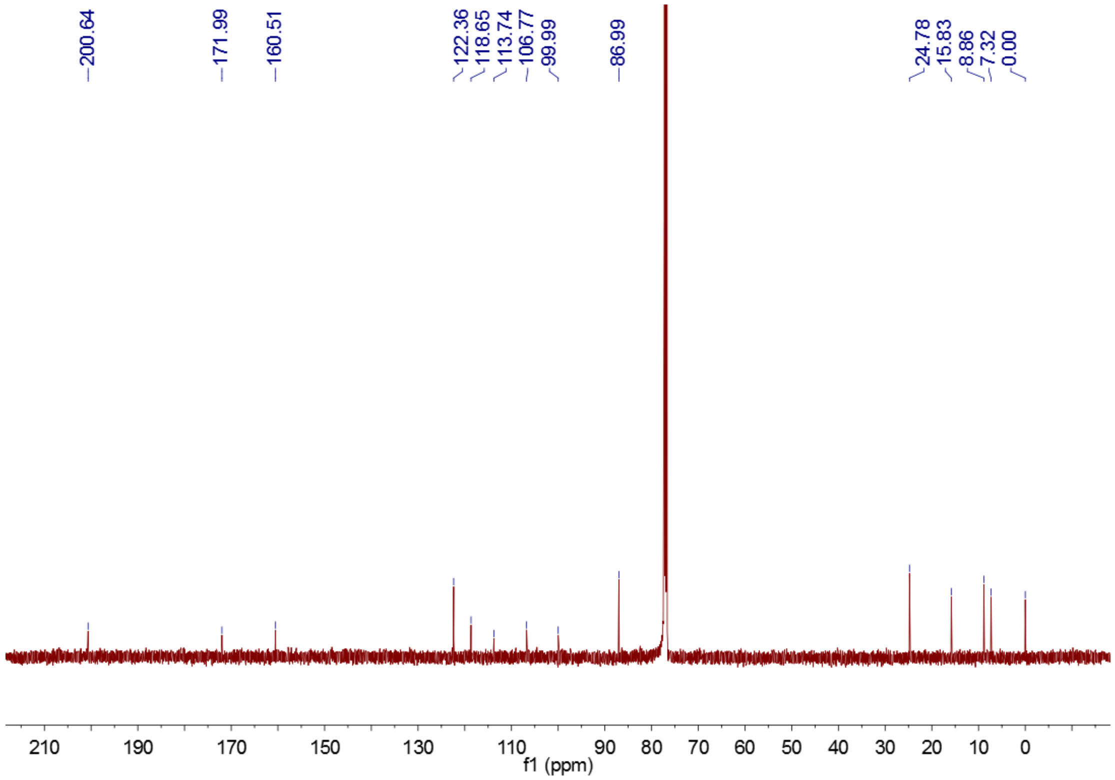

Figure S81. ^{13}C NMR spectrum (125 MHz, $CDCl_3$) of **9**.

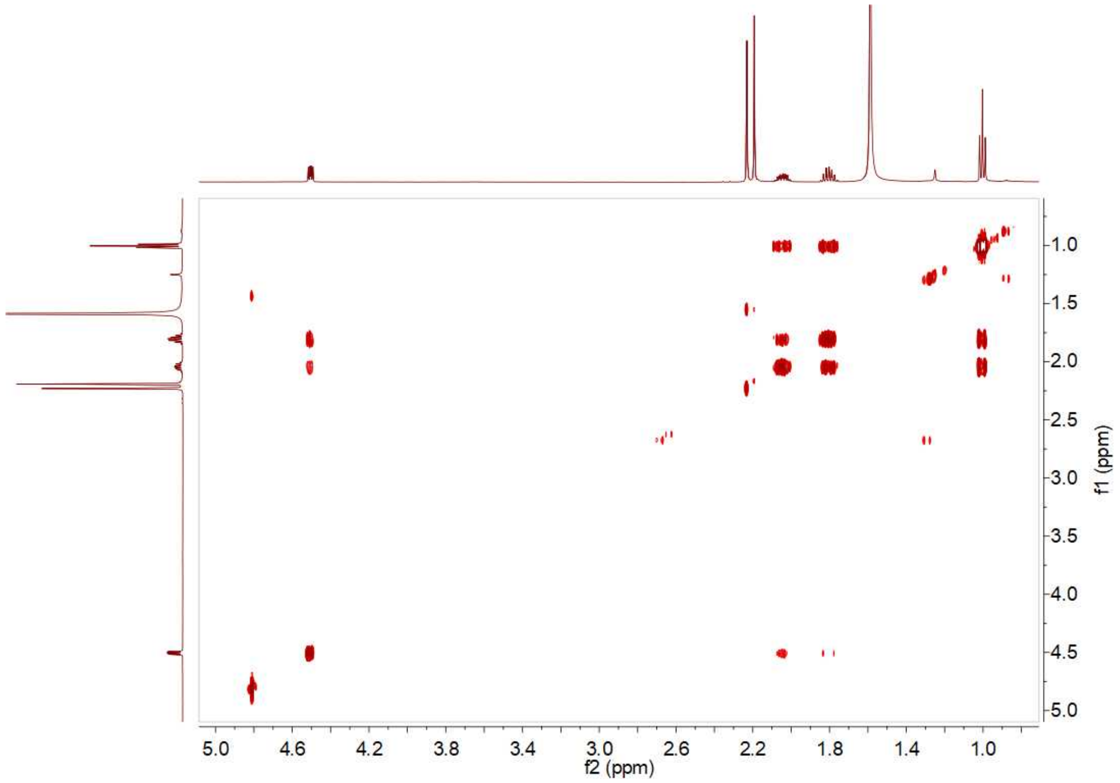

Figure S82. ^{1}H-^{1}H COSY spectrum of **9**.

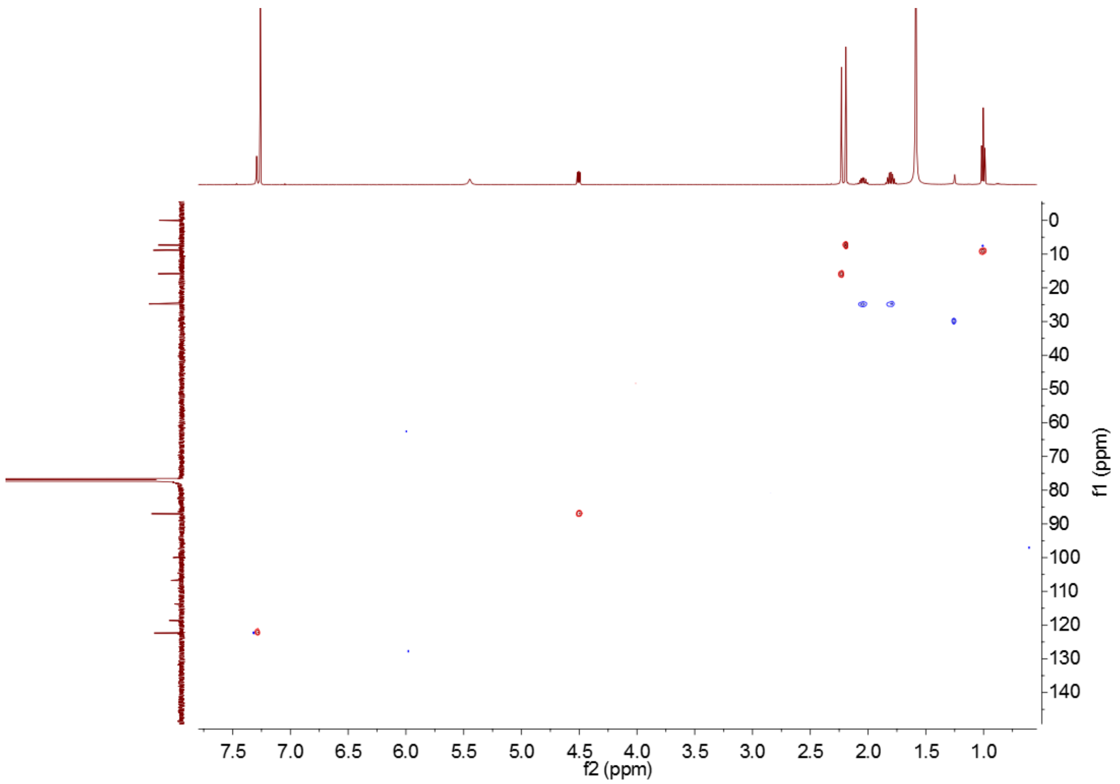

Figure S83. HSQC spectrum of **9**.

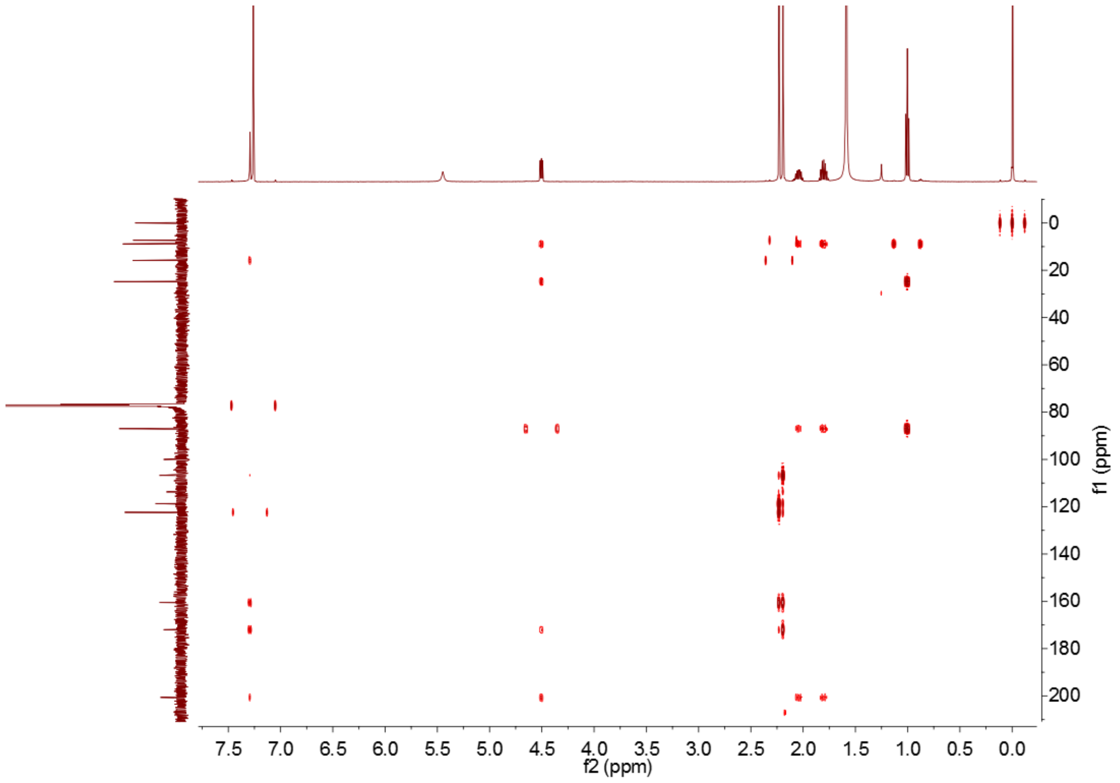

Figure S84. HMBC spectrum of **9**.

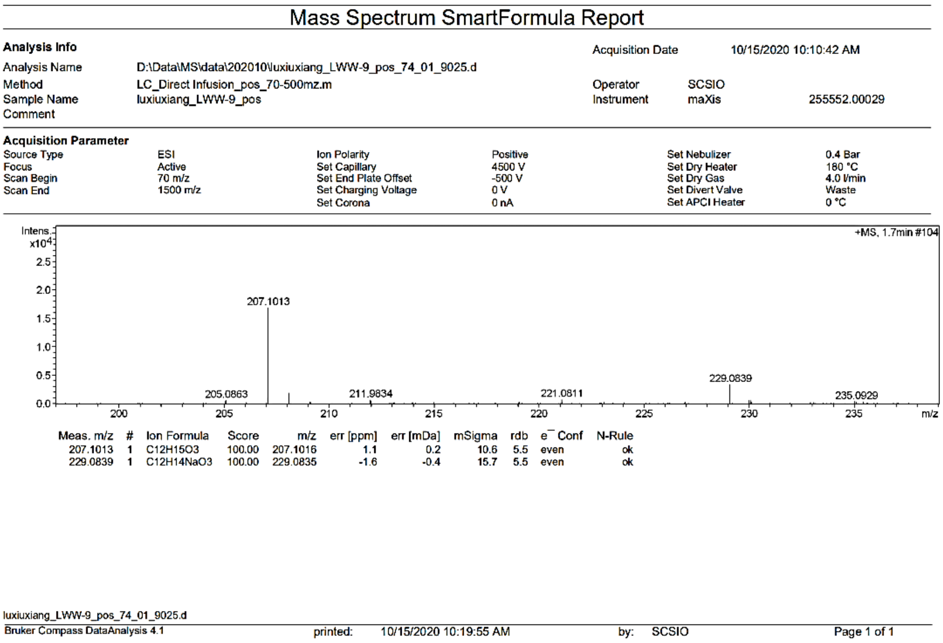

Figure S85. HRESIMS spectrum of **9**.

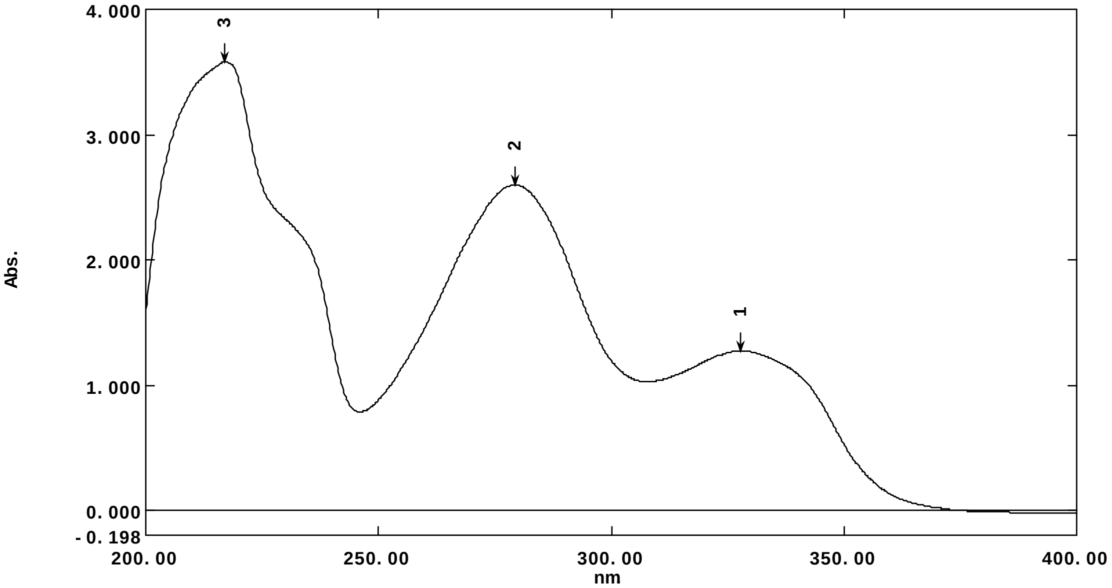

No	P/V	波长(nm)	吸收值
1		327.80	1.274
2		279.40	2.597
3		217.00	3.583

Figure S86. UV spectrum of **9.**

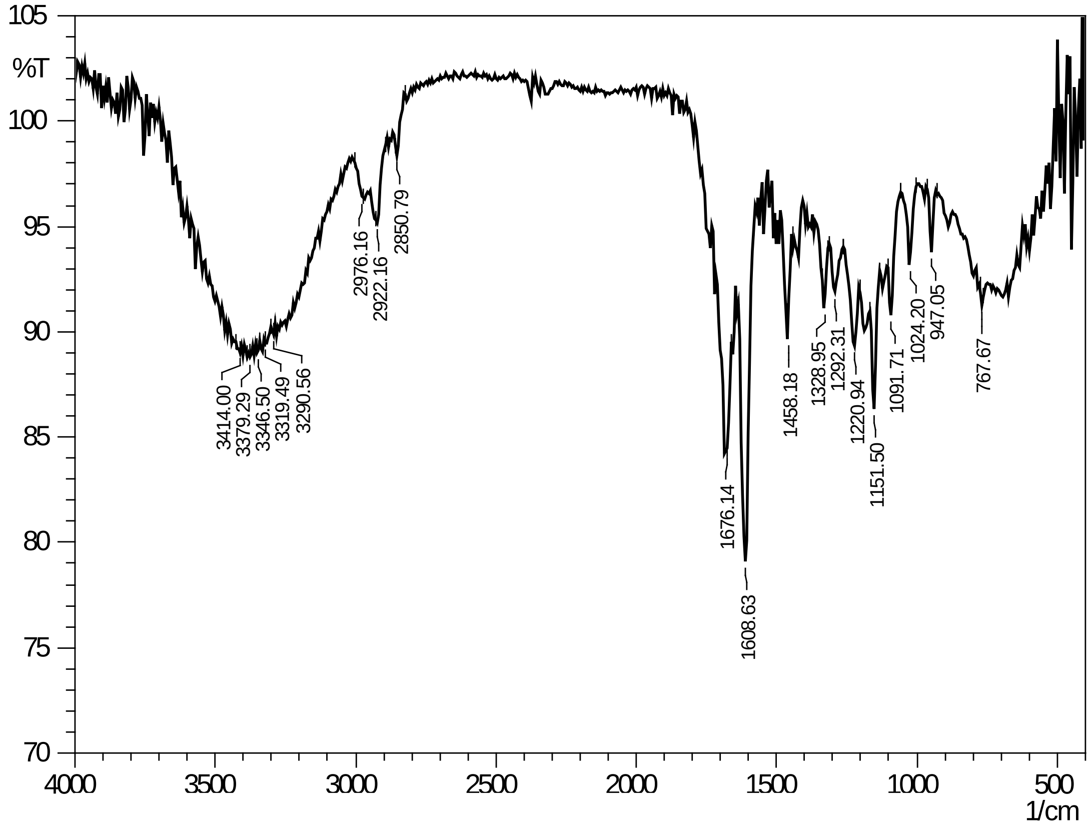

Figure S87. IR spectrum of **9**.

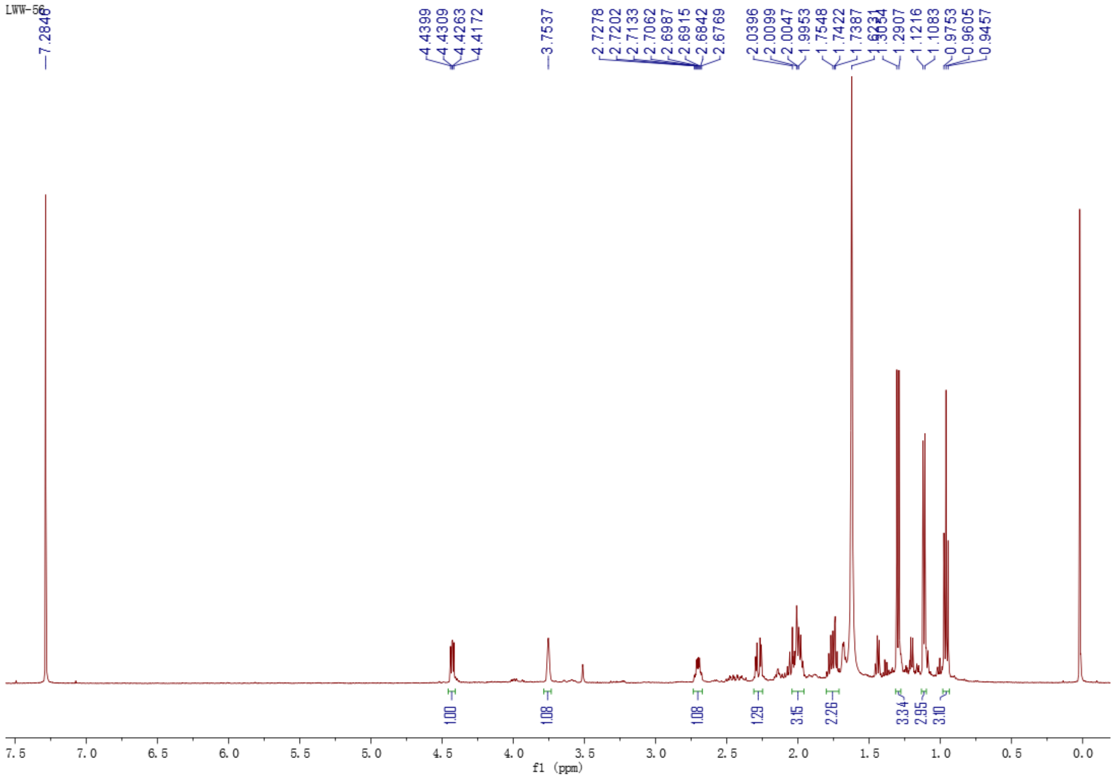

Figure S88. ^{1}H NMR spectrum (500 MHz, $CDCl_3$) of **10**.

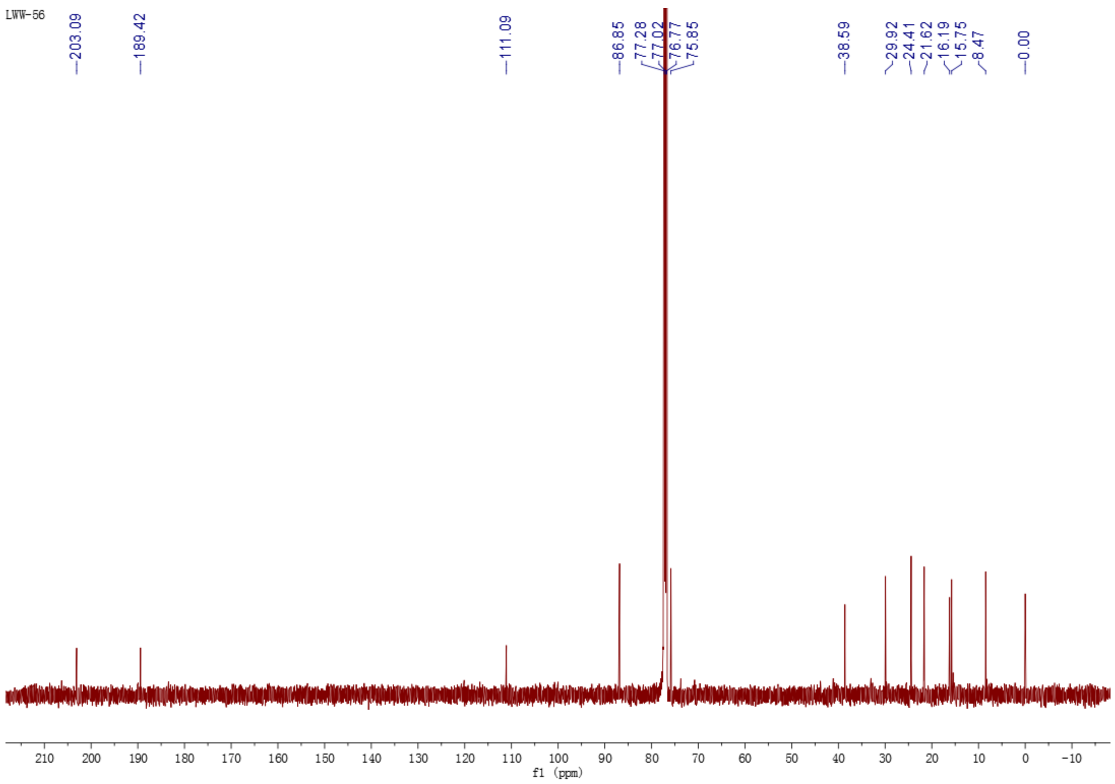

Figure S89. ^{13}C NMR spectrum (125 MHz, $CDCl_3$) of **10**.

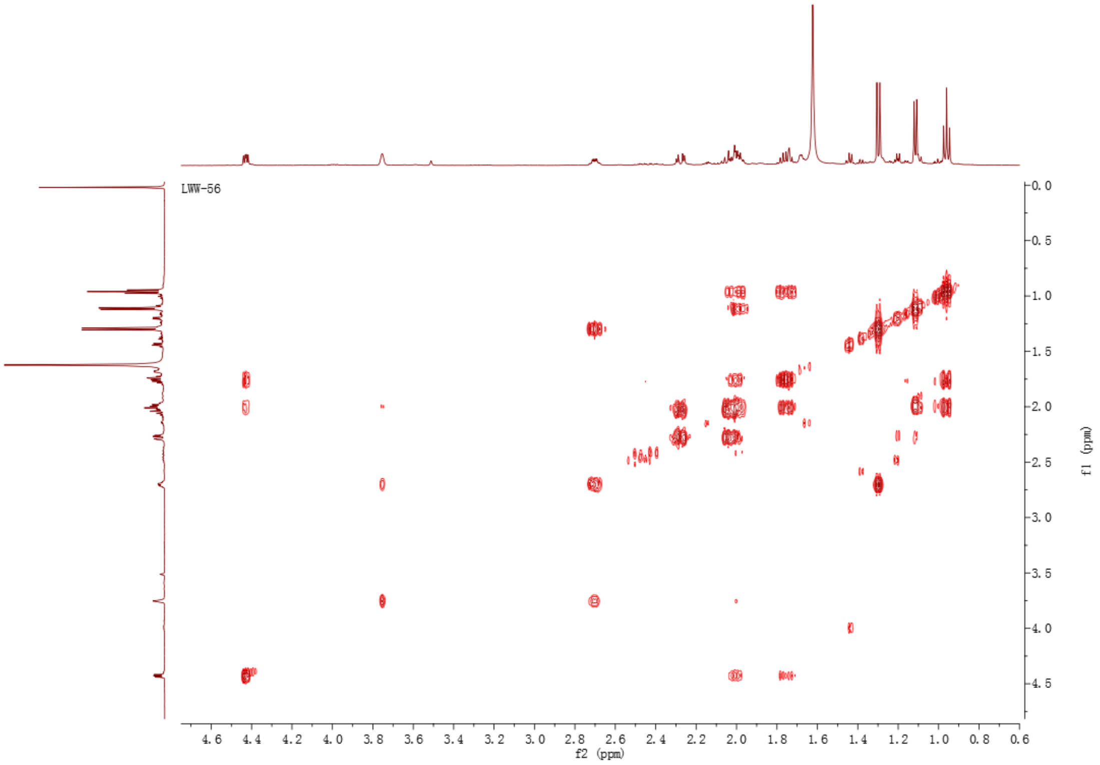

Figure S90. ^1H-^1H COSY spectrum of **10**.

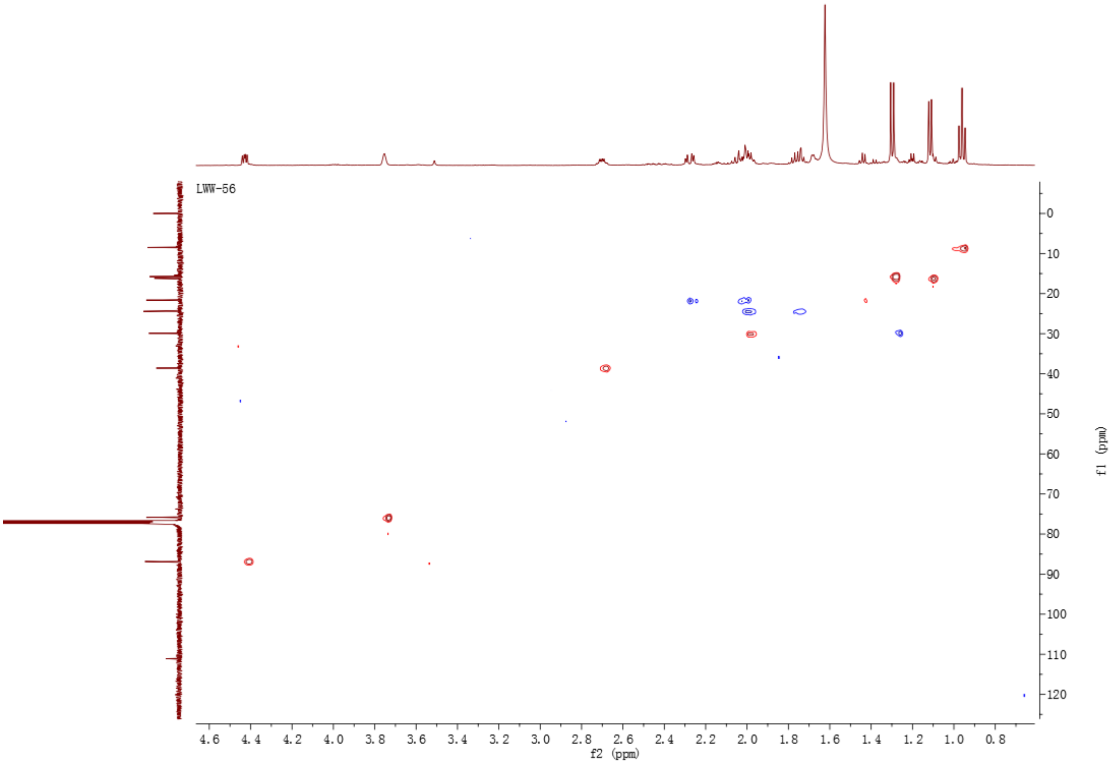

Figure S91. HSQC spectrum of **10**.

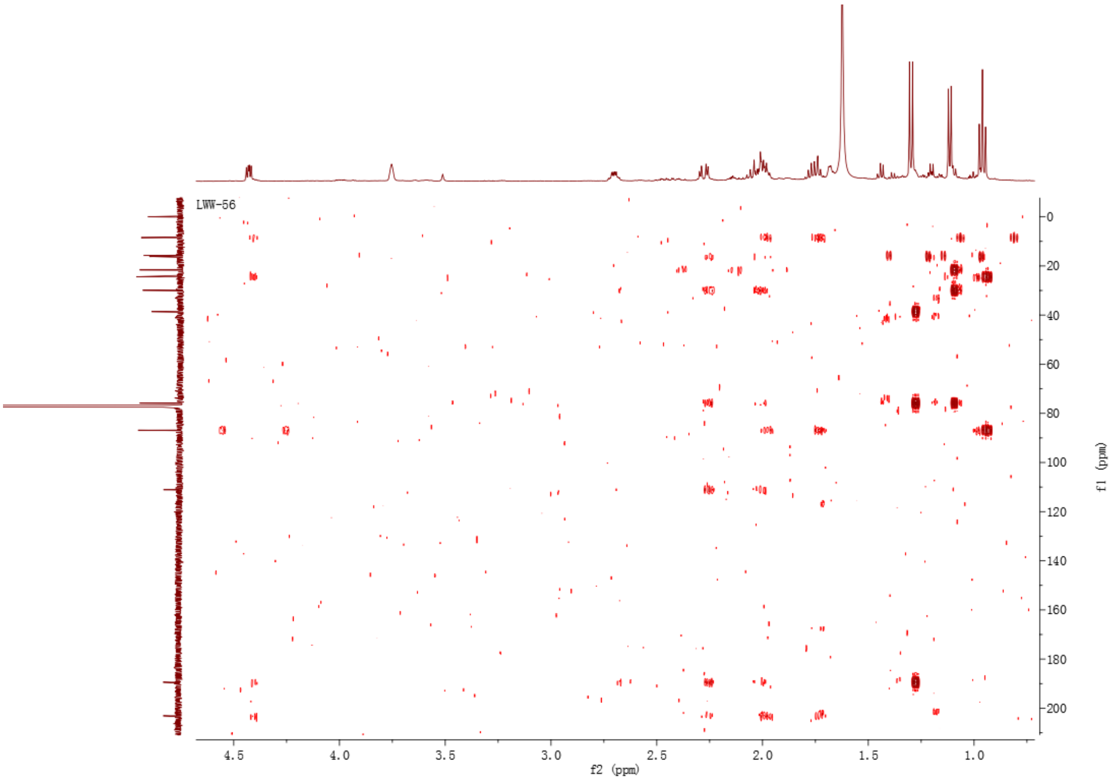

Figure S92. HMBC spectrum of **10**.

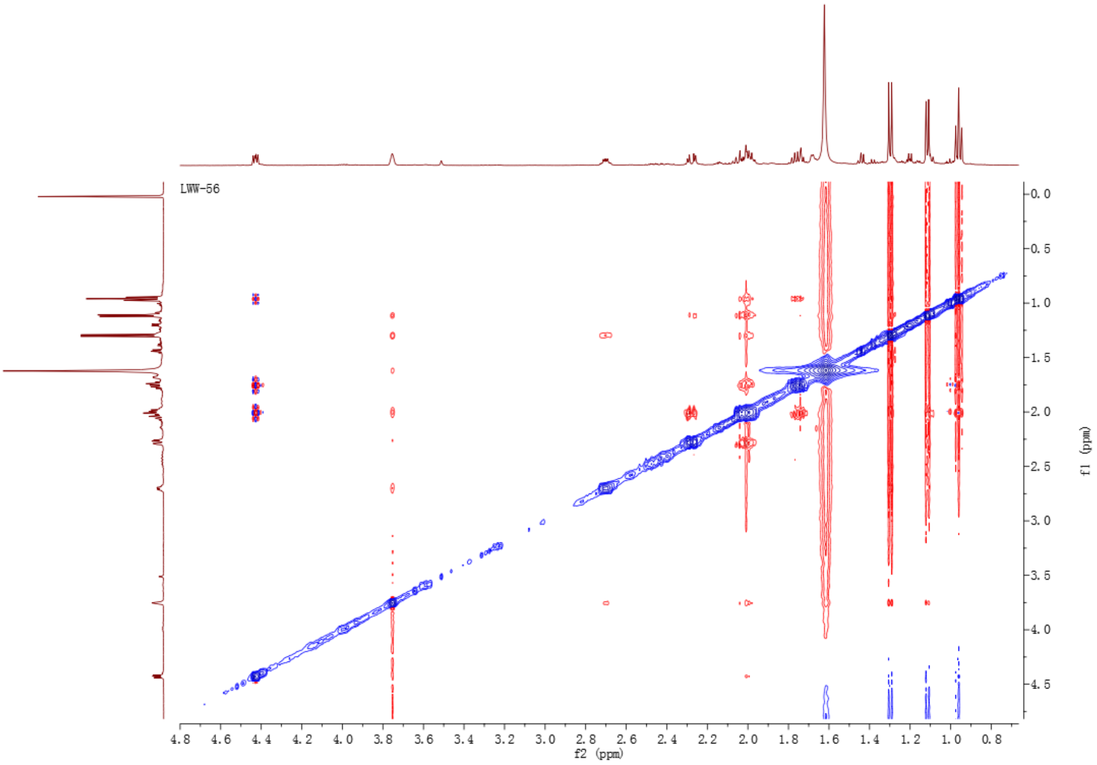

Figure S93. NOESY spectrum of **10**.

Mass Spectrum SmartFormula Report

Analysis Info

Analysis Name	D:\Data\MS\data\202008\liuxiuxiang_LWW-56_pos_76_01_8740.d	Acquisition Date	8/25/2020 11:30:50 AM	
Method	LC_Direct Infusion_pos_70-500mz.m	Operator	SCSIO	
Sample Name	liuxiuxiang_LWW-56_pos	Instrument	maXis	255552.00029
Comment				

Acquisition Parameter

Source Type	ESI	Ion Polarity	Positive	Set Nebulizer	0.4 Bar
Focus	Active	Set Capillary	4500 V	Set Dry Heater	180 °C
Scan Begin	70 m/z	Set End Plate Offset	-500 V	Set Dry Gas	4.0 l/min
Scan End	1500 m/z	Set Charging Voltage	0 V	Set Divert Valve	Waste
		Set Corona	0 nA	Set APCI Heater	0 °C

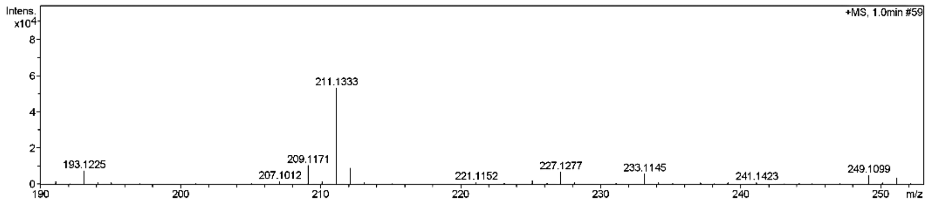

Meas. m/z	#	Ion Formula	Score	m/z	err [ppm]	err [mDa]	mSigma	rdb	e⁻ Conf	N-Rule
211.1333	1	C12H19O3	100.00	211.1329	-1.9	-0.4	20.0	3.5	even	ok
233.1145	1	C12H18NaO3	100.00	233.1148	1.2	0.3	10.8	3.5	even	ok

liuxiuxiang_LWW-56_pos_76_01_8740.d

Bruker Compass DataAnalysis 4.1 printed: 8/25/2020 11:36:24 AM by: SCSIO Page 1 of 1

Figure S94. HRESIMS spectrum of **10**.

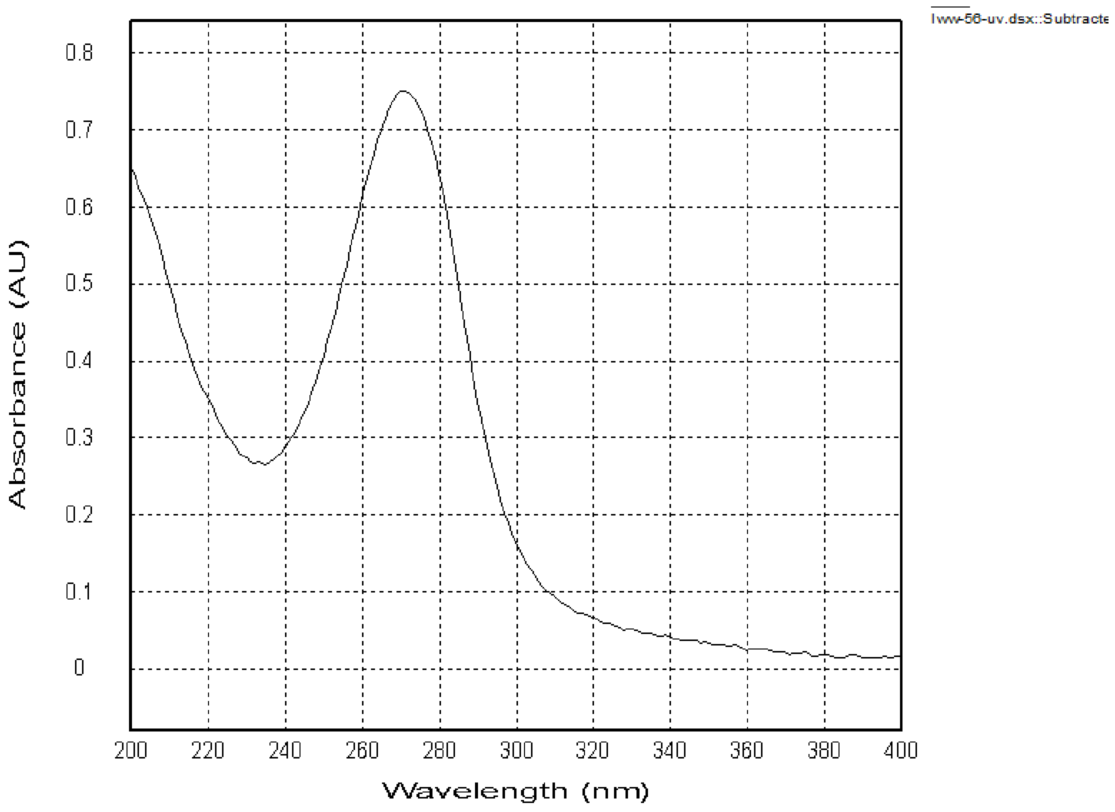

Figure S95. UV spectrum of **10.**

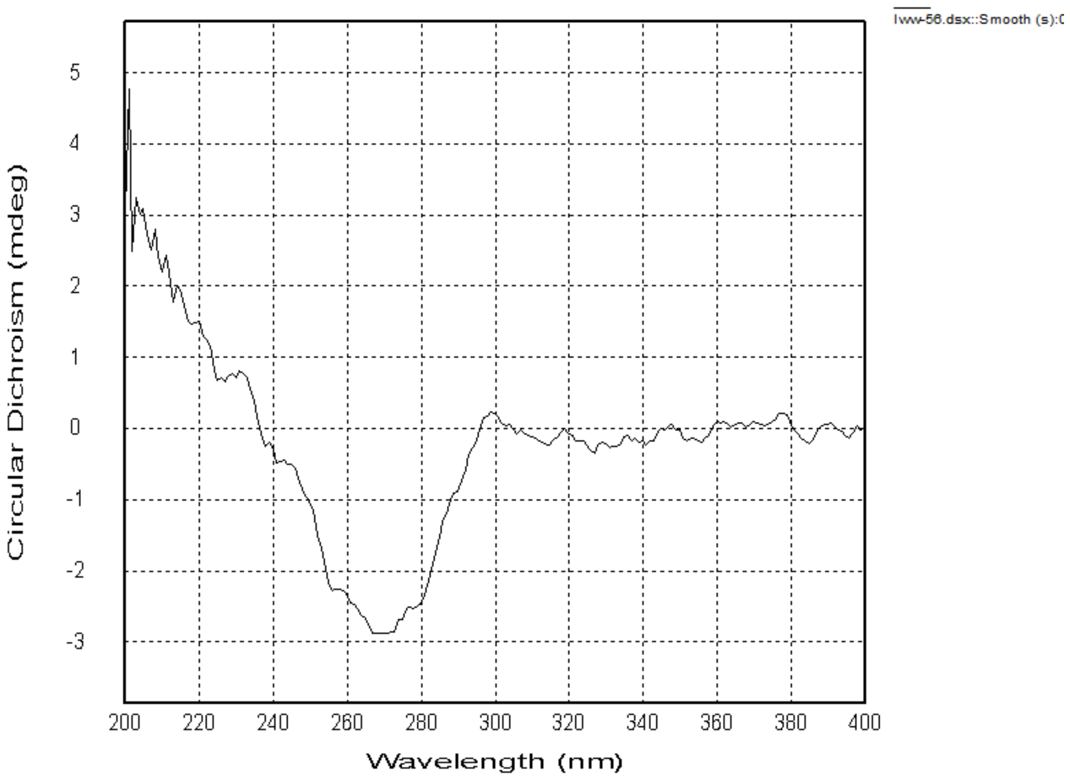

Figure S96. CD spectrum of **10**.

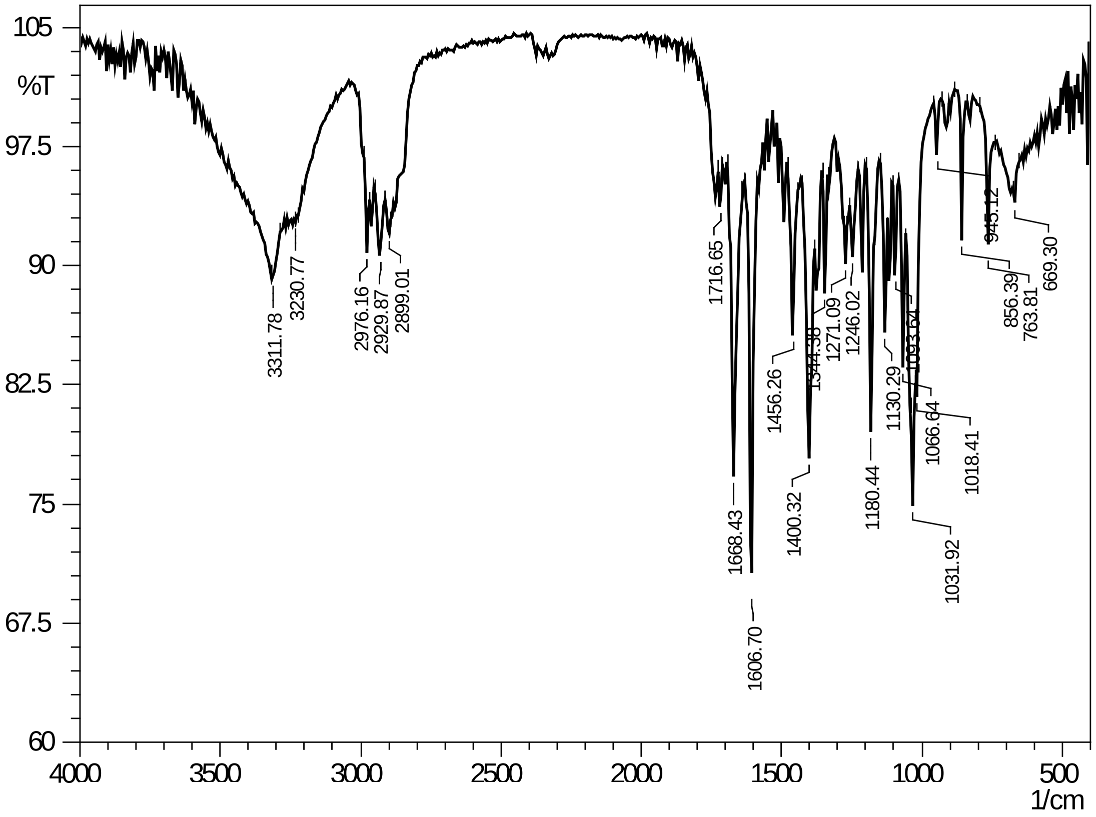

Figure S97. IR spectrum of **10**.

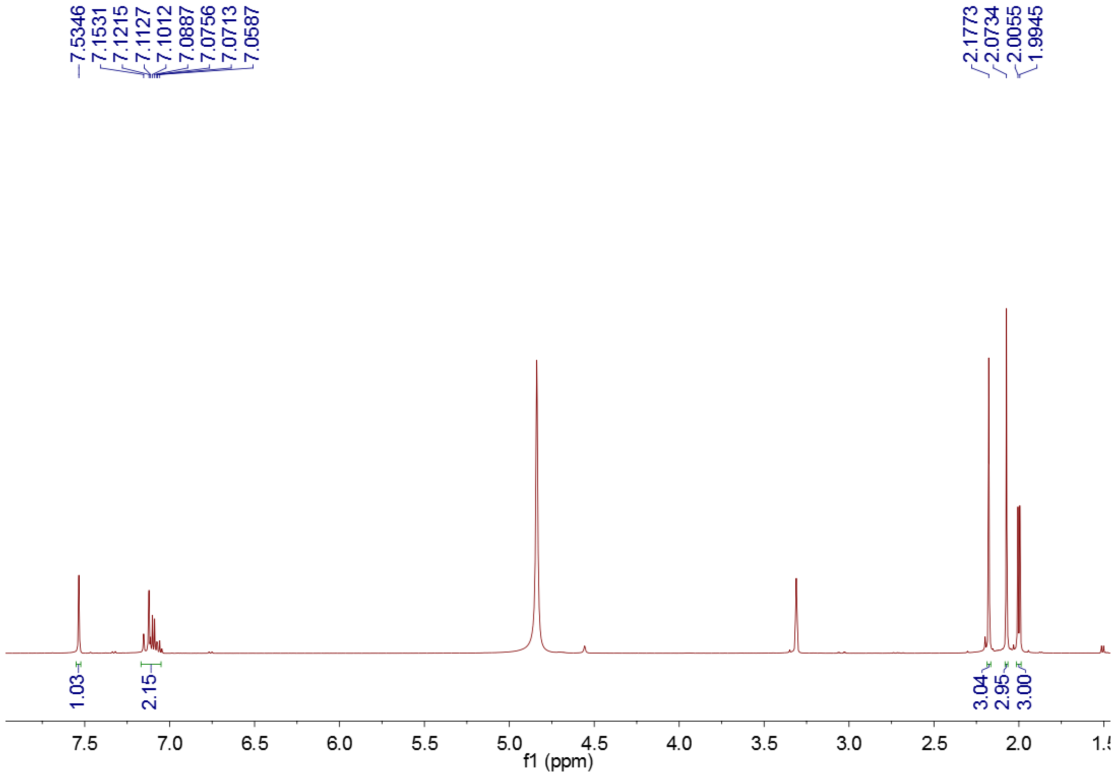

Figure S98. ^{1}H NMR spectrum (500 MHz, CD_3OD) of **11**.

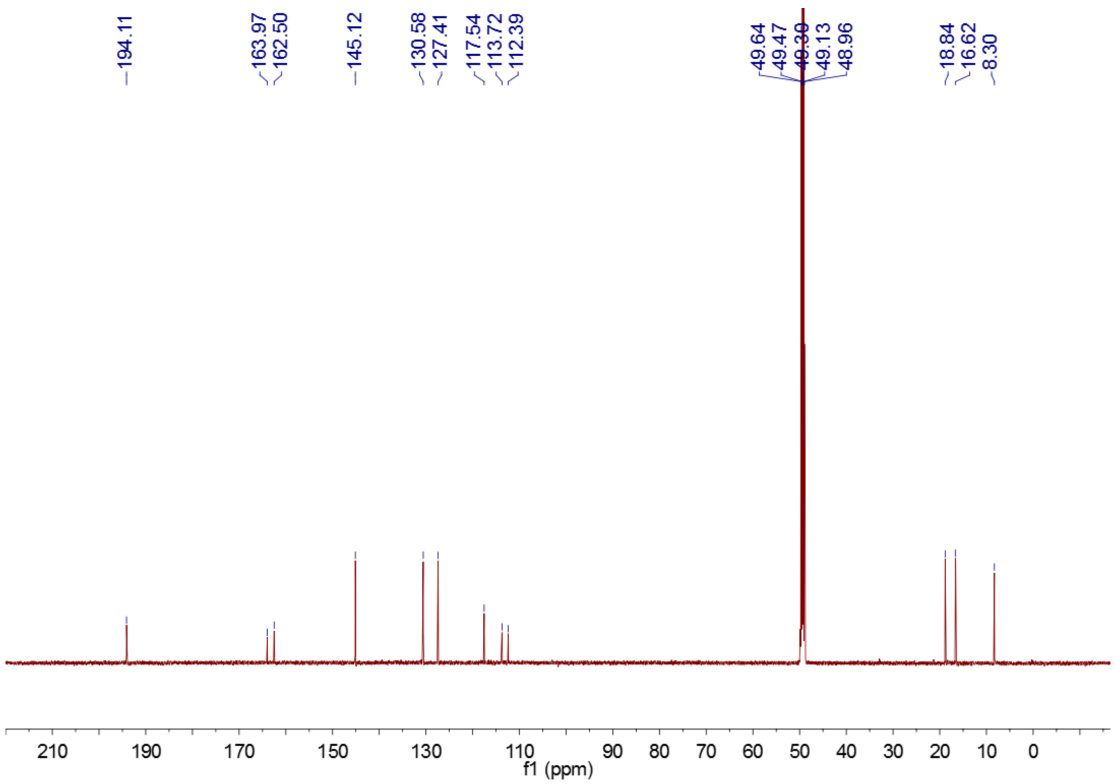

Figure S99. ^{13}C NMR spectrum (125 MHz, CD_3OD) of **11**.

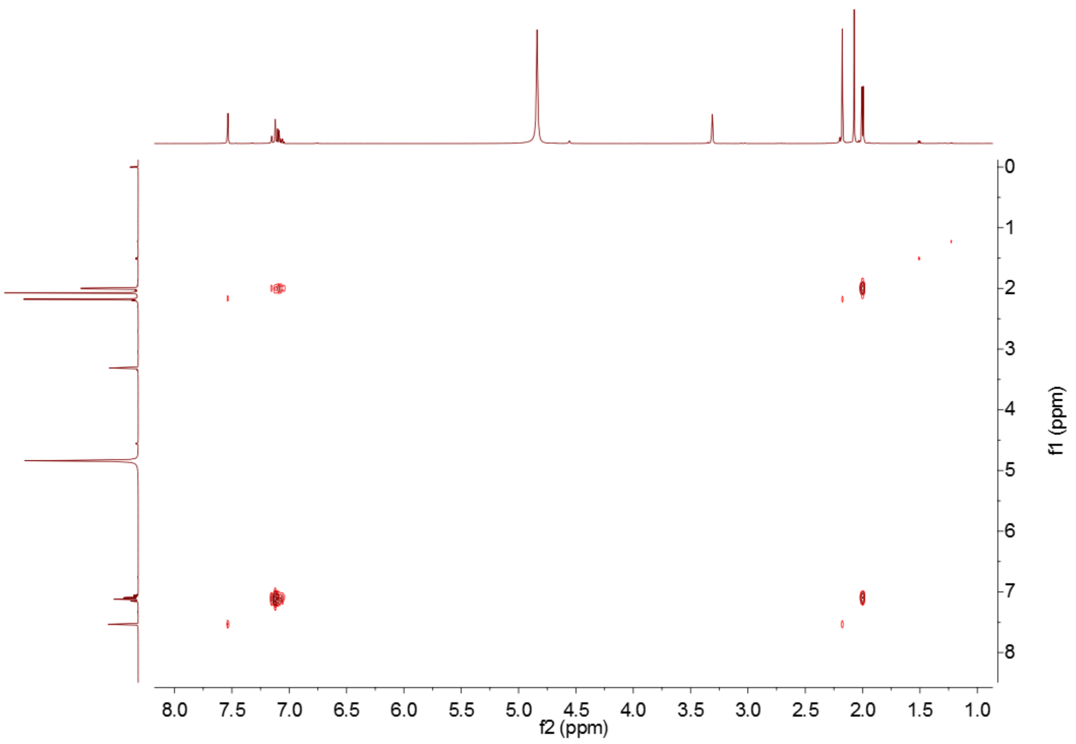

Figure S100. ^{1}H-^{1}H COSY spectrum of **11**.

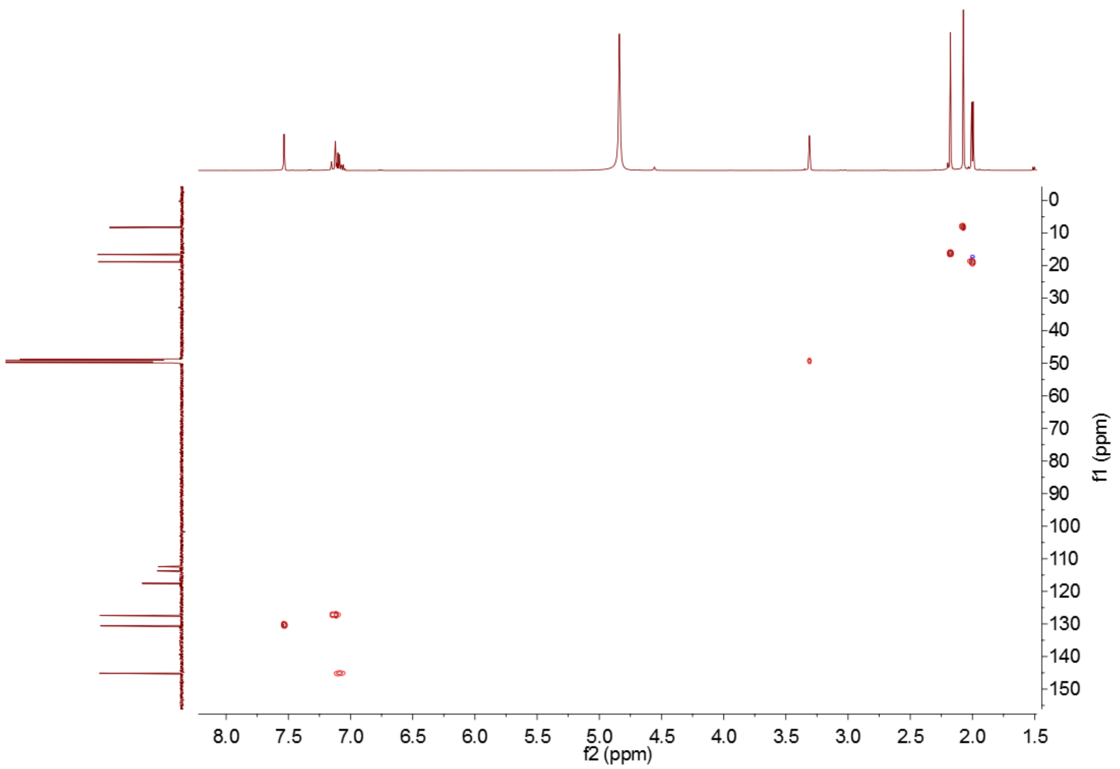

Figure S101. HSQC spectrum of **11**.

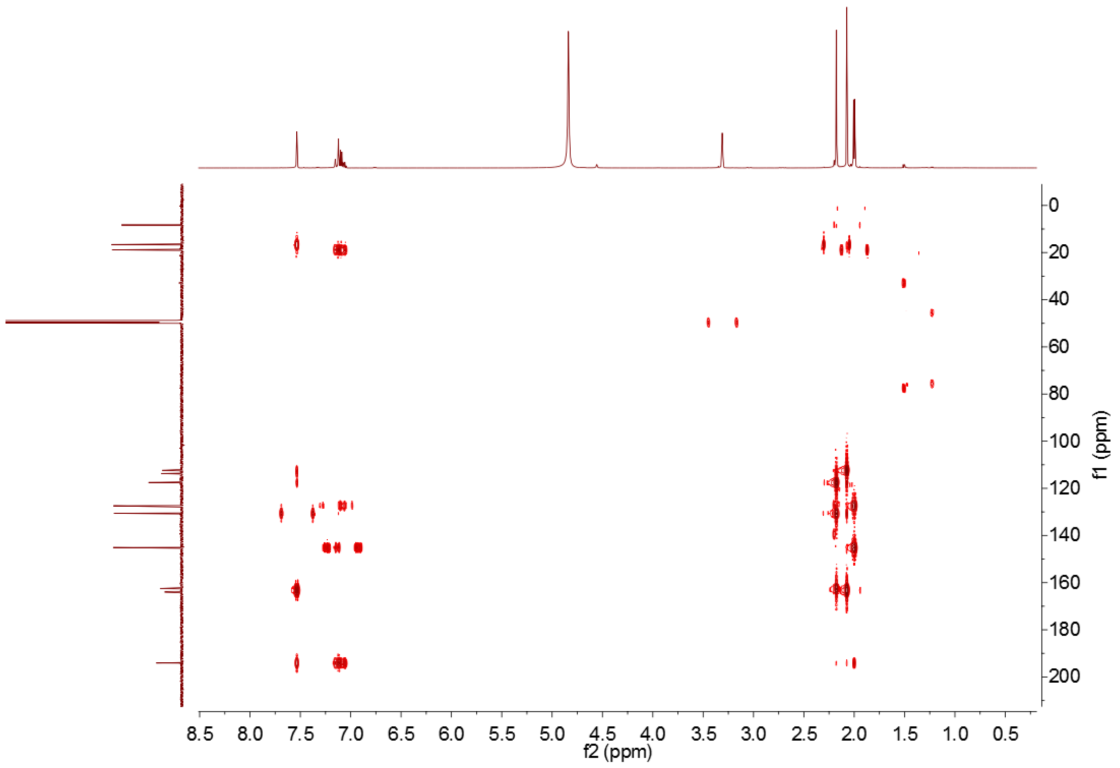

Figure S102. HMBC spectrum of **11**.

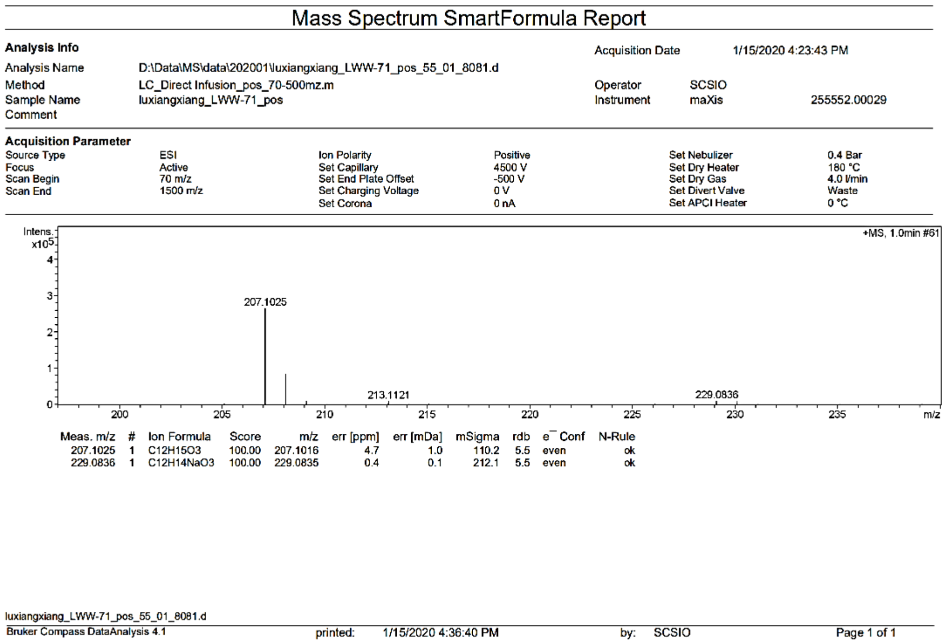

Figure S103. HRESIMS spectrum of **11**.

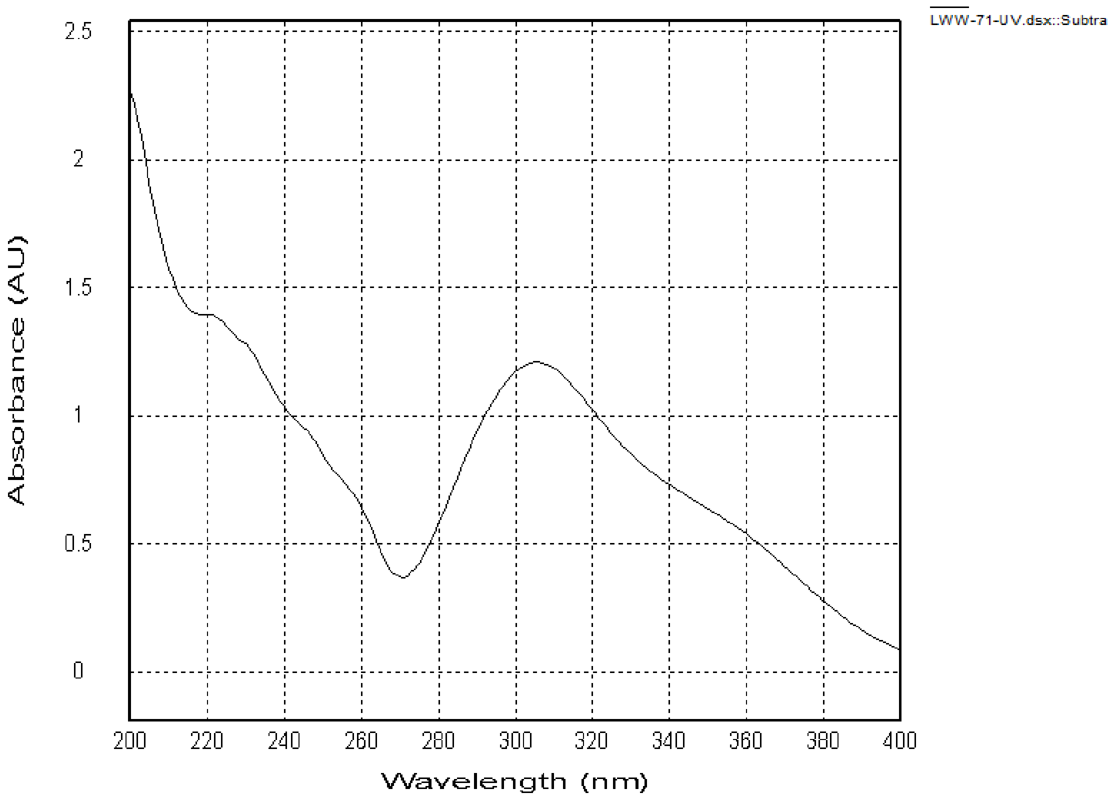

Figure S104. UV spectrum of **11.**

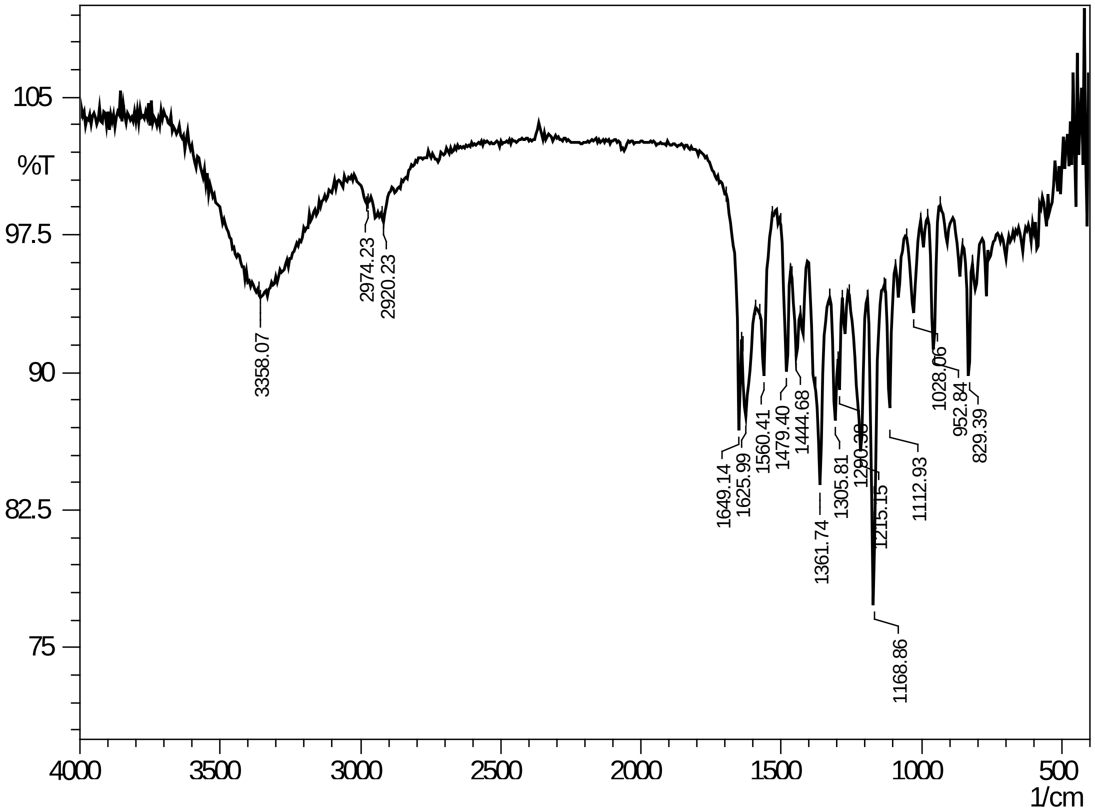

Figure S105. IR spectrum of **11**.
